# Entropy and the Second Law of Thermodynamics—The Nonequilibrium Perspective

**DOI:** 10.3390/e22070793

**Published:** 2020-07-21

**Authors:** Henning Struchtrup

**Affiliations:** Mechanical Engineering, University of Victoria, Victoria, BC V8W 2Y2, Canada; struchtr@uvic.ca

**Keywords:** 2nd law of thermodynamics, entropy, nonequilibrium, entropy generation, teaching thermodynamics

## Abstract

An alternative to the Carnot-Clausius approach for introducing entropy and the second law of thermodynamics is outlined that establishes entropy as a nonequilibrium property from the onset. Five simple observations lead to entropy for nonequilibrium and equilibrium states, and its balance. Thermodynamic temperature is identified, its positivity follows from the stability of the rest state. It is shown that the equations of engineering thermodynamics are valid for the case of local thermodynamic equilibrium, with inhomogeneous states. The main findings are accompanied by examples and additional discussion to firmly imbed classical and engineering thermodynamics into nonequilibrium thermodynamics.

## Highlights

- 2nd law and entropy based on 5 simple observations aligning with daily experience.
- Entropy defined for nonequilibrium and equilibrium states.
- Positivity of thermodynamic temperature ensures dissipation of kinetic energy, and stability of the rest state.
- Global balance laws based on assumption of local thermodynamic equilibrium.
- Agreement with Classical Equilibrium Thermodynamics and Linear Irreversible Thermodynamics.
- Entropy definition in agreement with Boltzmann’s H-function.
- Discussion of thermodynamic engines and devices, incl. Carnot engines and cycles, only *after* the laws of thermodynamics are established.

## Preamble

This text centers on the introduction of the 2nd Law of Thermodynamics from a small number of everyday observations. The emphasis is on a straight path from the observations to identifying the 2nd law, and thermodynamic temperature. There are only few examples or applications, since these can be found in textbooks. The concise presentation aims at the more experienced reader, in particular those that are interested to see how the 2nd law can be introduced without Carnot engines and cycles.

Nonequilibrium states and nonequilibrium processes are at the core of thermodynamics, and the present treatment puts these at center stage—where they belong. Throughout, all thermodynamic systems considered are allowed to be in nonequilibrium states, which typically are inhomogeneous in certain thermodynamic properties, with homogeneous states (for the proper variables) assumed only in equilibrium.

The content ranges from simple observation in daily life over the equations for systems used in engineering thermodynamics to the partial differential equations of thermo-fluid-dynamics; short discussions of kinetic theory of gases and the microscopic interpretation of entropy are included.

The presentation is split into many short segments to give the reader sufficient pause for consideration and digestion. For better flow, some material is moved to the Appendix which provides the more difficult material, in particular excursions into Linear Irreversible Thermodynamics and Kinetic Theory.

While it would be interesting to compare the approach to entropy presented below to what was or is done by others, this will not be done. My interest here is to give a rather concise idea of the introduction of entropy on the grounds of nonequilibrium states and irreversible processes, together with a smaller set of significant problems that can be tackled best from this viewpoint. The reader interested in the history of thermodynamics, and other approaches is referred to the large body of literature, of which only a small portion is referenced below.

## 1. Intro: What’s the Problem with the 2nd Law?

After teaching thermodynamics to engineering students for more than two decades (from teaching assistant to professor) I believe that a lot of the trouble, or the conceived trouble, with understanding of the 2nd law is related to how it is taught to the students. Most engineering textbooks introduce the 2nd law, and its main quantity, *Entropy*, following the classical Carnot-Clausius arguments [1] using reversible and irreversible engines [2,3,4,5]. To follow this approach properly, one needs a lot of background on processes, systems, property relations, engines and cycles. For instance in a widely adopted undergraduate textbook [2], the mathematical formulation of the 2nd law—the balance law for entropy—appears finally on page 380, more than 300 pages after the conservation law for energy, which is the mathematical expression of the *1st Law of Thermodynamics.*

Considering that the full discussion of processes, systems, property relations, engines and cycles requires *both*, the 1st *and* the 2nd law, it should be clear that a far more streamlined access to the body of thermodynamics can be achieved when both laws—in their full mathematical formulation—are available as early as possible. The doubtful reader will find some examples in the course of this treatise. Some authors are well aware of this advantage, which is common in Germany [6], but seems to come only slowly to the North-American market [7,8].

Early introduction of the 2nd law cannot be based on the Carnot-Clausius argument using engines and cycles, but must find other ways. In References [6,7] this problem is solved by simply postulating the 2nd law, and then showing first that is makes perfect sense, by analyzing simple systems, before using it to find less intuitive results, such as the limited efficiency of heat engines, and any other results of (applied) thermodynamics.

The problem with the postulative approach is that assumptions and restrictions of the equations are not always clearly stated. While the 1st law of thermodynamics is universally valid, this is not the case for most formulations of the 2nd law of thermodynamics. The presentation below aims at introducing the 2nd law step by step, with clear discussion of all assumptions used.

## 2. Outline: Developing the 2nd Law

Below, I will develop the 2nd law from a small number of observations. This approach is also outlined in my undergraduate textbook [8], but with some shortcuts and omissions that I felt are required for the target audience, that is, students who are exposed to the topic for the first time. The presentation below is aimed more at the experienced reader, therefore when it comes to the 2nd law, I aim to avoid these shortcuts, and discuss several aspects in more depth, in particular the all-important question of nonequilibrium states, while reducing the discussion of basic elements like properties and their relations as much as possible.

Many treatments of thermodynamics claim that it can only make statements on equilibrium states, and cannot deal with nonequilibrium states and irreversible processes. Would this be true, simple thermodynamic elements such as heat conductors or irreversible turbines could not be treated in the context of thermodynamics.

Indeed, classical [9,10,11] or more recent [12,13,14] treatments focus on equilibrium states, and avoid the definition of entropy in nonequilibrium.

However, in his famous treatment of kinetic theory of gases, Boltzmann gave proof of the H-theorem [15,16,17], which can be interpreted as the 2nd law of thermodynamics for ideal gases in *any* state. Entropy and its balance follow directly by integration from the particle distribution function and the Boltzmann equation, and there is no limitation at all to equilibrium states. Of course, for general nonequilibrium states one cannot expect to find a simple property relation for entropy, but this does not imply that entropy as an extensive system property does not exist. All frameworks of nonequilibrium thermodynamics naturally include a nonequilibrium entropy [18,19,20,21,22].

The arguments below focus on the approach to equilibrium from nonequilibrium states. Entropy is introduced as the property to describe equilibration processes, hence is defined for general nonequilibrium states.

Luckily, most engineering problems, and certainly those that are the typical topics in engineering thermodynamics, can be described in the framework of *local thermodynamic equilibrium* [18], where the well-known property relations for equilibrium are valid locally, but states are inhomogeneous (global equilibrium states normally are homogeneous in certain properties, see Section 40). Textbook discussions of open systems like nozzles, turbines, heat exchangers, and so forth, rely on this assumption, typically without explicitly stating this fact [2,3,4,5,6,7,8]. It is also worthwhile to note that local thermodynamic equilibrium arises naturally in kinetic theory, when one considers proper limitations on processes, viz. sufficiently small Knudsen number, see Appendix D.5 and References [16,17].

Obviously, the 2nd law does not exist on its own, that is for its formulation we require some background—and language—on systems, processes, and the 1st law, which will be presented first, as concisely as possible, before we will embark on the construction of the balance law for entropy, the property relations, and some analysis of its consequences. For completeness, short sections on *Linear Irreversible Thermodynamics* and *Kinetic Theory of Gases* are included in the Appendix.

For better accessibility, in most of the ensuing discussion we consider closed systems, the extension to open systems will be outlined only briefly. Moreover, mixtures, reacting or not, are not included, mainly for space reasons. Larger parts of the discussion, and the figures, are adapted from my textbook on technical thermodynamics [8], albeit suitably re-ordered, tailored, and re-formulated. The interested reader is referred to the book for many application to technical processes in open and closed systems, as well as the discussion of inert and reacting mixtures.

In an overview paper [13] of their detailed account of entropy as an equilibrium property [12], Lieb and Yngvason state in the subtitle that

*“The existence of entropy, and its increase, can be understood without reference to either statistical mechanics or heat engines.”*

This is the viewpoint of this contribution as well, hence I could not agree more. However, these authors’ restriction of entropy to equilibrium states is an unnecessary limitation. The analysis of irreversible processes is central in modern engineering thermodynamics, where systems in nonequilibrium states must be evaluated, and the entropy generation rate is routinely studied to analyze irreversible losses, and to redesign processes with the goal of entropy generation minimization [8,23]. Hence, while the goal appears to be the same, the present philosophy differs substantially from authors who restrict entropy to equilibrium states.

## 3. The 2nd Law in Words

In the present approach, the 2nd law summarizes everyday experiences in a few generalized observations which are then used to conclude on its mathematical formulation, that is, the balance law for entropy. Only the mathematical formulation of thermodynamics, which also includes the 1st law and property relations, allows to generalize from simple experience to the unexpected.

Below, the observations used, and the mathematical formulation of the 2nd law, are summarized as an introduction of what will come for the thermodynamically experienced reader, and for future reference for the newcomer. The definitions of terms used, explanations of the observations, and their evaluation will be presented in subsequent sections

The observation based form of the 2nd law reads:

**Basic Observations**

**Observation** **1.** *A closed system can only be manipulated by heat and work transfer.*
**Observation** **2.** *Over time, an isolated thermodynamic system approaches a unique and stable equilibrium state.*
**Observation** **3.** *In a stable equilibrium state, the temperature of a thermally unrestricted system is uniform.*
**Observation** **4.** *Work transfer is unrestricted in direction, but some work might be lost to friction.*
**Observation** **5.** *Heat will always go from hot to cold by itself, but not vice versa.*

The experienced reader should not be surprised by any of these statements. The second, third and fifth probably are most familiar, since they appear as input, or output, in any treatment of the 2nd law. The first and the fourth are typically less emphasized, if stated at all, but are required to properly formulate the transfer of entropy, and to give positivity of thermodynamic temperature.

Careful elaboration will show that these observations, with the assumption of local thermodynamic equilibrium, will lead to the 2nd law for closed systems in the form

(1) $$\frac{dS}{dt}-\sum_{k}\frac{{\dot{Q}}_{k}}{T_{k}}={\dot{S}}_{gen}\geq 0\;\;\;\;\mathrm{and}\;\;\;\;T>0\;.$$

Here, *S* is the concave entropy of the system, ${\dot{Q}}_{k}$ is energy transfer by heat over the system boundary at positive thermodynamic temperature $T_{k}$, and ${\dot{S}}_{gen}$ is the non-negative generation rate of entropy within the system, which vanishes in equilibrium.

## 4. Closed System

The first step in any thermodynamic consideration is to identify the system that one wishes to describe. Any complex system, for example, a power plant, can be seen as a compound of some—or many—smaller and simpler systems that interact with each other. For the basic understanding of the thermodynamic laws it is best to begin with the simplest system, and study more complex systems later as assemblies of these simple systems.

**Figure 1:**
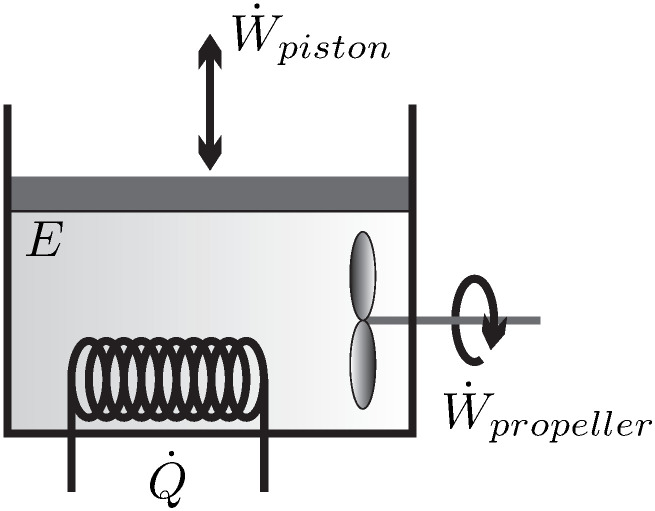
Closed system with energy *E* exchanging work $\dot{W}$ and heat $\dot{Q}$ with its surroundings.

The simplest system of interest, and the one we will consider for most of the discussion, is the*closed system* where a simple substance (i.e., no chemical changes) is enclosed by walls, and no mass flows over the system boundaries, for example, the piston-cylinder device depicted in Figure 1.

There is only a small number of manipulations possible to change the state of a closed system, which are indicated in the figure—the volume of the system can be changed by moving the piston, the system can be stirred with a propeller, and the system can be heated or cooled by changing the temperature of the system boundary, as indicated by the heating (or cooling) coil. Another possibility to heat or cool the system is through absorption and emission of radiation, and transfer of radiation across the system boundary (as in a microwave oven)–this is just another way of heating. One could also shake the system, which is equivalent to stirring.

The statement that there is no other possible manipulation of the system than these is formulated in **Observation 1**.

These manipulative actions lead to exchange of energy between the system and its surroundings, either by work in case of piston movement and stirring, or by the exchange of heat. The transfer of energy (*E*) by work ($\dot{W}$) and heat ($\dot{Q}$) will be formulated in the *1st Law of Thermodynamics* (Section 12). The fundamental difference between piston and propeller work, as (possibly) reversible and irreversible processes will become clear later.

## 5. Properties

**Figure 2:**
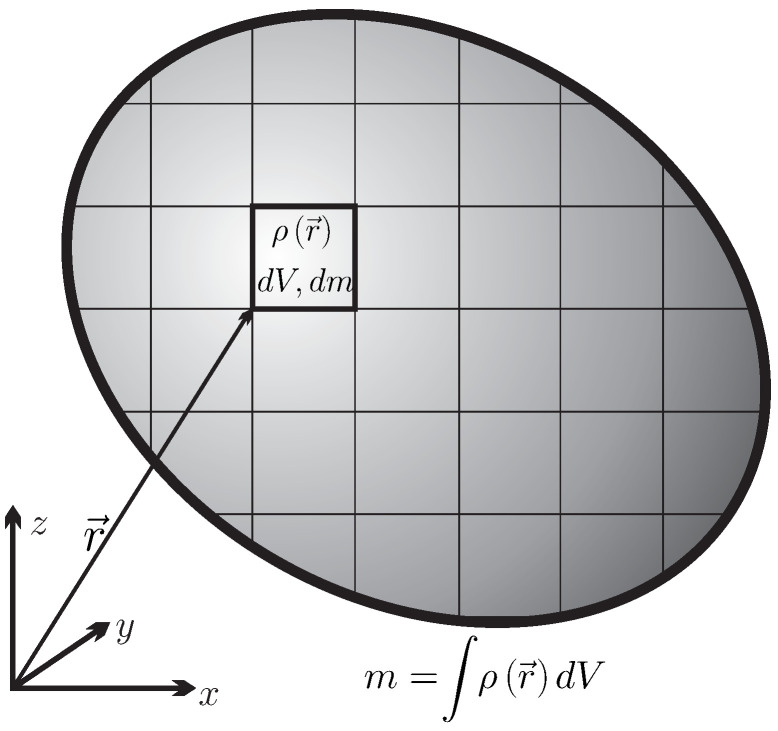
Inhomogeneous distribution of mass density $\rho \left(\vec{r}\right)$ in a system.

To get a good grip on properties that describe the system, we consider a system of volume *V* which is filled by a mass *m* of substance. To describe variation of properties in space, it is useful to divide the system into infinitesimal elements of size dV which contain the mass dm, as sketched in Figure 2.

The volume V=∫dV filled by the substance can, in principle, be measured by means of a ruler. The mass m=∫dm of the substance can be measured using a scale. The pressure *p* of the substance can be measured as the force required to keep a piston in place, divided by the surface area of the piston.

One distinguishes between extensive properties, which are related to the size of the system, and intensive properties, which are independent of the overall size of the system. Mass *m* and volume *V* are extensive quantities, for example, they double when the system is doubled; pressure *p* and temperature *T* (yet to be defined) are intensive properties, they remain unchanged when the system is doubled.

A particular class of intensive properties are the specific properties, which are defined as the ratio between an extensive property and the corresponding mass. In inhomogeneous states intensive and specific properties vary locally, that is they have different values in different volume elements dV. The *local* specific properties are defined through the values of the extensive property dΦ and the mass dm in the volume element,

(2) $$\phi =\frac{d\Phi }{dm}\;.$$

For example, the local specific volume *v*, and the local mass density ρ, are defined as

(3) $$v=\frac{1}{\rho }=\frac{dV}{dm}\;.$$

The values of the extensive properties for the full system are determined by integration of the specific properties over the mass or volume elements,

(4) Φ=∫dΦ=∫ϕdm=∫ρϕdV.

As an example, Figure 2 shows the inhomogeneous distribution of mass density ρ in a system (i.e., ϕ=1). Note that due to inhomogeneity, the density is a function of location $\vec{r}=\left\{x,y,z\right\}$ of the element dV, hence $\rho =\rho \left(\vec{r}\right)$.

For homogeneous states, the integrands can be taken out of the integrals, and we find simple relations such as

(5) $$v=\frac{1}{\rho }=\frac{V}{m}\;\;\;,\;\;\;\phi =\frac{\Phi }{m}\;.$$

## 6. Micro and Macro

A macroscopic amount of matter filling the volume *V*, say a steel rod or a gas in a box, consists of an extremely large number—to the order of 10${}^{23}$—of atoms or molecules. These are in constant interaction which each other and exchange energy and momentum, for example, a gas particle in air at standard conditions undergoes about 10${}^{9}$ collisions per second.

From the viewpoint of mechanics, one would have to describe each particle by its own (quantum mechanical) equation of motion, in which the interactions with all other particles would have to be taken into account. Obviously, due to the huge number of particles, this is not feasible. Fortunately, the constant interaction between particles leads to a collective behavior of the matter already in very small volume elements dV, in which the state of the matter can be described by few macroscopic properties like pressure, mass density, temperature and others. This allows us to describe the matter not as an assembly of atoms, but as a continuum where the state in each volume element dV is described by these few macroscopic properties.

Note that the underlying assumption is that the volume element contains a sufficiently large number of particles, which interact with high frequency. Indeed, the continuum hypothesis breaks down under certain circumstances, in particular for highly rarefied gases [17]. In all of what follows, however, we shall only consider systems in which the assumption is well justified. Appendix D provides a short discussion of kinetic gas theory, where macroscopic thermodynamics arises in the limit of high collision frequency between particles (equivalent to small mean free path).

## 7. Processes and Equilibrium States

A *process* is any change in one or more properties occurring within a system. The system depicted in Figure 1 can be manipulated by moving the piston or propeller, and by changing the temperature of the system boundary (heating/cooling coil). Any manipulation changes the state of the system locally and globally—a process occurs.

After all manipulation stops, the states, that is, the values of the local intensive properties in the volume elements, will keep changing for a while—that is the process continues—until a stable final state is assumed. This stable final state is called the *equilibrium state*. The system will remain in the equilibrium state until a new manipulation commences.

Simple examples from daily life are:

(a) A cup of coffee is stirred with a spoon. After the spoon is removed, the coffee will keep moving for a while until it comes to rest. It will stay at rest indefinitely, unless stirring is recommenced or the cup is moved.
(b) Milk is poured into coffee. Initially, there are light-brown regions of large milk content and dark-brown regions of low milk content. After a while, however, coffee and milk are well-mixed, at mid-brown color, and remain in that state. Stirring speeds the process up, but the mixing occurs also when no stirring takes place. Personally, I drink standard dip-coffee into which I pour milk: I have not used a spoon for mixing both in years.
(c) A spoon used to stir hot coffee becomes hot at the end immersed in the coffee. A while after it is removed from the cup, it will have assumed a homogeneous temperature.
(d) Oil mixed with vinegar by stirring will separate after a while, with oil on top of the vinegar.

In short, observation of daily processes, and experiments in the laboratory, show that a system that is left to itself for a sufficiently long time will approach a stable equilibrium state, and will remain in this state as long as the system is not subjected to further manipulation. This experience is the content of **Observation 2**. Example (d) shows that not all equilibrium states are homogeneous; however, temperature will always be homogeneous in equilibrium, which is laid down as **Observation 3**.

The details of the equilibrium state depend on the constraints on the system, in particular material, size, mass, and energy; this will become clear further below (Section 39).

The time required for reaching the equilibrium state, and other details of the process taking place, depend on the initial deviation from the equilibrium state, the material, and the geometry. Some systems may remain for rather long times in metastable states—these will not be further discussed.

Physical constraints between different parts of a system can lead to different equilibrium states within the parts. For instance, a container can be divided by a rigid wall, with different materials at both sides. Due to the physical division, the materials in the compartments might well be at different pressures, and different temperatures, and they will not mix. However, if the wall is diathermal, that is, it allows heat transfer, then the temperature will equilibrate between the compartments. If the wall is allowed to move, it will do so, until the pressures in both parts are equal. If the wall is removed, depending on their miscibility the materials might mix, see examples (b) and (d).

Unless otherwise stated, the systems discussed in the following are free from internal constraints.

## 8. Reversible and Irreversible Processes

When one starts to manipulate a system that is initially in equilibrium, the equilibrium state is disturbed, and a new process occurs.

All real-life applications of thermodynamics involve some degree of nonequilibrium. For the discussion of thermodynamics it is customary, and useful, to consider idealized processes, for which the manipulation happens sufficiently slow. In this case, the system has sufficient time to adapt so that it is in an equilibrium state at *any* time. Slow processes that lead the system through a series of equilibrium states are called *quasi-static*, or *quasi-equilibrium*, or *reversible*, processes.

If the manipulation that causes a quasi-static process stops, the system is already in an equilibrium state, and no further change will be observed.

Equilibrium states are simple, quite often they are homogenous states, or can be approximated as homogeneous states (see Section 40). The state of the system is fully described by few extensive properties, such as mass, volume, energy, and the corresponding pressure and temperature.

When the manipulation is fast, so that the system has no time to reach a new equilibrium state, it will be in nonequilibrium states. If the manipulation that causes a nonequilibrium process stops, the system will undergo further changes until it has reached its equilibrium state. The equilibration process takes place while no manipulation occurs, that is, the system is left to itself. Thus, the equilibration is an uncontrolled process.

Nonequilibrium processes typically involve inhomogeneous states, hence their proper description requires values of the properties at all locations $\vec{r}$ (i.e., in all volume elements dV) of the system. Accordingly, the detailed description of nonequilibrium processes is more complex than the description of quasi-static processes. This is the topic of theories of nonequilibrium thermodynamics, where the processes are described through partial differential equations, see Appendix C. For instance, the approach of Linear Irreversible Thermodynamics yields the Navier-Stokes and Fourier laws that are routinely used in fluid dynamics and heat transfer. Apart from giving the desired spatially resolved description of the process, these equations are also useful in examining under which circumstances a process can be approximated as quasi-static. For the moment, we state that a process must be sufficiently slow for this to be the case.

The approach to equilibrium introduces a timeline for processes—As time progresses, an isolated system, that is, a system that is not further manipulated in any way, so that heat and work vanish, will talways approach, and finally reach, its unique equilibrium state. The opposite will not be observed, that is an isolated system will never be seen spontaneously leaving its equilibrium state when no manipulation occurs.

**Figure 3:**
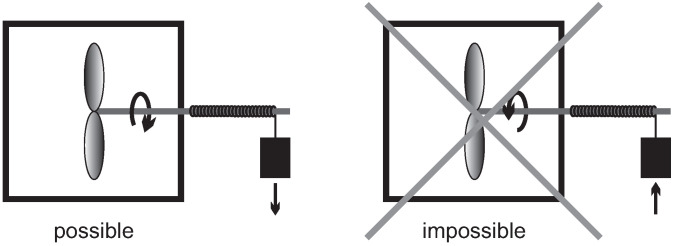
A possible and an impossible process.

Indeed, we immediately detect whether a movie of a nonequilibrium process is played forward or backwards: well mixed milk coffee will not separate suddenly into milk and coffee; a spoon of constant temperature will not suddenly become hot at one end, and cold at the other; a propeller immersed in a fluid at rest will not suddenly start to move and lift a weight (Figure 3); oil on top of water will not suddenly mix with the water; and so forth. We shall call processes with a time-line *irreversible*.

Only for quasi-static processes, where the system is always in equilibrium states, we cannot distinguish whether a movie is played forwards or backwards. This is why these processes are also called *reversible*. Since equilibration requires time, quasi-static, or reversible, processes typically are slow processes, so that the system always has sufficient time to adapt to an imposed change.

To be clear, we define quasi-static processes as reversible. One could consider irreversible slow processes, such as the compression of a gas with a piston subject to friction. For the gas itself, the process would be reversible, but for the system of gas *and* piston, the process would be irreversible.

## 9. Temperature and the 0th Law

By touching objects we can distinguish between hot and cold, and we say that hotter states have a higher temperature. Objective measurement of temperature requires (a) a proper definition, and (b) a proper device for measurement—a thermometer.

**Figure 4:**
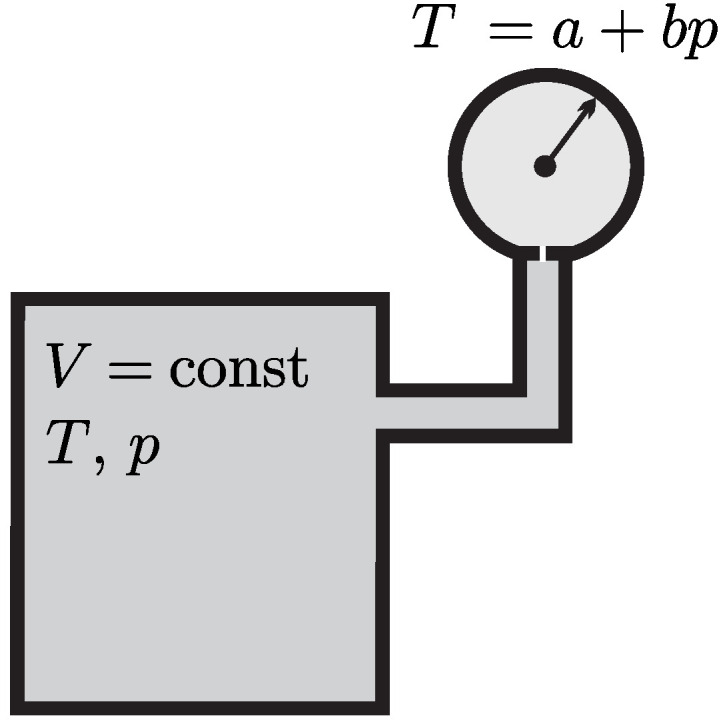
In a gas thermometer, temperature *T* is determined through measurement of pressure *p*.

Experience shows that physical states of systems change with temperature. For instance, the gas thermometer in Figure 4 contains a certain amount of gas enclosed in a container at fixed volume *V*. Increase of its temperature *T* by heating leads to a measurable change in the gas pressure *p*. Note that pressure is a mechanical property, which is measured as force per area. An arbitrary temperature scale can be defined, for example, as T=a+bp with arbitrary constants *a* and *b*.

**Figure 5:**
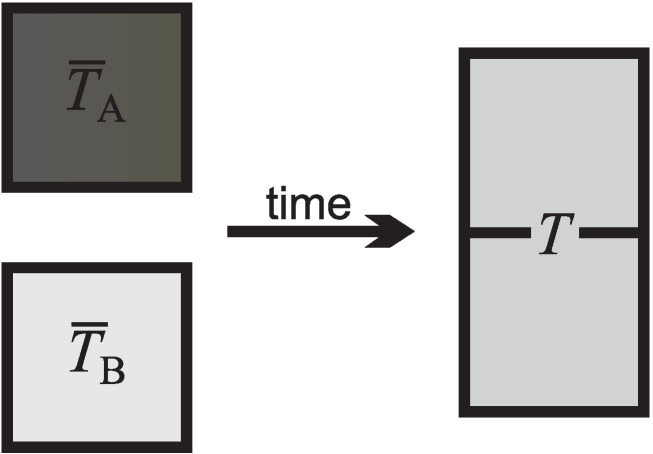
Two bodies of different temperatures ${\bar{T}}_{A}$, ${\bar{T}}_{B}$ assume a common temperature *T* a while after they are brought into thermal contact.

To study temperature, we consider two systems, initially in their respective equilibrium, both not subject to any work interaction, that is, no piston or propeller motion in Figure 1, which are manipulated by bringing them into physical contact, such that energy can pass between the systems (thermal contact), see Figure 5. Then, the new system that is comprised of the two initial systems will exhibit a process towards its equilibrium state. Consider first equilibration of a body **A** with the gas thermometer, so that the compound system of body and thermometer has the initial temperature ${\bar{T}}_{A}$, which can be read of the thermometer. Next, consider the equilibration of a body **B** with the gas thermometer, so that the compound system of body and thermometer has the initial temperature ${\bar{T}}_{B}$, as shown on the thermometer.

Now, we bring the two bodies and the thermometer into thermal contact, and let them equilibrate. It is observed that both systems change their temperature such the hotter system becomes colder, and vice versa. Independent of whether the thermometer is in thermal contact only with system **A** or in thermal contact with system **B**, it shows the same temperature. Hence, the equilibrium state is characterized by a common temperature of both systems. Since no work interaction took place, one speaks of the *thermal equilibrium* state.

Expressed more formally, we conclude that if body **C** (the thermometer in the above) is in thermal equilibrium with body **A** and in thermal equilibrium with body **B**, than also bodies **A** and **B** will be in thermal equilibrium. All three bodies will have the same temperature. The extension to an arbitrary number of bodies is straightforward, and since any system under consideration can be thought of as a compund of smaller subsystems, we can conclude that a system in thermal equilibrium has a homogeneous temperature.

The observation outlined above defines temperature, hence its important enough to be laid out as a law (**Observation 3**)

**The 0th Law of Thermodynamics**

*In a stable equilibrium state, the temperature of a thermally unrestricted system is uniform. Or, two bodies in thermal equilibrium have the same temperature.*

The 0th law introduces temperature as a measurable quantity. Indeed, to measure the temperature of a body, all we have to do is to bring a calibrated thermometer into contact with the body and wait until the equilibrium state of the system (body and thermometer) is reached. When the size of the thermometer is sufficiently small compared to the size of the body, the final temperature of body *and* thermometer will be (almost) equal to the initial temperature of the body.

## 10. Ideal Gas Temperature Scale

For proper agreement and reproducibility of temperature measurement, it is helpful to agree on a temperature scale.

Any gas at sufficiently low pressures and large enough temperatures, behaves as an ideal gas. From experiments one observes that for an ideal gas confined to a fixed volume the pressure increases with temperature. The temperature scale is *defined* such that the relation between pressure and temperature is linear, that is

(6) T=a+bp

The Celsius scale was originally defined based on the boiling and freezing points of water at p=1atm to define the temperatures of 100 ${}^{{}^{\circ}}C$ and 0 ${}^{{}^{\circ}}C$. For the Celsius scale one finds a=−273.15 ${}^{{}^{\circ}}C$ independent of the ideal gas used. The constant *b* depends on the volume, mass and type of the gas in the thermometer.

By shifting the temperature scale by *a*, one can define an alternative scale, the *ideal gas temperature scale*, as

(7) $$T\left(K\right)=bp\;.$$

The ideal gas scale has the unit Kelvin [K] and is related to the Celsius scale as

(8) $$T\left(K\right)=T\left({}^{{}^{\circ}}C\right)\frac{K}{{}^{{}^{\circ}}C}+273.15K\;.$$

It will be shown later that this scale coincides with the thermodynamic temperature scale that follows from the 2nd law (Section 27).

## 11. Thermal Equation of State

Careful measurements on simple substances show that specific volume *v* (or density ρ=1/v), pressure *p* and temperature *T* cannot be controlled independently. Indeed, in equilibrium states they are linked through a relation of the form $p=p\left(v,T\right)$, or $p=p\left(\rho ,T\right)$, known as the *thermal equation of state*. For most substances, this relation cannot be easily expressed as an actual equation, but is laid down in property tables.

The thermal equation of state relates measurable properties. It suffices to know the values of two properties to determine the values of others. This will still be the case when we add energy and entropy in equilibrium states to the list of thermodynamic properties, which can be determined through measurement of any two of the measurable properties, that is, $\left(p,T\right)$ or $\left(v,T\right)$ or $\left(p,v\right)$.

To summarize: If we assume local thermal equilibrium, the complete knowledge of the macroscopic state of a system requires the values of two intensive properties in each location (i.e., in each infinitesimal volume element), and the local velocity. The state of a system in global equilibrium, where properties are homogeneous, is described by just two intensive properties (plus the size of the system, that is either total volume, or total mass). In comparison, full knowledge of the microscopic state would require the knowledge of location and velocity of each particle.

The ideal gas is one of the simplest substances to study, since it has simple property relations. Careful measurements have shown that for an ideal gas pressure *p*, total volume *V*, temperature *T* (in K), and mass *m* are related by an explicit thermal equation of state, the *ideal gas law*

(9) pV=mRT.

Here, *R* is the *gas constant* that depends on the type of the gas. With this, the constant in (7) is $b=V/\left(mR\right)=v/R$.

Alternative forms of the ideal gas equation result from introducing the specific volume v=V/m or the mass density ρ=1/v so that

(10) pv=RT,p=ρRT.

## 12. The 1st Law of Thermodynamics

It is our daily experience that heat can be converted to work, and that work can be converted to heat. A propeller mounted over a burning candle will spin when the heated air rises due to buoyancy: heat is converted to work. Rubbing our hands makes them warmer: work is converted to heat. Humankind has a long and rich history of making use of both conversions.

While the heat-to-work and work-to-heat conversions are readily observable in simple and more complex processes, the governing law is not at all obvious from simple observation. It required groundbreaking thinking and careful experiments to unveil the *Law of Conservation of Energy*. Due to its importance in thermodynamics, it is also known as the *1st Law of Thermodynamics*, which expressed in words, reads:

**1st Law of Thermodynamics**

*Energy cannot be produced nor destroyed, it can only be transferred, or converted from one form to another. In short, energy is conserved.*

It took quite some time to formulate the 1st law in this simple form, the credit for finding and formulating it goes to Robert Meyer (1814–1878), James Prescott Joule (1818–1889), and Hermann Helmholtz (1821–1894). Through careful measurements and analysis, they recognized that thermal energy, mechanical energy, and electrical energy can be transformed into each other, which implies that energy can be transferred by doing work, as in mechanics, and by heat transfer.

The 1st law is generally valid, no violation was ever observed. As knowledge of physics has developed, other forms of energy had to be included, such as radiative energy, nuclear energy, or the mass-energy equivalence of the theory of relativity, but there is no doubt today that energy is conserved under all circumstances.

We formulate the 1st law for the simple closed system of Figure 1, where all three possibilities to manipulate the system from the outside are indicated. For this system, the conservation law for energy reads

(11) $$\frac{dE}{dt}=\dot{Q}-\dot{W}\;,$$

where *E* is the total energy of the system, $\dot{Q}$ is the total heat transfer rate in or out of the system, and $\dot{W}\;=\;{\dot{W}}_{piston}+{\dot{W}}_{propeller}$ is the total power—the work per unit time—exchanged with the surroundings. Energy is an extensive property, hence also heat and work scale with the size of the system. For instance, doubling the system size, doubles the energy, and requires twice the work and heat to observe the same changes of the system.

This equation states that the change of the system’s energy in time (dE/dt) is equal to the energy transferred by heat and work per unit time ($\dot{Q}-\dot{W}$). The sign convention used is such that heat transferred *into* the system is positive, and work done *by* the system is positive.

## 13. Energy

There are many forms of energy that must be accounted for. For the context of the present discussion, the total energy *E* of the system is the sum of its kinetic energy $E_{kin}$, potential energy $E_{pot}$, and internal—or thermal—energy *U*,

(12) $$E=U+E_{kin}+E_{pot}\;.$$

The kinetic energy is well-known from mechanics. For a homogeneous system of mass *m* and barycentric velocity V, kinetic energy is given by

(13) $$E_{kin}=\frac{m}{2}V^{2}\;.$$

For inhomogeneous states, where each mass element has its own velocity, the total kinetic energy of the system is obtained by integration of the specific kinetic energy $e_{kin}$ over all mass elements dm=ρdV;

(14) $$e_{kin}=\frac{1}{2}V^{2}\;\;\;\mathrm{and}\;\;\;E_{kin}=\int \rho e_{kin}dV=\int \frac{\rho }{2}V^{2}dV\;.$$

Also the potential energy in the gravitational field is well-known from mechanics. For a homogeneous system of mass *m*, potential energy is given by

(15) $$E_{pot}=mg_{n}\bar{z}\;,$$

where $\bar{z}$ is the elevation of the system’s center of mass over a reference height, and $g_{n}=9.81\frac{m}{s^{2}}$ is the gravitational acceleration on Earth. For inhomogeneous states the total potential energy of the system is obtained by integration of the specific potential energy $e_{pot}$ over all mass elements dm=ρdV; we have

(16) $$e_{pot}=g_{n}z\;\;\;\mathrm{and}\;\;\;E_{pot}=\int \rho e_{pot}dV=\int \rho g_{n}zdV\;.$$

Even if a macroscopic element of matter is at rest, its atoms move about (in a gas or liquid) or vibrate (in a solid) fast, so that each atom has microscopic kinetic energy. The atoms are subject to interatomic forces, which contribute microscopic potential energies. Moreover, energy is associated with the atoms’ internal quantum states. Since the microscopic energies cannot be observed macroscopically, one speaks of the *internal energy*, or *thermal energy*, of the material, denoted as *U*.

For inhomogeneous states the total internal energy of the system is obtained by integration of the specific internal energy *u* over all mass elements dm=ρdV. For homogeneous and inhomogeneous systems we have

(17) U=muandU=∫ρudV.

## 14. Caloric Equation of State

Internal energy cannot be measured directly. The *caloric equation of state* relates the specific internal energy *u* to measurable quantities in equilibrium states, it is of the form $u=u\left(T,v\right)$, or $u=u\left(T,p\right)$. Recall that pressure, volume and temperature are related by the thermal equation of state, $p\left(v,T\right)$; therefore it suffices to know two properties in order to determine the others.

We note that internal energy summarizes all microscopic contributions to energy. Hence, a system, or a volume element within a system, will always have internal energy *u*, independent of whether the system is in (local) equilibrium states or in arbitrarily strong nonequilibrium states. Only in the former, however, does the caloric equation of state provide a link between energy and measurable properties.

The caloric equation of state must be determined by careful measurements, where the response of the system to heat or work supply is evaluated by means of the first law. For most materials the results cannot be easily expressed as equations, and are tabulated in property tables.

We consider a closed system heated slowly at constant volume (*isochoric* process), with homogeneous temperature *T* at all times. Then, the first law (27) reduces to (recall that $U=mu\left(T,v\right)$ and m=const.)

(18) $$m{\left(\frac{\partial u}{\partial T}\right)}_{v}\frac{dT}{dt}=\dot{Q}\;.$$

Here, we use the standard notation of thermodynamics, where ${\left(\frac{\partial u}{\partial T}\right)}_{v}=\frac{\partial u\left(T,v\right)}{\partial T}$ denotes the partial derivative of internal energy with temperature at constant specific volume v=V/m. This derivative is known as the *specific heat* (or *specific heat capacity*)* at constant volume*,

(19) $$c_{v}={\left(\frac{\partial u}{\partial T}\right)}_{v}\;.$$

As defined here, based on SI units, the specific heat $c_{v}$ is the amount of heat required to increase the temperature of 1kg of substance by 1K at constant volume. It can be measured by controlled heating of a fixed amount of substance in a fixed volume system, and measurement of the ensuing temperature difference; its SI unit is $\left[\frac{\mathrm{kJ}}{\mathrm{kg}K}\right]$.

In general, internal energy $u\left(T,v\right)$ is a function of a function of temperature and specific volume. For incompressible liquids and solids the specific volume is constant, v=const, and the internal energy is a function of temperature alone, $u\left(T\right)$. Interestingly, also for ideal gases the internal energy turns out to be a function of temperature alone, both experimentally and from theoretical considerations. For these materials the specific heat at constat volume depends only on temperature, $c_{v}\left(T\right)=\left(\frac{du}{dt}\right)$ and its integration gives the caloric equation of state as

(20) $$u\left(T\right)=\int_{T_{0}}^{T}c_{v}\left(T^{\prime }\right)dT^{\prime }+u_{0}\;.$$

Only energy differences can be measured, where the first law is used to evaluate careful experiments. The choice of the energy constant $u_{0}=u\left(T_{0}\right)$ fixes the energy scale. The actual value of this constant is relevant for the discussion of chemical reactions [8]. Note that proper mathematical notation requires to distinguish between the actual temperature *T* of the system, and the integration variable $T^{\prime }$.

For materials in which the specific heat varies only slightly with temperature in the interval of interest, the specific heat can be approximated by a suitable constant average $c_{v}$, so that the caloric equation of state assumes the particularly simple linear form

(21) $$u\left(T\right)=c_{v}\left(T-T_{0}\right)+u_{0}\;.$$

## 15. Work and Power

Work, denoted by *W*, is the product of a force and the displacement of its point of application. Power, denoted by $\dot{W}$, is work done per unit time, that is the force times the velocity of its point of application. The total work for a process is the time integral of power over the duration $\Delta t=t_{2}-t_{1}$ of the process,

(22) $$W=\int_{t_{1}}^{t_{2}}\dot{W}dt\;.$$

For the closed system depicted in Figure 1 there are two contributions to work: *moving boundary work*, due to the motion of the piston, and *rotating shaft work,* which moves the propeller. Other forms of work, for example, spring work or electrical work could be added as well.

Work and power can be positive or negative. We follow the sign convention that work done *by* the system is positive and work done *to* the system is negative.

For systems with homogeneous pressure *p*, which might change with time as a process occurs (e.g., the piston moves), one finds the following expressions for moving boundary work with finite and infinitesimal displacement, and for power,

(23) $$W_{12}=\int_{1}^{2}pdV\;\;\;,\;\;\;\delta W=pdV\;\;\;,\;\;\;\dot{W}=p\frac{dV}{dt}\;.$$

Moving boundary work depends on the process path, so that the work exchanged for an infinitesimal process step, $\delta W=pdV=\dot{W}dt$, is not an exact differential (see next section). Closed equilibrium systems are characterized by a single homogeneous pressure *p*, a single homogeneous temperature *T*, and the volume *V*. In quasi-static (or reversible) processes, the system passes through a series of equilibrium states which can be indicated in suitable diagrams, for example, the p-V-diagram.

In a closed system the propeller stirs the working fluid and creates inhomogeneous states. The power is related to the torque T and the revolutionary speed $\dot{n}$ (revolutions per unit time) as $\dot{W}=2\pi \dot{n}T$. Fluid friction transmits fluid motion (i.e., momentum and kinetic energy) from the fluid close to the propeller to the fluid further away. Due to the inherent inhomogeneity, stirring of a fluid in a closed system cannot be a quasi-static process, and is *always* irreversible.

In general, there might be several work interactions ${\dot{W}}_{j}$ of the system, then the total work for the system is the sum over all contributions; for example, for power

(24) $$\dot{W}=\sum_{j}{\dot{W}}_{j}\;.$$

For reversible processes with additional work contributions, one has $\dot{W}=\sum_{j}x_{j}\frac{dY_{j}}{dt}\;$, where $\left\{x_{j},Y_{j}\right\}$ are pairs of conjugate work variables, such as {p,V}.

Finally, we know from the science of mechanics that by using gears and levers, one can transfer energy as work from slow moving to fast moving systems and vice versa, and one can transmit work from high pressure to low pressure systems and vice versa. However, due to friction within the mechanical system used for transmission of work, some of the work may be lost. This experience is formulated in **Observation 4**.

## 16. Exact and Inexact Differentials

Above we have seen that work depends on the process path. In the language of mathematics this implies that the work for an infinitesimal step is not an exact differential, and that is why a Greek delta (δ) is used to denote the work for an infinitesimal change as δW. As will be seen in the next section, heat is path dependent as well.

State properties like pressure, temperature, volume and energy describe the momentary state of the system, or, for inhomogeneous states, the momentary state in the local volume element. State properties have exact differentials for which we write, for example, dE and dV. The energy change $E_{2}-E_{1}=\int_{1}^{2}dE$ and the volume change $V_{2}-V_{1}=\int_{1}^{2}dV$ are independent of the path connecting the states.

It is important to remember that work and heat, as path functions, do not describe states, but the processes that leads to changes of the state. Hence, for a process connecting two states 1,2 we write $W_{12}=\int_{1}^{2}\delta W$, $Q_{12}=\int_{1}^{2}\delta Q$, where $W_{12}$ and $Q_{12}$ are the energy transferred across the system boundaries by heat or work.

A state is characterized by state properties (pressure, temperature, etc.), it does not possess work or heat.

Quasi-static (reversible) processes go through well defined equilibrium states, so that the whole process path can be indicated in diagrams, for example, the p-V-diagram.

**Figure 6:**
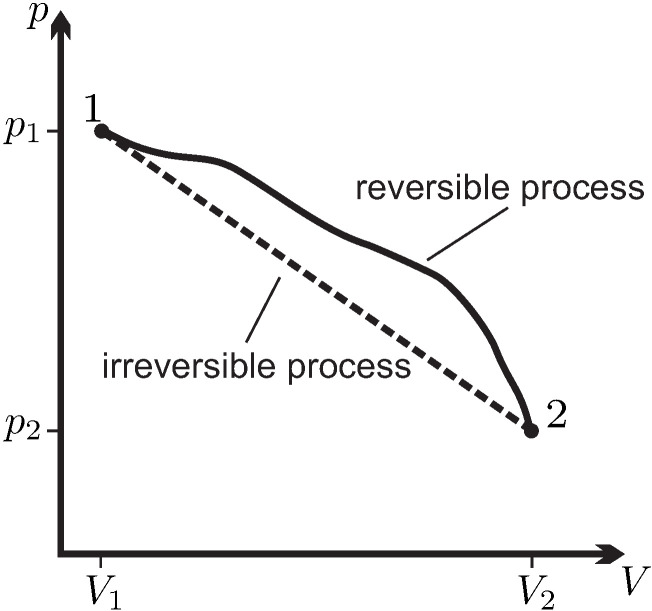
A reversible (quasi-static) and an irreversible (nonequilibrium) process between the equilibrium states 1 and 2.

Nonequilibrium (irreversible) processes, for which typically the states are different in all volume elements, cannot be drawn into diagrams. Often irreversible processes connect homogeneous equilibrium states which can be indicated in the diagram. It is recommended to use dashed lines to indicate nonequilibrium processes that connect equilibrium states. As an example, Figure 6 shows a p-V-diagram of two processes, one reversible, one irreversible, between the same equilibrium states 1 and 2. We emphasize that the dashed line does not refer to actual states of the system. The corresponding work for the nonequilibrium process cannot be indicated as the area below the curve, since its computation requires the knowledge of the—inhomogeneous!—pressures at the piston surface at all times during the process.

## 17. Heat Transfer

*Heat* is the transfer of energy due to differences in temperature. Experience shows that for systems in thermal contact the direction of heat transfer is restricted, such that heat will always go from hot to cold by itself, but not vice versa. This experience is formulated in **Observation 5**.

This restriction of direction is an important difference to energy transfer by work between systems in mechanical contact, which is not restricted.

Since heat flows only in response to a temperature difference, a quasi-static (reversible) heat transfer process can only be realized in the limit of infinitesimal temperature differences between the system and the system boundary, and for infinitesimal temperature gradients within the system.

We use the following notation: $\dot{Q}$ denotes the heat transfer rate, that is the amount of energy transferred as heat per unit time. Heat depends on the process path, so that the heat exchanged for an infinitesimal process step, $\delta Q=\dot{Q}dt$, is not an exact differential. The total heat transfer for a process between states 1 and 2 is

(25) $$Q_{12}=\int_{1}^{2}\delta Q=\int_{t_{1}}^{t_{2}}\dot{Q}dt\;.$$

By the convention used, heat transferred into the system is positive, heat transferred out of the system is negative.

A process in which no heat transfer takes place, $\dot{Q}=0$, is called *adiabatic process.*

In general, there might be several heat interactions ${\dot{Q}}_{k}$ of the system, then the total heat for the system is the sum over all contributions; for example, for the heating rate

(26) $$\dot{Q}=\sum_{k}{\dot{Q}}_{k}\;.$$

For the discussion of the 2nd law we will consider the ${\dot{Q}}_{k}$ as heat crossing the system boundary at locations where the boundary has the temperature $T_{k}$.

## 18. 1st Law for Reversible Processes

The form (11) of the first law is valid for *all* closed systems. When only reversible processes occur within the system, so that the system is in equilibrium states at any time, the equation can be simplified as follows: From our discussion of equilibrium states we know that for reversible processes the system will be homogeneous and that all changes must be very slow, which implies very small velocities relative to the center of mass of the system. Therefore, kinetic energy, which is velocity squared, can be ignored, $E_{kin}=0$. Stirring, which transfers energy by moving the fluid and friction, is irreversible, hence in a reversible process only moving boundary work can be transferred, where piston friction is absent. As long as the system location does not change, the potential energy does not change, and we can set $E_{pot}=0$.

With all this, for reversible (quasi-static) processes the 1st law of thermodynamics reduces to

(27) $$\frac{dU}{dt}=\dot{Q}-p\frac{dV}{dt}\;\;\;\;\mathrm{or}\;\;\;\;U_{2}-U_{1}=Q_{12}-\int_{1}^{2}pdV\;,$$

where the second form results from integration over the process duration.

## 19. Entropy and the Trend to Equilibrium

The original derivation of the 2nd law is due to Sadi Carnot (1796–1832) and Rudolf Clausius (1822–1888), where discussions of thermodynamic engines combined with **Observation 5** were used to deduce the 2nd law [1]. Even today, many textbooks present variants of their work [2,3,4,5]. As discussed in the introduction, we aim at introducing entropy without the use of heat engines, only using the 5 observations.

We briefly summarize our earlier statements on processes in closed systems: a closed system can be manipulated by exchange of work and heat with its surroundings only. In nonequilibrium—that is, irreversible—processes, when all manipulation stops, the system will undergo further changes until it reaches a final equilibrium state. This equilibrium state is stable, that is the system will not leave the equilibrium state spontaneously. It requires new action—exchange of work or heat with the surroundings—to change the state of the system. This paragraph is summarized in **Observations 1–3.**

The following nonequilibrium processes are well-known from experience, and will be used in the considerations below:

(a) Work can be transferred without restriction, by means of gears and levers. However, in transfer some work might be lost to friction (**Observation 4**).
(b) Heat goes from hot to cold. When two bodies at different temperatures are brought into thermal contact, heat will flow from the hotter to the colder body until both reach their common equilibrium temperature (**Observation 5**).

The process from an initial nonequilibrium state to the final equilibrium state requires some time. However, if the actions on the system (only work and heat!) are sufficiently slow, the system has enough time to adapt and will be in equilibrium states at all times. We speak of quasi-static—or, reversible—processes. When the slow manipulation is stopped at any time, no further changes occur.

If a system is not manipulated, that is there is neither heat or work exchange between the systems and its surroundings, we speak of an *isolated system*. The behavior of isolated systems described above—a change occurs until a stable state is reached—can be described mathematically by an inequality. The final stable state must be a maximum (alternatively, a minimum) of a suitable property describing the system. For a meaningful description of systems of arbitrary size, the new property should scale with system size, that is it must be extensive.

We call this new extensive property *entropy*, denoted *S*, and write an inequality for the isolated system,

(28) $$\frac{dS}{dt}={\dot{S}}_{gen}\geq 0\;.$$

${\dot{S}}_{gen}$ is called the *entropy generation rate*. The entropy generation rate is positive in nonequilibrium (${\dot{S}}_{gen}>0$), and vanishes in equilibrium (${\dot{S}}_{gen}=0$). The new Equation (28) states that in an isolated system the entropy will grow in time ($\frac{dS}{dt}>0$) until the stable equilibrium state is reached ($\frac{dS}{dt}\;=\;0$). Non-zero entropy generation, ${\dot{S}}_{gen}>0$, describes the irreversible process towards equilibrium, for example, through internal heat transfer and friction. There is no entropy generation in equilibrium, where entropy is constant. Since entropy only grows before the isolated system reaches its equilibrium state, the latter is a maximum of entropy.

While this equation describes the observed behavior in principle, it does not give a hint at what the newly introduced quantities *S* and ${\dot{S}}_{gen}$—entropy and entropy generation rate—are, or how they can be determined. Hence, an important part of the following discussion concerns the relation of entropy to measurable quantities, such as temperature, pressure, and specific volume. Moreover, it will be seen that entropy generation rate describes the irreversibility in, for example, heat transfer across finite temperature difference, or frictional flow.

The above postulation of an inequality is based on phenomenological arguments. The discussion of irreversible processes has shown that over time all isolated systems will evolve to a unique equilibrium state. The first law alone does not suffice to describe this behavior. Nonequilibrium processes aim to reach equilibrium, and the inequality is required to describe the clear direction in time.

As introduced here, entropy and the above rate equation describe irreversible processes, where initial nonequilibrium states evolve towards equilibrium. Not only is there no reason to restrict entropy to equilibrium, but rather, in this philosophy, it is essential to define entropy as a nonequilibrium property.

In the next sections we will extend the second law to non-isolated systems, identify entropy as a measurable property—at least in equilibrium states—and discuss entropy generation in irreversible processes.

## 20. Entropy Transfer

In non-isolated systems, which are manipulated by exchange of heat and work with their surroundings, we expect an exchange of entropy with the surroundings which must be added to the entropy inequality. We write

(29) $$\frac{dS}{dt}=\dot{\Gamma }+{\dot{S}}_{gen},\;\;\;\;\mathrm{with}\;{\dot{S}}_{gen}\geq 0\;,$$

where $\dot{\Gamma }$ is the *entropy transfer rate*. This equation states that the change of entropy in time (dS/dt) is due to transport of entropy over the system boundary ($\dot{\Gamma }$) and generation of entropy within the system boundaries (${\dot{S}}_{gen}$). This form of the second law is valid for all processes in closed systems. The entropy generation rate is positive (${\dot{S}}_{gen}>0$) for irreversible processes, and it vanishes (${\dot{S}}_{gen}=0$) in equilibrium and for reversible processes, where the system is in equilibrium states at all times.

All real technical processes are somewhat irreversible, since friction and heat transfer cannot be avoided. Reversible processes are idealizations that can be used to study the principal behavior of processes, and best performance limits.

We apply **Observation 1**: Since a closed system can only be manipulated through the exchange of heat and work with the surroundings, the transfer of any other property, including the transfer of entropy, must be related to heat and work, and must vanish when heat and work vanish. Therefore the entropy transfer $\dot{\Gamma }$ can only be of the form

(30) $$\dot{\Gamma }=\sum_{k}\beta_{k}{\dot{Q}}_{k}-\sum_{j}\gamma_{j}{\dot{W}}_{j}\;,$$

Recall that total heat and work transfer are the sum of many different contributions, $\dot{Q}=\sum_{k}{\dot{Q}}_{k}$ and $\dot{W}=\sum_{j}{\dot{W}}_{j}$. In the above formulation, the coefficients $\beta_{k}$ and $\gamma_{j}$ are used to distinguish heat and work transfer at different conditions at that part of the system boundary where the transfer (${\dot{Q}}_{k}$ or ${\dot{W}}_{j}$) takes place. Since work and heat scale with the size of the system, and entropy is extensive, the coefficients $\beta_{k}$ and $\gamma_{j}$ must be intensive, that is, independent of system size.

At this point, the coefficients $\beta_{k}$, $\gamma_{j}$ depend in an unknown manner on properties describing the state of the system and its interaction with the surroundings. While the relation between the entropy transfer rate $\dot{\Gamma }$ and the energy transfer rates ${\dot{Q}}_{k}$, ${\dot{W}}_{j}$ is not necessarily linear, the form (30) is chosen to clearly indicate that entropy transfer is zero when no energy is transferred, $\dot{\Gamma }=0$ if ${\dot{Q}}_{k}={\dot{W}}_{j}=0$ (isolated system).

With this expression for entropy transfer, the 2nd law assumes the form

(31) $$\frac{dS}{dt}+\sum_{j}\gamma_{j}{\dot{W}}_{j}-\sum_{k}\beta_{k}{\dot{Q}}_{k}={\dot{S}}_{gen}\geq 0\;,$$

This equation gives the mathematical formulation of the trend to equilibrium for a non-isolated closed system (exchange of heat and work, but not of mass). The next step is to identify entropy *S* and the coefficients $\beta_{k}$, $\gamma_{j}$ in the entropy transfer rate $\dot{\Gamma }$ in terms of quantities we can measure or control.

## 21. Direction of Heat Transfer

A temperature reservoir is defined as a large body in equilibrium whose temperature does not change when heat is removed or added (this requires that the reservoir’s thermal mass, $m_{R}c_{R}$, approaches infinity).

We consider heat transfer between two reservoirs of temperatures $T_{H}$ and $T_{L}$, where $T_{H}$ is the temperature of the hotter reservoir. The heat is transferred through a heat conductor (**HC**), which is the thermodynamic system to be evaluated. A pure steady state heat transfer problem is studied, where the conductor receives the heat flows ${\dot{Q}}_{H}$ and ${\dot{Q}}_{L}$, and exchanges no work with the surroundings, $\dot{W}=0$.

**Figure 7:**
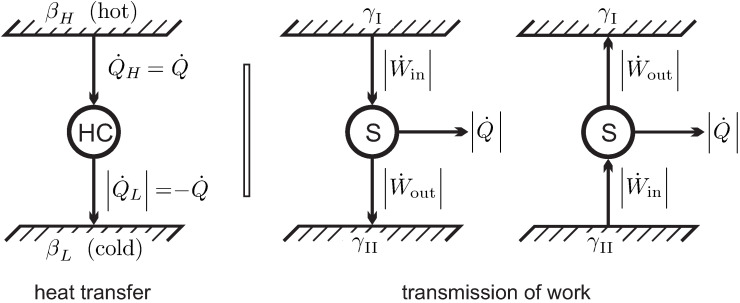
Heat transfer through a heat conductor HC (left) and transmission of work through a steady state system S (right).

The left part of Figure 7 shows a schematic of the heat transfer process. For steady state conditions no change over time is observed in the conductor, so that $\frac{dE}{dt}=\frac{dS}{dt}=0$. We emphasize that for this process the heat conductor will be in a nonequilibrium state, for example, it could be a solid heat conductor with an imposed temperature gradient, or, possibly, a gas in a state of natural convection in the gravitational field. To proceed with the argument, it is not necessary to quantify energy and entropy of the conductor, since both do not change in steady state processes.

For steady state, the first and second law (11, 31) applied to the heat conductor **HC** reduce to

(32) $$\begin{array}{ccc} {\dot{Q}}_{H} & = & -{\dot{Q}}_{L}=\dot{Q} \end{array}$$

(33) $$\begin{array}{ccc} -\beta_{H}{\dot{Q}}_{H}-\beta_{L}{\dot{Q}}_{L} & = & {\dot{S}}_{gen}\geq 0\;. \end{array}$$

Here, $\beta_{H}$ and $\beta_{L}$ are the values of β at the hot and cold sides of the conductor, respectively. Combining both we have

(34) $$\left(\beta_{L}-\beta_{H}\right)\dot{Q}={\dot{S}}_{gen}\geq 0\;.$$

We apply **Observation 5**: Since heat must go from hot to cold (from reservoir $T_{H}$ to reservoir $T_{L}$), the heat must be positive, $\dot{Q}=Q_{H}>0$, which requires $\left(\beta_{L}-\beta_{H}\right)>0$. Thus, the coefficient β must be smaller for the part of the system which is in contact with the hotter reservoir, $\beta_{H}<\beta_{L}$. This must be so irrespective of the values of *any* other properties at the system boundaries (L,H), that is, independent of the conductor material or its mass density, or any other material properties, and also for all possible values $\dot{Q}$ of the heat transferred. It follows, that $\beta_{L}$, $\beta_{H}$ must depend on temperature of the respective reservoir *only*.

Moreover, β must be a decreasing function of reservoir temperature alone, if temperature of the hotter reservoir is defined to be higher.

## 22. Work Transfer and Friction Loss

For the discussion of the coefficient γ we turn our attention to the transmission of work. The right part of Figure 7 shows two “work reservoirs” characterized by different values $\gamma_{I}$, $\gamma_{\mathrm{II}}$ between which work is transmitted by a steady state system S.

We apply **Observation 4**. The direction of work transfer is not restricted: by means of gears and levers work can be transmitted from low to high force and vice versa, and from low to high velocity and vice versa. Therefore, transmission might occur from I to II, and as well from II to I. Accordingly, there is no obvious interpretation of the coefficient γ. Indeed, we will soon be able to remove the coefficient γ from the discussion.

According to the second part of **Observation 4**, friction might occur in the transmission. Thus, in the transmission process we expect some work being lost to frictional heating, therefore $\left|{\dot{W}}_{out}\right|\leq \left|{\dot{W}}_{in}\right|$. In order to keep the transmission system at constant temperature, some heat must be removed to a reservoir (typically the outside environment). Work and heat for both cases are indicated in the figure, the arrows indicate the direction of transfer.

The first law for both transmission processes reads (steady state, $\frac{dE}{dt}=0$)

(35) $$0=-\left|\dot{Q}\right|-\left|{\dot{W}}_{out}\right|+\left|{\dot{W}}_{in}\right|\;,$$

where the signs account for the direction of the flows. Since work loss in transmission means $\left|{\dot{W}}_{out}\right|\leq \left|{\dot{W}}_{in}\right|$, this implies that heat must leave the system, $\dot{Q}=-\left|\dot{Q}\right|\leq 0$, as indicated in the figure.

Due to the different direction of work in the two processes considered, the second law (31) gives different conditions for both situations (steady state, $\frac{dS}{dt}=0$),

(36) $$-\gamma_{I}\left|{\dot{W}}_{in}\right|+\gamma_{\mathrm{II}}\left|{\dot{W}}_{out}\right|+\beta \left|\dot{Q}\right|\geq 0\;,\;\;\gamma_{I}\left|{\dot{W}}_{out}\right|-\gamma_{\mathrm{II}}\left|{\dot{W}}_{in}\right|+\beta \left|\dot{Q}\right|\geq 0\;,$$

where, as we have seen in the previous section, β is a measure for the temperature of the reservoir that accepts the heat. Elimination of the heat $\left|\dot{Q}\right|$ between first and second laws gives two inequalities,

(37) $$\left(\gamma_{\mathrm{II}}-\beta \right)\left|{\dot{W}}_{out}\right|-\left(\gamma_{I}-\beta \right)\left|{\dot{W}}_{in}\right|\geq 0\;,\;\left(\gamma_{I}-\beta \right)\left|{\dot{W}}_{out}\right|-\left(\gamma_{\mathrm{II}}-\beta \right)\left|{\dot{W}}_{in}\right|\geq 0,$$

or, after some reshuffling,

(38) $$\left(\beta -\gamma_{\mathrm{II}}\right)\frac{\left|{\dot{W}}_{out}\right|}{\left|{\dot{W}}_{in}\right|}\leq \left(\beta -\gamma_{I}\right)\;\;\;,\;\;\;\left(\beta -\gamma_{I}\right)\frac{\left|{\dot{W}}_{out}\right|}{\left|{\dot{W}}_{in}\right|}\leq \left(\beta -\gamma_{\mathrm{II}}\right)\;.$$

Combining the two equations (38) gives the two inequalities

(39) $$\left(\beta -\gamma_{I}\right){\left(\frac{\left|{\dot{W}}_{out}\right|}{\left|{\dot{W}}_{in}\right|}\right)}^{2}\leq \left(\beta -\gamma_{I}\right)\;\;\;,\;\;\;\left(\beta -\gamma_{\mathrm{II}}\right){\left(\frac{\left|{\dot{W}}_{out}\right|}{\left|{\dot{W}}_{in}\right|}\right)}^{2}\leq \left(\beta -\gamma_{\mathrm{II}}\right)\;.$$

From the these follows, since $0\leq \frac{\left|{\dot{W}}_{out}\right|}{\left|{\dot{W}}_{in}\right|}\leq 1$, that $\left(\beta -\gamma \right)\;$ must be non-negative, β−γ≥0.

Both inequalities (38) must hold for arbitrary transmission systems, that is for all $0\leq \frac{\left|{\dot{W}}_{out}\right|}{\left|{\dot{W}}_{in}\right|}\leq 1$, and all temperatures of the heat receiving reservoir, that is for all β. For a reversible transmission, where $\frac{\left|{\dot{W}}_{out}\right|}{\left|{\dot{W}}_{in}\right|}=1$, both inequalities (38) can only hold simultaneously if $\gamma_{I}=\gamma_{\mathrm{II}}$. Accordingly, $\gamma_{I}=\gamma_{\mathrm{II}}=\gamma$ must be a constant, and $\left(\beta -\gamma \right)\geq 0$ for all β.

With γ as a constant, the entropy balance (31) becomes

(40) $$\frac{dS}{dt}+\gamma \dot{W}-\sum \beta_{k}{\dot{Q}}_{k}={\dot{S}}_{gen}\geq 0\;,$$

where $\dot{W}=\sum_{j}{\dot{W}}_{j}$ is the net power for the system. The energy balance solved for power, $\dot{W}\;=\;\sum {\dot{Q}}_{k}\;-\;\frac{dE}{dt}$, allows us to eliminate work, so that the 2nd law becomes

(41) $$\frac{d\left(S-\gamma E\right)}{dt}-\sum \left(\beta_{k}-\gamma \right){\dot{Q}}_{k}={\dot{S}}_{gen}\geq 0\;.$$

## 23. Entropy and Thermodynamic Temperature

Without loss of generality, we can absorb the energy term γE into entropy, that is, we set

(42) $$\left(S-\gamma E\right)\to S\;;$$

this is equivalent to setting γ=0. Note that, since energy is conserved, any multiple of energy can be added to entropy without changing the principal features of the 2nd law; obviously, the most elegant formulation is the one where work does not appear.

Moreover, we have found that $\left(\beta -\gamma \right)$ is a non-negative monotonously decreasing function of temperature, and we *define* thermodynamic temperature as

(43) $$T=\frac{1}{\beta -\gamma }>0\;.$$

Note that non-negativity of inverse temperature implies that temperature itself is strictly positive.

With this, we have the 2nd law in the form

(44) $$\frac{dS}{dt}-\sum_{k}\frac{{\dot{Q}}_{k}}{T_{R,k}}={\dot{S}}_{gen}\geq 0\;.$$

The above line of arguments relied solely on the temperatures of the reservoirs with which the system exchanges heat; in order to emphasize this, we write the reservoir temperatures as $T_{R,k}$.

The form (44) is valid for *any* system **S**, in *any* state, that exchanges heat with reservoirs which have thermodynamic temperatures $T_{R,k}$. The entropy of the system is *S*, and it should be clear from the derivation that it is defined for any state, equilibrium, or nonequilibrium! Thermodynamic temperature must be positive to ensure dissipation of work due to friction. The discussion below will show that for systems in local thermal equilibrium, the reservoir temperature can be replaced by the system boundary temperature.

## 24. Entropy in Equilibrium: Gibbs Equation

Equilibrium entropy can be related to measurable quantities in a straightforward manner, so that it is measurable as well, albeit indirectly. We consider an equilibrium system undergoing a quasi-static processes, in contact with a heater at temperature *T*; for instance we might think of a carefully controlled resistance heater. Due to the equilibrium condition, the temperature of the system must be *T* as well (0th law!), and the entropy generation vanishes, ${\dot{S}}_{gen}=0$. Then, Equation (44) for entropy becomes

(45) $$\frac{dS_{E}}{dt}=\frac{\dot{Q}}{T}\;;$$

while for this case the the 1st law (45) reads

(46) $$\frac{dU_{E}}{dt}=\dot{Q}-p\frac{dV}{dt}\;.$$

In both equations we added the index *E* to highlight the equilibrium state; *p* is the homogeneous pressure of the equilibrium state.

We are only interested in an infinitesimal step of the process, of duration dt. Eliminating the heat between the two laws, we find

(47) $$TdS_{E}=dU_{E}+pdV\;.$$

This relation is known as the Gibbs equation, named after Josiah Willard Gibbs (1839–1903). The Gibbs equation is a differential relation between properties of the system and valid for *all* simple substances—in equilibrium states.

We note that *T* and *p* are intensive, and *U*, *V* and *S* are extensive properties. The specific entropy $s_{E}=S_{E}/m$ can be computed from the Gibbs equation for specific properties, which is obtained by division of (47) with the constant mass *m*. We ignore the subscript *E* for streamlined notation, so that the Gibbs equation for specific properties reads

(48) Tds=du+pdv.

Solving the first law for reversible processes (27) for heat and comparing the result with the Gibbs equation we find, with $\dot{Q}dt=\delta Q$,

(49) $$dS=\frac{1}{T}\left(dU+pdV\right)=\frac{1}{T}\delta Q\;.$$

We recall that heat is a path function, that is, δQ is an inexact differential, but entropy is a state property, that is, dS is an exact differential. In the language of mathematics, the inverse thermodynamic temperature $\frac{1}{T}$ serves as an integrating factor for δQ, such that $dS=\frac{1}{T}\delta Q$ becomes an exact differential.

It must be noted that one can always find an integrating factor for a differential form of two variables. Hence, it must be emphasized that thermodynamic temperature *T* remains an integrating factor if additional contributions to reversible work (conjugate work variables) are considered in the first law, which leads to the Gibbs equation in the form $TdS=dU-\sum_{j}x_{j}dY_{j}$, where $\left\{x_{j},Y_{j}\right\}$ are pairs of conjugate work variables, such as {p,V}. For instance, this becomes clear in Caratheodory’s axiomatic treatment of thermodynamics (for adiabatic processes) [9], which is briefly discussed in Appendix A.

From the above, we see that for reversible processes δQ=TdS. Accordingly, the total heat exchanged in a reversible process can be computed from temperature and entropy as the area below the process curve in the temperature-entropy diagram (T-S-diagram),

(50) $$Q_{12}=\int_{1}^{2}TdS\;.$$

This is analogue to the computation of the work in a reversible process as $W_{12}=\int_{1}^{2}pdV$.

## 25. Measurability of Properties

Some properties are easy to measure, and thus quite intuitive, for example, pressure *p*, temperature *T* and specific volume *v*. Accordingly, the thermal equation of state, $p\left(T,v\right)$ can be measured with relative ease, for systems in equilibrium. Other properties cannot be measured directly, for instance internal energy *u*, which must be determined by means of applying the first law to a calorimeter, or equilibrium entropy *s*, which must be determined from other properties by integration of the Gibbs Equation (48).

The Gibbs equation gives a differential relation between properties for any simple substance. Its analysis with the tools of multivariable calculus shows that specific internal energy *u*, specific enthalpy h=u+pv, specific Helmholtz free energy f=u−Ts, and specific Gibbs free energy g=h−Ts are potentials when considered as functions of particular variables. The evaluation of the potentials leads to a rich variety of relations between thermodynamic properties. In particular, these relate properties that are more difficult, or even impossible, to measure to those that are more easy to measure, and thus reduce the necessary measurements to determine data for all properties. The discussion of the thermodynamic potentials energy *u*, enthalpy *h*, Helmholtz free energy *f* and Gibbs free energy *g*, based on the Gibbs equation is one of the highlights of equilibrium thermodynamics [8,10]. Here, we refrain from a full discussion and only consider one important result in the next section.

To avoid misunderstanding, we point out that the following Section 26, Section 27, Section 28, Section 29 concern thermodynamic properties of systems in equilibrium states. We also stress that entropy and internal energy are system properties also in nonequilibrium states.

## 26. A Useful Relation

The Gibbs equation formulated for the Helmholtz free energy f=u−Ts arises from a Legendre transform $Tds=d\left(Ts\right)-sdT$ in the Gibbs equation as

(51) df=−sdT−pdv.

Hence, $f\left(T,v\right)$ is a thermodynamic potential [8,10], with

(52) $$-s={\left(\frac{\partial f}{\partial T}\right)}_{v}\;\;\;,\;\;\;-p={\left(\frac{\partial f}{\partial v}\right)}_{T}\;\;\;,\;\;\;{\left(\frac{\partial s}{\partial v}\right)}_{T}={\left(\frac{\partial p}{\partial T}\right)}_{v}\;.$$

The last equation is the Maxwell relation for this potential, it results from exchanging the order of derivatives, $\frac{\partial^{2}f}{\partial v\partial T}=\frac{\partial^{2}f}{\partial T\partial v}$. Remarkably, the Maxwell relation (52)${}_{3}$ contains the expression ${\left(\frac{\partial p}{\partial T}\right)}_{v}$, which can be interpreted as the change of pressure *p* with temperature *T* in a process at constant volume *v*. Since *p*, *T* and *v* can be measured, this expression can be found experimentally. In fact, measurement of $\left\{p,T,v\right\}$ gives the thermal equation of state $p\left(T,v\right)$, and we can say that ${\left(\frac{\partial p}{\partial T}\right)}_{v}$ can be determined from the thermal equation of state. The other expression, ${\left(\frac{\partial s}{\partial v}\right)}_{T}$, cannot be measured by itself, since it contains entropy *s*, which cannot be measured directly. Hence, with the Maxwell relation the expression ${\left(\frac{\partial s}{\partial v}\right)}_{T}$ can be measured indirectly, through measurement of the thermal equation of state.

To proceed, we consider energy and entropy in the Gibbs Equation (48) as functions of temperature and volume, $u\left(T,v\right)$, $s\left(T,v\right)$. We take the partial derivative of the Gibbs equation with respect to *v* while keeping *T* constant, to find

(53) $${\left(\frac{\partial u}{\partial v}\right)}_{T}=T{\left(\frac{\partial s}{\partial v}\right)}_{T}-p\;.$$

With the Maxwell relation (52)${}_{3}$ to replace the entropy derivative ${\left(\frac{\partial s}{\partial v}\right)}_{T}$ in (53)${}_{1}$, we find an equation for the volume dependence of internal energy that is entirely determined by the thermal equation of state $p\left(T,v\right)$,

(54) $${\left(\frac{\partial u}{\partial v}\right)}_{T}=T{\left(\frac{\partial p}{\partial T}\right)}_{v}-p\;.$$

Since internal energy cannot be measured directly, the left hand side cannot be determined experimentally. The equation states that the volume dependence of the internal energy is known from measurement of the thermal equation of state.

## 27. Thermodynamic and Ideal Gas Temperatures

In the derivation of the 2nd law, thermodynamic temperature *T* appears as the factor of proportionality between the heat transfer rate $\dot{Q}$ and the entropy transfer rate $\dot{\Gamma }$. In previous sections we have seen that this definition of thermodynamic temperature stands in agreement with the direction of heat transfer: heat flows from hot (high *T*) to cold (low *T*) by itself. The heat flow aims at equilibrating the temperature within any isolated system that is left to itself, so that two systems in thermal equilibrium have the same thermodynamic temperature. Moreover, the discussion of internal friction showed that thermodynamic temperature must be positive.

While we have claimed agreement of thermodynamic temperature with the ideal gas temperature scale in Section 9, we have yet to give proof of this. To do so, we use (54) together with the experimental result stated in Section 14, that for an ideal gas the internal energy does *not* depend on volume, but only on temperature (see also Section 35). This implies, for the ideal gas,

(55) $$0={\left(\frac{\partial u}{\partial v}\right)}_{T}=T{\left(\frac{\partial p}{\partial T}\right)}_{v}-p\;\;\;\Rightarrow \;\;\;p=T{\left(\frac{\partial p}{\partial T}\right)}_{v}\;.$$

Accordingly, ideal gas pressure must be a linear function of the thermodynamic temperature *T*,

(56) $$p=\pi \left(v\right)T\;.$$

The volume dependency $\pi \left(v\right)$ must be measured, for example, in a piston cylinder system in contact with a temperature reservoir, so that the temperature is constant. Measurements show that pressure is inversely proportional to volume, so that

(57) $$p=\pi_{0}\frac{T}{v}\;,$$

with a constant $\pi_{0}$ that fixes the thermodynamic temperature scale.

The Kelvin temperature scale, named after William Thomson, Lord Kelvin (1824–1907), historically used the triple point of water (611kPa, 0.01 ${}^{{}^{\circ}}C)$ as reference. The triple point is the unique equilibrium state at which a substance can coexist in all three phases, solid, liquid and vapor. The Kelvin scales assigns the value of $T_{Tr}=273.16\;K$ to this unique point, which can be reproduced with relative ease in laboratories, so that calibration of thermometers is consistent. With this choice, the constant $\pi_{0}$ is the specific gas constant $R=\bar{R}/M$, where $\bar{R}=8.314\;\mathrm{kJ}/$$\left(\mathrm{kmol}K\right)$ is the universal gas constant, and *M* is the molecular mass with unit $\left[\mathrm{kg}/\mathrm{kmol}\right]$ (e.g., $M_{He}=4\frac{\mathrm{kg}}{\mathrm{kmol}}$ for helium, $M_{H_{2}O}=18\frac{\mathrm{kg}}{\mathrm{kmol}}$ for water, $M_{air}=29\frac{\mathrm{kg}}{\mathrm{kmol}}$ for air), so that, as already stated in Section 11,

(58) pv=RT.

In 2018, the temperature scale became independent of the triple point of water. Instead, it is now set by fixing the Boltzmann constant $k_{B}$, which is the gas constant per particle, that is, $\bar{R}=k_{B}A_{v}$ where $A_{v}$ is the Avogadro constant [24]. At the same time, other SI units were fredefined by assigning fixed values to physical constants, including the Avogadro constant, which defines the number of particles in one mole [25].

The historic development of the 2nd law relied on the use of Carnot engines, that is, a fully reversible engine between two reservoirs, and the Carnot process—which is a particular realization of a Carnot engine. Evaluation of the Carnot cycle for an ideal gas then shows the equivalence of ideal gas temperature and thermodynamic temperature. In the present treatment, all statements about engines are derived from the laws of thermodynamics, after they are found, based on simple experience.

The positivity of thermodynamic temperature implies positive ideal gas temperature and hence positive gas pressures. In Section 41, positive thermodynamic temperature is linked to mechanical stability. The ideal gas equation provides an intuitive example for this: A gas under negative pressure would collapse, hence be in an unstable state.

## 28. Measurement of Properties

Only few thermodynamic properties can be measured easily, namely temperature *T*, pressure *p*, and volume *v*. These are related by the thermal equation of state $p\left(T,v\right)$ which is therefore relatively easy to measure.

The specific heat $c_{v}={\left(\frac{\partial u}{\partial T}\right)}_{v}$ can be determined from careful measurements. These calorimetric measurements employ the first law, where the change in temperature in response to the heat (or work) added to the system is measured.

Other important quantities, however, for example, u,h,f,g,s, cannot be measured directly. We briefly study how they can be related to measurable quantities, that is, *T*, *p*, *v*, and $c_{v}$ by means of the Gibbs equation and the differential relations derived above.

We consider the measurement of internal energy. The differential of $u\left(T,v\right)$ is

(59) $$du=c_{v}dT+{\left(\frac{\partial u}{\partial v}\right)}_{T}dv\;.$$

Therefore, the internal energy $u\left(T,v\right)$ can be determined by integration when $c_{v}$ and ${\left(\frac{\partial u}{\partial v}\right)}_{T}$ are known from measurements. By (54) the term ${\left(\frac{\partial u}{\partial v}\right)}_{T}$ is known through measurement of the thermal equation of state, and we can write

(60) $$du=c_{v}dT+\left[T{\left(\frac{\partial p}{\partial T}\right)}_{v}-p\right]dv\;.$$

Integration is performed from a reference state $\left(T_{0},v_{0}\right)$ to the actual state $\left(T,v\right)$. Since internal energy is a point function, its differential is exact, and the integration is independent of the path chosen. The easiest integration is in two steps, first at constant volume $v_{0}$ from $\left(T_{0},v_{0}\right)$ to $\left(T,v_{0}\right)$, then at constant temperature *T* from $\left(T,v_{0}\right)$ to $\left(T,v\right)$,

(61) $$u\left(T,v\right)-u\left(T_{0},v_{0}\right)=\int_{T_{0}}^{T}c_{v}\left(T^{\prime },v_{0}\right)dT^{\prime }+\int_{v_{0}}^{v}\left[T{\left(\frac{\partial p}{\partial T}\right)}_{v^{\prime }}-p\left(T,v^{\prime }\right)\right]dv^{\prime }\;.$$

Accordingly, in order to determine the internal energy $u\left(T,v\right)$ for all *T* and *v* it is sufficient to measure the thermal equation of state $p\left(T,v\right)$ for all $\left(T,v\right)$ and the specific heat $c_{v}\left(T,v_{0}\right)$ for all temperatures *T* but *only one* volume $v_{0}$. For the ideal gas, the volume contribution vanishes, and the above reduces to (20).

The internal energy can only be determined apart from a reference value $u\left(T_{0},v_{0}\right)$. As long as no chemical reactions occur, the energy constant $u\left(T_{0},v_{0}\right)$ can be arbitrarily chosen.

Entropy $s\left(T,v\right)$ follows by integration of the Gibbs equation, for example, in the form, again with (54),

(62) $$ds=\frac{1}{T}du+\frac{p}{T}dv=\frac{c_{v}}{T}dT+\frac{1}{T}\left({\left(\frac{\partial u}{\partial v}\right)}_{T}+p\right)dv=\frac{c_{v}}{T}dT+{\left(\frac{\partial p}{\partial T}\right)}_{v}dv\;,$$

as

(63) $$s\left(T,v\right)-s\left(T_{0},v_{0}\right)=\int_{T_{0}}^{T}\frac{c_{v}\left(T^{\prime },v_{0}\right)}{T^{\prime }}dT^{\prime }+\int_{v_{0}}^{v}{\left(\frac{\partial p}{\partial T}\right)}_{v^{\prime }}dv^{\prime }\;;$$

Also entropy can be determined only apart from a reference value $s\left(T_{0},v_{0}\right)$ which only plays a role when chemical reactions occur; the third law of thermodynamics fixes the scale properly.

After *u* and *s* are determined, enthalpy *h*, Helmholtz free energy *f*, and Gibbs free energy *g* simply follow by means of their definitions. Thus the measurement of *all* thermodynamic quantities requires only the measurement of the thermal equation of state $p\left(T,v\right)$ for all $\left(T,v\right)$ and the measurement of the specific heat at constant volume $c_{v}\left(T,v_{0}\right)$ for all temperatures, but only one volume, for example, in a constant volume calorimeter. All other quantities follow from differential relations that are based on the Gibbs equation, and integration [8,10].

Above we have outlined the necessary measurements to fully determine all relevant thermodynamic properties for systems in equilibrium. We close this section by pointing out that all properties can be determined if just one of the thermodynamic potentials u,h,f,g is known [8,10]. Since all properties can be derived from the potential, the expression for the potential is sometimes called the *fundamental relation*.

## 29. Property Relations for Entropy

For incompressible liquids and solids, the specific volume is constant, hence dv=0. The caloric equation of state (59) implies $du=c_{v}dT$ and the Gibbs equation reduces to $Tds=c_{v}dT$. For constant specific heat, $c_{v}=const.$, integration gives entropy as explicit function of temperature,

(64) $$s\left(T\right)=c_{v}\ln\frac{T}{T_{0}}+s_{0}\;,$$

where $s_{0}$ is the entropy at the reference temperature $T_{0}$.

For the ideal gas, where ${\left(\frac{\partial p}{\partial T}\right)}_{p}=\frac{R}{v}$ and the specific heat depends on *T* only, entropy assumes the familiar form

(65) $$s\left(T,v\right)=\int_{T_{0}}^{T}\frac{c_{v}\left(T^{\prime }\right)}{T^{\prime }}dT^{\prime }+R\ln\frac{v}{v_{0}}+s_{0}\;,$$

For a gas with constant specific heat, the integration can be performed to give

(66) $$s\left(T,v\right)=c_{v}\ln\frac{T}{T_{0}}+R\ln\frac{v}{v_{0}}+s_{0}\;.$$

Of course, a substance behaves as an ideal gas only for sufficiently low pressures or sufficiently hight temperatures, so that these relations have a limited range of applicability. In particular for low temperatures, the ideal gas law and the equations above are not valid.

## 30. Local Thermodynamic Equilibrium

In the previous sections, we considered homogeneous systems that undergo equilibrium processes, and discussed how to determine thermodynamic properties of systems in equilibrium states. To generalize for processes in inhomogeneous systems, we now consider the system as a compound of sufficiently small subsystems. The key assumption is that each of the subsystems is in *local thermodynamic equilibrium*, so that it can be characterized by the same state properties as a macroscopic equilibrium system. To simplify the proceedings somewhat, we consider numbered subsystems of finite size, and summation.

The exact argument for evaluation of local thermodynamic equilibrium considers infinitesimal cells dV, partial differential equations, and, to arrive at the equations for systems, integration. This detailed approach, known as *Linear Irreversible Thermodynamics* (LIT), is presented in Appendix C. The simplified argument below avoids the use of partial differential equations, and aims only on the equations for systems, hence this might be the preferred approach for use in an early undergraduate course [8].

**Figure 8:**
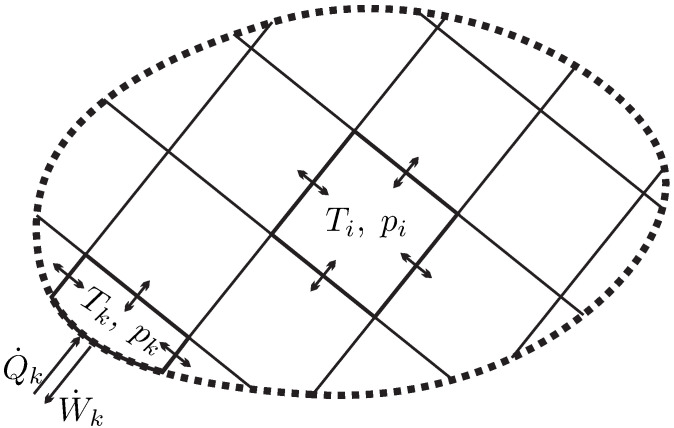
Non-equilibrium system split into small equilibrium subsystems. Arrows indicate work and heat exchange between neighboring elements, and the surroundings.

Figure 8 indicates the splitting into subsystems, and highlights a subsystem *i* inside the system and a subsystem *k* at the system boundary. Temperature and pressure in the subsystems are given by $T_{i}$, $p_{i}$ and $T_{k}$, $p_{k}$, respectively. Generally, temperature and pressure are inhomogeneous, that is adjacent subsystems have different temperatures and pressures. Accordingly, each subsystem interacts with its neighborhood through heat and work transfer as indicated by the arrows. Heat and work exchanged with the surroundings of the system are indicated as ${\dot{Q}}_{k}$ and ${\dot{W}}_{k}$.

Internal energy and entropy in a subsystem *i* are denoted as $E_{i}$ and $S_{i}$, and, since both are extensive, the corresponding quantities for the complete system are obtained by summation over all subsystems, $E=\sum_{i}E_{i}$, $S=\sum_{i}S_{i}$. Note that in the limit of infinitesimal subsystems the sums become integrals, as in Section 5. The balances of energy and entropy for a subsystem *i* read

(67) $$\frac{dE_{i}}{dt}={\dot{Q}}_{i}-{\dot{W}}_{i}\;\;\;,\;\;\;\frac{dS_{i}}{dt}=\frac{{\dot{Q}}_{i}}{T_{i}}+{\dot{S}}_{gen,i}\;,$$

where ${\dot{Q}}_{i}=\sum_{j}{\dot{Q}}_{i,j}$ is the net heat exchange, and ${\dot{W}}_{i}=\sum_{j}{\dot{W}}_{i,j}$ is the net work exchange for the subsystem. Here, the summation over *j* indicates the exchange of heat and work with the neighboring cells, such that, for example, ${\dot{Q}}_{i,j}$ is the heat that *i* receives from the neighboring cell *j*.

The boundary cells of temperatures $T_{k}$ are either adiabatically isolated to the outside, or they exchange heat with external systems (reservoirs) of temperature $T_{R,k}$, which, in fact, are the temperatures that appear in the 2nd law in the form of Equation (44). For systems in local thermodynamic equilibrium, temperature differences at boundaries, such at those between a gas and a container wall, are typically extremely small. Hence, temperature jumps at boundaries are usually ignored, so that $T_{R,k}=T_{k}$, and we will proceed with this assumption. Appendix C.4 provides a more detailed discussion of temperature jumps and velocity slip within the context of Linear Irreversible Thermodynamics.

To obtain first and second law for the compound system, we have to sum the corresponding laws for the subsystems, which gives

(68) $$\frac{dE}{dt}=\dot{Q}-\dot{W}\;\;\;\mathrm{with}\;\;\;\dot{Q}=\sum_{k}{\dot{Q}}_{k}\;,\;\dot{W}=\sum_{k}{\dot{W}}_{k}$$

and

(69) $$\frac{dS}{dt}=\sum_{k}\frac{{\dot{Q}}_{k}}{T_{k}}+{\dot{S}}_{gen}\;\;\;\mathrm{with}\;\;\;{\dot{S}}_{gen}\geq 0\;.$$

In the above, ${\dot{Q}}_{k}$ is the heat transferred over a system boundary which has temperature $T_{k}$. This subtle change from Equation (44), which has the reservoir temperatures, results from ignoring temperature jumps at boundaries. As will be explained next, the summation over *k* concerns only heat and work exchange with the surroundings.

Since energy is conserved, the internal exchange of heat and work between subsystems cancels in the conservation law for energy (68). For instance, in the exchange between neighboring subsystems *i* and *j*, $Q_{i,j}$ is the heat that *i* receives from *j* and $W_{i,j}$ is the work that *i* does on *j*. Moreover, $Q_{j,i}$ is the heat that *j* receives from *i* and $W_{j,i}$ is the work that *j* does on *i*. Since energy is conserved, no energy is added or lost in transfer between *i* and *j*, that is $Q_{i,j}=-Q_{j,i}$ and $W_{i,j}=-W_{j,i}$. Accordingly, the sums vanish, $Q_{i,j}+Q_{j,i}=0$ and $W_{i,j}+W_{j,i}=0$. Extension of the argument shows that the internal exchange of heat and work between subsystems adds up to zero, so that only exchange with the surroundings, indicated by subscript *k*, appears in (68).

Entropy, however, is not conserved, but may be produced. Exchange of heat and work between subsystems, if irreversible, will contribute to the entropy generation rate ${\dot{S}}_{gen}$. Thus, the total entropy generation rate ${\dot{S}}_{gen}$ of the compound system is the sum of the entropy generation rates in the subsystems ${\dot{S}}_{gen,i}$ plus additional terms related to the energy transfer between subsystems, ${\dot{S}}_{gen}=\sum_{i}{\dot{S}}_{gen,i}+\sum_{i,j}{\dot{S}}_{gen,i,j}>0$. In simple substances, this internal entropy generation occurs due to internal heat flow and internal friction.

Strictly speaking, the small temperature differences for heat transfer between system and boundary, $T_{k}-T_{R,k}$ contribute to entropy generation as well. In typical applications, the temperature differences and the associated entropy generation are so small that both can be ignored.

We repeat that entropy generation is strictly positive, ${\dot{S}}_{gen}>0$, in irreversible processes, and is zero, ${\dot{S}}_{gen}=0$, in reversible processes.

To fully quantify entropy generation, that is to compute its actual value, requires the detailed local computation of all processes inside the system from the conservation laws and the second law as partial differential equations—this is outlined in Appendix C.

The above derivation of the second law Equation (69) relies on the assumption that the equilibrium property relations for entropy are valid locally also for nonequilibrium systems. This *local equilibrium hypothesis*—equilibrium in a subsystem, but not in the compound system—works well for most systems in technical thermodynamics. It should be noted that the assumption breaks down for extremely strong nonequilibrium.

## 31. Heat Transfer between Reservoirs

In this and the following sections we proceed by considering simple processes with the 1st and 2nd law in the form (68) and (69) found for systems in local thermodynamic equilibrium. These examples are shown to highlight the contents of the 2nd law. For instructors who prefer to *postulate* the 2nd law, these would be the examples used to show the agreement with daily experience.

**Figure 9:**
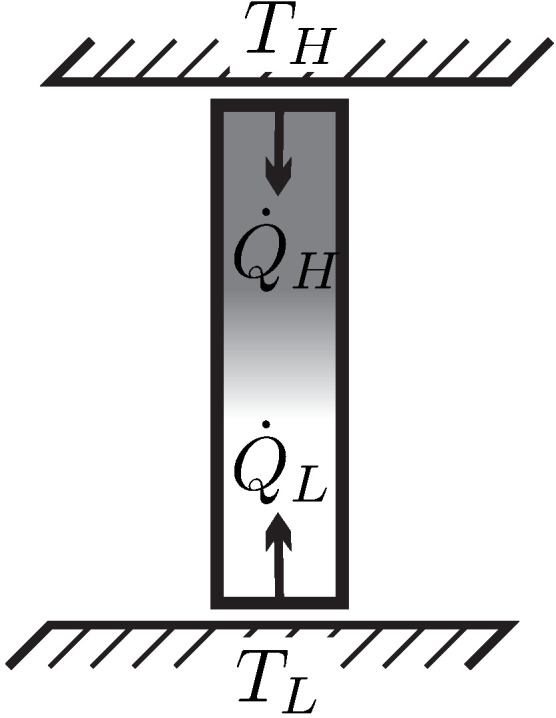
Heat transfer between two reservoirs at $T_{1}$ and $T_{2}$. In steady state the heat conductor does not accumulate energy, therefore ${\dot{Q}}_{L}=-{\dot{Q}}_{H}$.

We begin with the basic heat transfer process between two reservoirs of thermodynamic temperatures $T_{H}$ and $T_{L}$, where $T_{H}>T_{L}$ is the temperature of the hotter system, see Figure 9. The heat is transferred through a heat conductor, which is the thermodynamic system to be evaluated. One will expect a temperature gradient in the conductor, that is the conductor is not in a homogeneous equilibrium state, but in a nonequilibrium state. A pure heat transfer problem is studied, where the conductor receives the heat flows ${\dot{Q}}_{H}$ and ${\dot{Q}}_{L}$, and exchanges no work with the surroundings, $\dot{W}=0$. The first and second law (68) and (69) applied to the heat conductor read

(70) $$\frac{dU}{dt}={\dot{Q}}_{L}+{\dot{Q}}_{H}\;\;\;,\;\;\;\frac{dS}{dt}-\frac{{\dot{Q}}_{L}}{T_{L}}-\frac{{\dot{Q}}_{H}}{T_{H}}={\dot{S}}_{gen}\geq 0\;.$$

For steady state conditions no changes over time are observed in the conductor, so that $\frac{dU}{dt}=\frac{dS}{dt}=0$. The first law shows that the heat flows must be equal in absolute value, but opposite in sign,

(71) $${\dot{Q}}_{H}=-{\dot{Q}}_{L}=\dot{Q}\;.$$

With this, the second law reduces to the inequality

(72) $$\dot{Q}\left(\frac{1}{T_{L}}-\frac{1}{T_{H}}\right)={\dot{S}}_{gen}\geq 0\;.$$

With the thermodynamic temperature $T_{H}>T_{L}>0$, the bracket is positive. According to Figure 9 the proper direction of heat transfer in accordance to Clausius’ statement that *heat will go from hot to cold by itself, but not vice versa* (**Observation 5**) is for ${\dot{Q}}_{H}=-{\dot{Q}}_{L}=\dot{Q}>0$.

**Figure 10:**
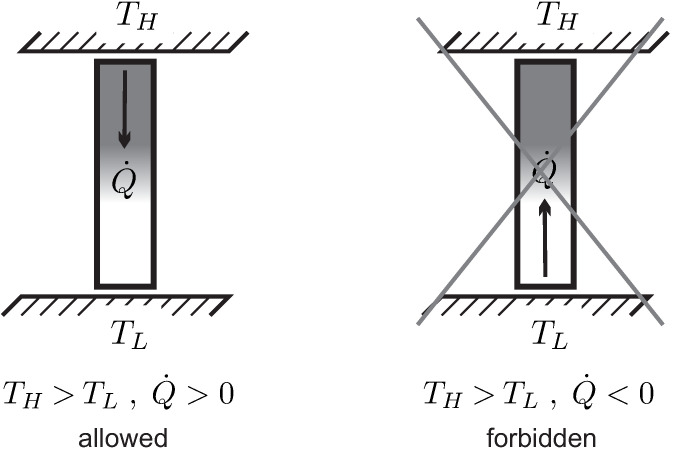
Heat transfer between two reservoirs with $T_{H}>T_{L}$. Heat must go from warm to cold.

Equation (72) shows that heat transfer over finite temperature differences creates entropy inside the heat conductor. In the steady state case considered here, the entropy created is leaving the system with the outgoing entropy flow $\frac{{\dot{Q}}_{L}}{T_{L}}$ which is larger than the incoming entropy flow $\frac{{\dot{Q}}_{H}}{T_{H}}$. Figure 10 gives an illustration of the allowed process, where heat goes from hot to cold, and the forbidden process, where heat would go from cold to hot by itself.

## 32. Newton’s Law of Cooling

The inequality (72) requires that $\dot{Q}$ has the same sign as $\left(\frac{1}{T_{L}}-\frac{1}{T_{H}}\right)$, a requirement that is fulfilled for a heat transfer rate

(73) $$\dot{Q}=\alpha A\left(T_{H}-T_{L}\right)$$

with a positive heat transfer coefficient α>0, and the heat exchange surface area *A*. This relation is known as Newton’s law of cooling, and is often used in heat transfer problems. The values of the positive coefficient α must be found from the detailed configuration and conditions in the heat transfer system. The surface area *A* appears due to the intuitive expectation that enlarging the transfer area leads to a proportional increase in the amount of heat transferred.

Heat transfer was introduced as energy transfer due to temperature difference with heat going from hot to cold. Newton laws of cooling states that as a result of the temperature difference one will observe a response, namely the heat flow.

The procedure to deduce Newton’s law of cooling can be described as follows: The entropy generation rate (72) is interpreted as the product of a thermodynamic force—here, the difference of inverse temperatures $\left(\frac{1}{T_{L}}-\frac{1}{T_{H}}\right)$—and a corresponding flux—here, the heat flow $\dot{Q}$. To ensure positivity of the entropy generation rate, the flux must be proportional to the force, with a positive factor αA that must be measured. This is the strategy of Linear Irreversible Thermodynamics, which can be used for all force-flux pairs, see Appendix C. A thermodynamic force is any deviation from the equilibrium state, here the temperature difference, which will vanish in equilibrium. A thermodynamic flux is a response to the force that drives a process towards equilibrium, here the heat flux.

With Newton’s law of cooling it is easy to see that heat transfer over finite temperature differences is an irreversible process. Indeed, the second law (72) gives with (73)

(74) $${\dot{S}}_{gen}=\dot{Q}\left(\frac{1}{T_{L}}-\frac{1}{T_{H}}\right)=\alpha A\frac{{\left(T_{H}-T_{L}\right)}^{2}}{T_{L}T_{H}}>0\;.$$

Equation (74) quantifies the entropy generation rate in steady state heat transfer, which, for fixed heat transfer rate $\dot{Q}$, grows with the difference of inverse temperatures. Only when the temperature difference is infinitesimal, that is, $T_{H}=T_{L}+dT$, entropy generation can be ignored, and heat transfer can be considered as a reversible process. This can be seen as follows: For infinitesimal dT the entropy generation rate becomes ${\dot{S}}_{gen}=\alpha A{\left(\frac{dT}{T_{L}}\right)}^{2}$ and heat becomes $\dot{Q}=\alpha AdT$. This implies that entropy generation vanishes with the temperature difference, ${\dot{S}}_{gen}=0\;\left(dT\to 0\right)$. In this case, to have a finite amount of heat $\dot{Q}$ transferred, the heat exchange area *A* must go to infinity.

## 33. 0th Law and 2nd Law

Above we considered heat transfer between reservoirs, but the conclusion is valid for heat conduction between arbitrary systems: As long as the systems are in thermal contact through heat conductors, and their temperatures are different, there will be heat transfer between the systems. Only when the temperatures of the systems are equal, heat transfer will cease. This is the case of thermal equilibrium, where no change in time occurs anymore. This includes that the temperature of an isolated body in thermal equilibrium will be homogeneous, where equilibration occurs through heat transfer within the system; for the formal argument see Section 40 below.

The 0th law states that in equilibrium systems in thermal contact assume the same temperature. Thus, the 0th law of thermodynamics might appear as a special case of the 2nd law. It stands in its own right, however: Not only does it define temperature as a measurable quantity, but it also states the homogeneity of temperature in equilibrium, which is required to identify the Gibbs equation in Section 24.

## 34. Internal Friction

When coffee, or any other liquid, is stirred, it will spin a while after the spoon is removed. The motion will slow down because of internal friction, and finally the coffee will be at rest in the cup. We show that the 2nd law describes this well-known behavior, which is observed in all viscous fluids.

**Figure 11:**
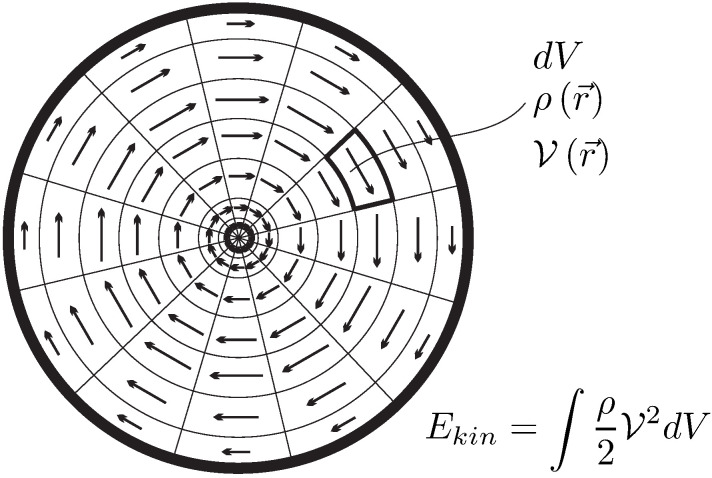
The kinetic energy $E_{kin}$ of a stirred fluid is the sum of the kinetic energies in all volume elements. Friction with the container wall, and within the fluid, will slow down the fluid until it comes to rest in the final equilibrium state.

With the fluid in motion, all fluid elements have different velocity vectors, that is, the system is not in a homogeneous equilibrium state. We have to account for the kinetic energy of the swirling, which must be computed by summation, that is, integration, of the local kinetic energies $\frac{\rho \left(\vec{r}\right)}{2}V{\left(\vec{r}\right)}^{2}$ in all volume elements; see Figure 11. The 1st and 2nd law (68) and (69) now read

(75) $$\frac{d\left(U+E_{kin}\right)}{dt}=\dot{Q}-\dot{W}\;\;\;,\;\;\;\frac{dS}{dt}-\sum \frac{{\dot{Q}}_{k}}{T_{k}}={\dot{S}}_{gen}\geq 0\;.$$

We assume adiabatic systems ($\dot{Q}=0$) without any work exchange ($\dot{W}=0$, this implies constant volume), so that

(76) $$\frac{d\left(U+E_{kin}\right)}{dt}=0\;\;\;,\;\;\;\frac{dS}{dt}={\dot{S}}_{gen}\geq 0\;.$$

For simplicity we ignore local temperature differences within the stirred substance, and use the Gibbs Equation (47) so that

(77) $$\frac{dS}{dt}={\left(\frac{\partial S}{\partial U}\right)}_{V}\frac{dU}{dt}=\frac{1}{T}\frac{dU}{dt}=-\frac{1}{T}\frac{dE_{kin}}{dt}={\dot{S}}_{gen}\geq 0\;.$$

Since entropy generation and inverse thermodynamic temperature are non-negative, this implies

(78) $$\frac{dE_{kin}}{dt}⩽0\;.$$

Hence, the kinetic energy $E_{kin}=\int \frac{\rho }{2}V^{2}dV$ decreases over time, and will be zero in equilibrium, where the stirred substance comes to rest, V=0.

Here we notice, again, that the sign of thermodynamic temperature is intimately linked to friction: T>0 ensures that friction dissipates kinetic energy. The total entropy generation in this process is

(79) $$S_{gen}=\int {\dot{S}}_{gen}dt=-\int \frac{1}{T}\frac{dE_{kin}}{dt}dt\;.$$

## 35. Uncontrolled Expansion of a Gas

**Figure 12:**
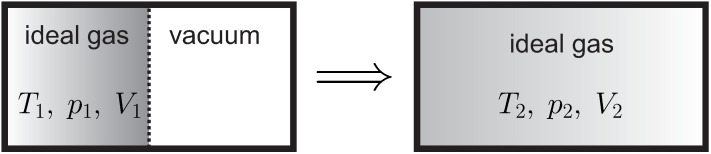
Irreversible adiabatic expansion of an ideal gas.

Our next example concerns the uncontrolled expansion of an ideal gas. We consider an ideal gas in a container which is divided by a membrane, see Figure 12.

Initially the gas is contained in one part of the container at $\left\{T_{1},p_{1},V_{1}\right\}$, while the other part is evacuated. The membrane is destroyed, and the gas expands to fill the the container. The fast motion of the gas is slowed down by internal friction, and in the final homogeneous equilibrium state $\left\{T_{2},p_{2},V_{2}\right\}$ the gas is at rest and distributed over the total volume of the container. We have no control over the flow after the membrane is destroyed: this is an irreversible process.

The container is adiabatically enclosed to the exterior, and, since its walls are rigid, no work is transmitted to the exterior. Thus, the first law for closed systems (11) reduces to

(80) $$\frac{d\left(U+E_{kin}+E_{pot}\right)}{dt}=0\;,$$

or, after integration,

(81) $$U_{2}+E_{kin,2}+E_{pot,2}=U_{1}+E_{kin,1}+E_{pot,1}\;.$$

Since the gas it at rest initially and in the end, $E_{kin,1}=E_{kin,2}=0$, and since potential energy has not changed $E_{pot,1}=E_{pot,2}$, the above reduces to $U_{2}=U_{1}$. Note, however, that *during* the process $E_{kin}>0$ and $U<U_{1}$.

With U=mu, and m=const., the specific internal energy remains unchanged,

(82) $$u\left(T_{1},v_{1}\right)=u\left(T_{2},v_{2}\right)\;.$$

Measurements for ideal gases show that $T_{1}=T_{2}$, that is the initial and final temperatures of the gas are the same. With this, the previous condition becomes

(83) $$u\left(T_{1},v_{1}\right)=u\left(T_{1},v_{2}\right)\;,$$

which can only hold if the internal energy of the ideal gas does not depend on volume. This experiment verifies that the internal energy of the ideal gas is independent of volume, and depends only on temperature, $u=u\left(T\right)$. In Section 27 we already used this result to show the equivalence of thermodynamic and ideal gas temperature scales.

The second law for this adiabatic process simply reads

(84) $$\frac{dS}{dt}={\dot{S}}_{gen}\geq 0\;.$$

Integration over the process duration yields

(85) $$S_{2}-S_{1}=\int_{t_{1}}^{t_{2}}{\dot{S}}_{gen}dt=S_{gen}\geq 0\;.$$

The total change of entropy follows from the ideal gas entropy (66), with $T_{1}=T_{2}$, as

(86) $$S_{2}-S_{1}=m\left(s_{2}-s_{1}\right)=mR\ln\frac{V_{2}}{V_{1}}=mR\ln\frac{v_{2}}{v_{1}}>0\;.$$

Since in this process the temperature of the ideal gas remains unchanged, the growth of entropy is only attributed to the growth in volume: by filling the larger volume $V_{2}$, the gas assumes a state of larger entropy. Since the container is adiabatic, there is no transfer of entropy over the boundary (i.e., $\sum \frac{{\dot{Q}}_{k}}{T_{k}}=0$), and all entropy generated stays within the system, $S_{gen}=S_{2}-S_{1}$.

In this computation, energy and entropy change, and the entropy generated can be determined from the initial and final equilibrium states. However, the process is irreversible, with states of strong nonequilibrium along the way. The rate equations for 1st and 2nd law are valid throughout the process, but do not suffice to determine values for energy and entropy at all moments in time, since they do not allow to resolve the inhomogeneity of the intermediate states. A detailed prediction of the process requires a local theory, such as the Navier-Stokes-Fourier equations of Linear Irreversible Thermodynamics (see Appendix C), or the Boltzmann equation of Kinetic Gas Theory (see Appendix D). Values for system energy and entropy can be obtained from the local description through integration over the system.

## 36. Irreversibility and Work Loss

The thermodynamic laws for closed systems that exchange heat with an arbitrary number of reservoirs read

(87) $$\frac{d\left(U+E_{kin}\right)}{dt}={\dot{Q}}_{0}+\sum {\dot{Q}}_{k}-\dot{W}\;\;\;,\;\;\;\frac{dS}{dt}-\frac{{\dot{Q}}_{0}}{T_{0}}-\sum \frac{{\dot{Q}}_{k}}{T_{k}}={\dot{S}}_{gen}\geq 0\;,$$

where the heat exchange ${\dot{Q}}_{0}$ with a reservoir at $T_{0}$ is highlighted. Most thermodynamic engines utilize the environment as heat source or sink, and in this case ${\dot{Q}}_{0}$ should be considered as the heat exchanged with the environment. Note that the environment is freely available, and no cost is associated with removing heat from, or rejecting heat into, the environment. Moreover the environment is large compared to any system interacting with it, hence its temperature $T_{0}$ remains constant.

Elimination of ${\dot{Q}}_{0}$ between the two laws and solving for work gives

(88) $$\dot{W}=\sum \left(1-\frac{T_{0}}{T_{k}}\right){\dot{Q}}_{k}-\frac{d\left(U+E_{kin}-T_{0}S\right)}{dt}-T_{0}{\dot{S}}_{gen}\;.$$

This equation applies to arbitrary processes in closed systems. The generation of entropy in irreversible processes reduces the work output of work producing devices (where $\dot{W}>0$, for example, heat engines) and increases the work requirement of work consuming devices (where $\dot{W}<0$, for example, heat pumps and refrigerators). We note the appearance of the Carnot factor $\left(1-\frac{T_{0}}{T_{k}}\right)$ multiplying the heating rates ${\dot{Q}}_{k}$.

The amount of work lost to irreversible processes is

(89) $${\dot{W}}_{\mathrm{loss}}=T_{0}{\dot{S}}_{gen}\geq 0\;,$$

sometimes it is denoted as the *irreversibility*. It is an important engineering task to identify and quantify the irreversible work losses, and to reduce them by redesigning the system, or use of alternative processes. Loss analysis is an important part of technical thermodynamics that is featured in modern textbooks [8,23].

Entropy generation is due to friction, heat transfer over finite temperature differences, mixing, chemical reactions, and so forth. Full quantification of the entropy generation in nonequilibrium processes requires resolution of the process at all times, that is, solution of local transport equations (Navier-Stokes-Fourier, etc.). Nevertheless, already at the system level, loss analysis can lead to deeper insight into possibilities for process improvement.

The discussion of heat engines, refrigerators and heat pumps operating at steady state between two reservoirs, in particular of Carnot engines, is an important element of thermodynamic analysis. With the 1st and 2nd law in place, this is a special case of the above Equation (88), as is discussed next.

## 37. Heat Engines, Refrigerators, Heat Pumps

**Figure 13:**
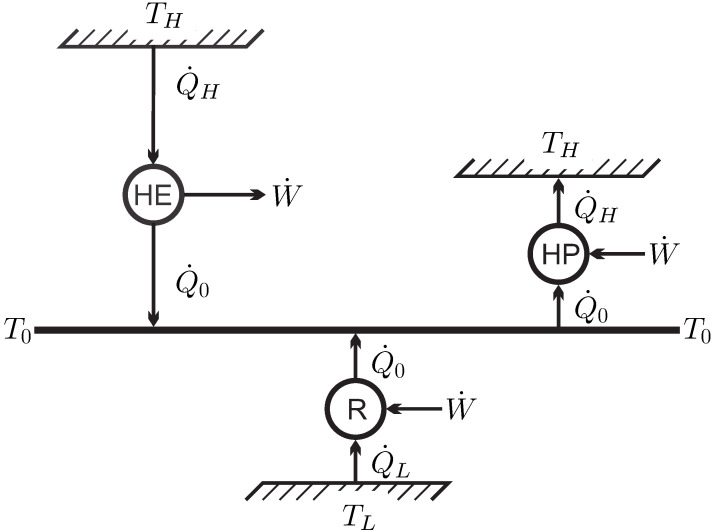
Heat engine (HE), refrigerator (R), and heat pump (HP) in contact with the environment at $T_{0}$.

Engines operating at steady state between two reservoirs, one of them the environment, are shown in Figure 13, namely a heat engine (HE), a refrigerator (R), and a heat pump (HP). We discuss these engines with the combined 1st and 2nd law (88).

For a heat engine that receives the heat ${\dot{Q}}_{H}$ from a hot reservoir at $T_{H}>T_{0}$, and rejects heat into the environment at $T_{0}$, the actual power produced is

(90) $$\dot{W}=\left(1-\frac{T_{0}}{T_{H}}\right){\dot{Q}}_{H}-T_{0}{\dot{S}}_{gen}>0\;,$$

where $T_{0}{\dot{S}}_{gen}\geq 0$ is work loss to irreversibilities. A Carnot engine, named after Sadi Carnot (1796–1832), is a fully reversible engine, that is, it has no irreversible losses and provides the power

(91) $${\dot{W}}_{\mathrm{rev}}=\left(1-\frac{T_{0}}{T_{H}}\right){\dot{Q}}_{H}>0\;.$$

The thermal efficiency is defined as the ratio of work produced (the gain) over heat input (the expense), $\eta =\dot{W}/{\dot{Q}}_{H}$, and we find the thermal efficiency of the Carnot engine as

(92) $$\eta_{C}=\frac{{\dot{W}}_{\mathrm{rev}}}{{\dot{Q}}_{H}}=1-\frac{T_{0}}{T_{H}}<1\;.$$

For a refrigerator that removes the heat ${\dot{Q}}_{L}$ from a cold space at $T_{L}<T_{0}$ and rejects heat into the environment at $T_{0}$, the power requirement is

(93) $$\dot{W}=\left(1-\frac{T_{0}}{T_{L}}\right){\dot{Q}}_{L}-T_{0}{\dot{S}}_{gen}<0\;,$$

where $T_{0}{\dot{S}}_{gen}\geq 0$ is the extra work required to overcome irreversibilities. A fully reversible refrigerator, that is, a Carnot refrigerator, requires the power

(94) $${\dot{W}}_{\mathrm{rev}}=\left(1-\frac{T_{0}}{T_{L}}\right){\dot{Q}}_{L}<0\;.$$

The coefficient of performance of a refrigerator is defined as the ratio of heat drawn from the cold (the gain) over work input (the expense), ${\mathrm{COP}}_{R}={\dot{Q}}_{L}/\left|\dot{W}\right|$, and we find the coefficient of performance of the Carnot refrigerator as

(95) $${\mathrm{COP}}_{R,C}=\frac{{\dot{Q}}_{L}}{\left|{\dot{W}}_{\mathrm{rev}}\right|}=\frac{1}{\frac{T_{0}}{T_{L}}-1}\;.$$

For a heat pump that supplies the heat ${\dot{Q}}_{H}$ to a warm space at $T_{H}>T_{0}$ and draws heat from the environment at $T_{0}$, the power requirement is

(96) $$\dot{W}=\left(1-\frac{T_{0}}{T_{H}}\right){\dot{Q}}_{H}-T_{0}{\dot{S}}_{gen}<0\;,$$

where $T_{0}{\dot{S}}_{gen}\geq 0$ is the extra work required to overcome irreversibilities. A fully reversible heat pump, that is, a Carnot heat pump, requires the power

(97) $${\dot{W}}_{\mathrm{rev}}=\left(1-\frac{T_{0}}{T_{H}}\right){\dot{Q}}_{H}<0\;.$$

The coefficient of performance of a heat pump is defined as the ratio between the heat provided (the gain) and the work input (the expense), ${\mathrm{COP}}_{\mathrm{HP}}=\left|{\dot{Q}}_{H}\right|/\left|\dot{W}\right|$, and we find the coefficient of performance of the Carnot heat pump as

(98) $${\mathrm{COP}}_{\mathrm{HP},C}=\frac{\left|{\dot{Q}}_{H}\right|}{\left|{\dot{W}}_{\mathrm{rev}}\right|}=\frac{1}{1-\frac{T_{0}}{T_{H}}}>1\;.$$

Due to irreversible losses, real engines always have lower efficiencies or coefficients of performance than the (fully reversible) Carnot engines operating between the same temperatures. While Carnot efficiencies cannot be reached—*all* real engines are irreversible—they serve as important benchmarks.

It must be emphasized that in the present approach the discussion of engines comes well after the 2nd law of thermodynamics is established. The classical Carnot-Clausius argument for finding the 2nd law puts engines front and center, which requires long discussion of processes and cycles before the 2nd law can be finally presented [2,3,4,5]. The present approach, where the 2nd law is derived from simple **Observations 1–5**, requires far less background and allows to introduce the 2nd law soon after the 1st law, so that both laws are available for the evaluation of all processes, cycles and engines right away, see also References [6,7,8]. Note that the above analysis of heat engine, refrigerator and heat pump does not require any details on the processes inside the engines.

## 38. Kelvin-Planck and Clausius Statements

The temperature difference between reservoirs provides the thermodynamic force that induces heat flux from hot to cold, due to the desire to equilibrate temperature. A heat engine converts a portion of this heat flux into work. That is, the nonequilbrium between reservoirs is essential for the process.

Not all heat received from the hot reservoir can be converted into work, some heat must be rejected to a colder reservoir. The Kelvin-Planck formulation of the second law states this as: *No steady state thermodynamic process is possible in which heat is completely converted into work*.

**Figure 14:**
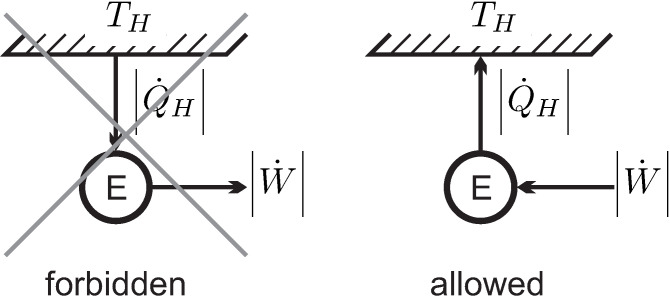
Heat cannot be completely converted into work, but work can be completely converted to heat.

This statement is a direct consequence of the 1st and 2nd law. For a steady state process with just one heat exchange the laws require

(99) $$-\frac{{\dot{Q}}_{H}}{T_{H}}=-\frac{\dot{W}}{T_{H}}={\dot{S}}_{gen}\geq 0\;,$$

hence heat and work must both be negative. Figure 14 shows the forbidden process, and also the—allowed—inverse process, the complete conversion of work into heat through friction. A typical example for the latter are resistance heaters in which electrical work is converted to heat through electric resistance (heat pump with $COP_{\mathrm{RH}}=\left|{\dot{Q}}_{H}\right|/\left|\dot{W}\right|=1$).

Clausius’ statement of the second law says that *heat will not go from cold to warm by itself*. This statement was used explicitly in our development of the 2nd law (**Observation 5**). Note that the two words “by itself” are important here—a heat pump system can transfer heat from cold to warm, but work must be supplied, so the heat transfer is not “by itself.”

It is straightforward to show that both statements are equivalent [2,8].

## 39. Finding Equilibrium States

With the laws of thermodynamics now in place, we can use them to learn more about the equilibrium states that will be observed. For an isolated system, 1st and 2nd law reduce to

(100) $$\frac{dE}{dt}=0\;\;\;\;,\;\;\;\;\frac{dS}{dt}={\dot{S}}_{gen}\geq 0\;,$$

with a constant mass m in the system. Since no work is exchanged, the system volume *V* must be constant as well. According to the second law, the state of the system will change (with ${\dot{S}}_{gen}>0$) until the entropy has reached a maximum (when ${\dot{S}}_{gen}=0$), where the process is restricted by having the initial mass, momentum and energy enclosed in the system. Starting with an arbitrary inhomogeneous initial state, the approach to equilibrium is a reorganization of the local properties of the system towards the final equilibrium state, which we will determine now for a single phase system.

Total mass, energy and entropy are obtained by integration over the full system,

(101) $$m=\int_{V}\rho dV\;\;,\;\;E=\int_{V}\rho \left(u+\frac{1}{2}V^{2}+g_{n}z\right)dV\;\;,\;\;S=\int_{V}\rho sdV\;.$$

Here, ρ, *T*, V, and $u\left(\rho ,T\right)$, $s\left(\rho ,T\right)$ are the *local* values of the thermodynamic properties, that is, $\rho =\rho \left(\vec{r}\right)$, $T=T\left(\vec{r}\right),V\left(\vec{r}\right)$ and so forth, where $\vec{r}$ is the location in the volume *V* of the system, see Section 5. The gravitational acceleration $g_{n}$ should not be confused with the Gibbs free energy *g*.

Often we are interested in systems that are globally at rest, where the overall momentum $\vec{M}$ vanishes, but we might consider also systems moving with a constant velocity $\vec{v}$, so that $\vec{M}=m\vec{v}$. Since all elements of the system have their own velocity $\vec{V}\left(\vec{r}\right)$, we find the total momentum by summing over the system,

(102) $$\vec{M}=m\vec{v}=\int_{V}\rho \vec{V}dV\;;$$

here $\vec{V}\left(\vec{r}\right)$ is the local velocity vector with absolute value $V=\sqrt{\vec{V}\cdot \vec{V}}$. As long as no forces act on the system, its momentum will be constant; total momentum vanishes for a system at rest in the observer frame, $\vec{M}=0$.

The equilibrium state is the maximum of entropy *S* under the constraints of given mass *m*, momentum $\vec{M}$, and energy *E*. The best way to account for the constraints is the use of Lagrange multipliers $\Lambda_{\rho }$, ${\vec{\Lambda }}_{M}$ and $\Lambda_{E}$ to incorporate the constraints and maximize not *S* but

(103) $$\begin{array}{c} \Phi =\int_{V}\rho sdV-\Lambda_{\rho }\left(\int_{V}\rho dV-m\right)-{\vec{\Lambda }}_{M}\cdot \left(\int_{V}\rho \vec{V}dV-\vec{M}\right)\\ -\Lambda_{E}\left(\int_{V}\rho \left(u+\frac{1}{2}V^{2}+g_{n}z\right)dV-E\right)\;. \end{array}$$

The maximization of Φ will give the local values of the thermodynamic equilibrium properties $\left\{\rho ,T,V\right\}$ in terms of the Lagrange multipliers, which then must be determined from the given values of $\left\{m,\vec{M},E\right\}$.

For the solution of this problem, we employ the methods of variational calculus. For compact notation, we introduce the abbreviations $y\left(\vec{r}\right)=\left\{\rho ,\vec{V},T\right\}$ and

(104) $$X\left(y\right)=\rho \left[s-\Lambda_{\rho }-{\vec{\Lambda }}_{M}\cdot \vec{V}-\Lambda_{E}\left(u+\frac{1}{2}V^{2}+g_{n}z\right)\right]\;.$$

The equilibrium state maximizes the integral $\int_{V}X\left(y\right)dV$. We denote the equilibrium state as $y_{E}\left(\vec{r}\right)$ and consider small variations $\delta y\left(\vec{r}\right)$ from the equilibrium state, so that $y=y_{E}+\delta y$. By means of a Taylor series we find

(105) $$\int_{V}X\left(y\right)dV=\int_{V}\left[X\left(y_{E}\right)+{\left(\frac{\partial X}{\partial y}\right)}_{|E}\delta y+\frac{1}{2}{\left(\frac{\partial^{2}X}{\partial y\partial y}\right)}_{|E}\delta y\delta y\right]dV$$

Since $y_{E}$ maximizes the integral, the other terms on the right hand side must be negative for arbitrary values of the variation $\delta y\left(\vec{r}\right)$, which implies ${\left(\frac{\partial X}{\partial y}\right)}_{|E}=0$ and negative definiteness of the matrix ${\left(\frac{\partial^{2}X}{\partial y\partial y}\right)}_{|E}$. We proceed with the evaluation of the first condition, and leave the discussion of the second conditions for Section 41.

The conditions for equilibrium read

(106) $${\left(\frac{\partial X}{\partial y}\right)}_{|E}={\left\{\frac{\partial X}{\partial \rho },\frac{\partial X}{\partial \vec{V}},\frac{\partial X}{\partial T}\right\}}_{|E}=0\;.$$

These and the following relations are valid for the equilibrium values, $T_{|E}$, $\rho_{|E}$, $u_{|E}$, $V_{|E}$, $s_{|E}$ and so forth. For better readability, the subscripts |E referring to the equilibrium state are not shown. Evaluation yields

(107) $$\frac{\partial X}{\partial \rho }=\left[s-\Lambda_{\rho }-{\vec{\Lambda }}_{M}\cdot \vec{V}-\Lambda_{E}\left(u+\frac{1}{2}V^{2}+g_{n}z\right)\right]+\rho \left[{\left(\frac{\partial s}{\partial \rho }\right)}_{T}-\Lambda_{E}{\left(\frac{\partial u}{\partial \rho }\right)}_{T}\right]=0\;,$$

(108) $$\frac{\partial X}{\partial \vec{V}}=\rho \left[-{\vec{\Lambda }}_{M}-\Lambda_{E}\vec{V}\right]=0\;.$$

(109) $$\frac{\partial X}{\partial T}=\rho \left[{\left(\frac{\partial s}{\partial T}\right)}_{\rho }-\Lambda_{E}{\left(\frac{\partial u}{\partial T}\right)}_{\rho }\right]=0\;.$$

## 40. Homogeneous Equilibrium States

We proceed with evaluating the three conditions (107)–(109) to find the stable equilibrium state. For convenience, we begin with the middle Equation (108), which gives homogeneous velocity in equilibrium,

(110) $$\vec{V}=-\frac{{\vec{\Lambda }}_{M}}{\Lambda_{E}}\;.$$

For the case of a system at rest, where

(111) $$0=\vec{M}=\int_{V}\rho \vec{V}dV=-\frac{{\vec{\Lambda }}_{M}}{\Lambda_{E}}\int_{V}\rho dV=-\frac{{\vec{\Lambda }}_{M}}{\Lambda_{E}}m\;,$$

this implies that in equilibrium all local elements are at rest, $\vec{V}={\vec{\Lambda }}_{M}=\vec{M}=0.$

With the Gibbs equation, the last condition (109) becomes

(112) $${\left(\frac{\partial s}{\partial T}\right)}_{\rho }-\Lambda_{E}{\left(\frac{\partial u}{\partial T}\right)}_{\rho }=\frac{1}{T}{\left(\frac{\partial u}{\partial T}\right)}_{\rho }-\Lambda_{E}{\left(\frac{\partial u}{\partial T}\right)}_{\rho }=0\;.$$

It follows that in equilibrium the temperature is homogeneous, and equal to the inverse Lagrange multiplier,

(113) $$T=\frac{1}{\Lambda_{E}}\;.$$

To evaluate the first condition, (107), we insert the above results for $\Lambda_{E}$, ${\vec{\Lambda }}_{M}$, $\vec{V}$ and use again the Gibbs equation, which gives ${\left(\frac{\partial s}{\partial \rho }\right)}_{T}-\frac{1}{T}{\left(\frac{\partial u}{\partial \rho }\right)}_{T}=-\frac{p}{T\rho^{2}}$. After some reordering, we find

(114) $$g+g_{n}z=u-Ts+\frac{p}{\rho }+g_{n}z=-T\Lambda_{\rho }\;,$$

where *g* is the Gibbs free energy, and $g_{n}$ is gravitational acceleration. Thus, the sum of specific Gibbs free energy and specific potential energy, $g+g_{n}z$, is homogeneous in equilibrium, while density and pressure might be inhomogeneous.

In summary, maximizing entropy in the isolated system yields that the system is fully at rest, V=0, has homogeneous temperature, $T=1/\Lambda_{E}$, and, in the gravitational field, has inhomogeneous density and pressure, given implicitly by $g\left(T,\rho \right)+g_{n}z=-T\Lambda_{\rho }$. The Lagrange multipliers must be determined from the constraints (101), their values depend on the size and geometry of the system.

Equilibrium states of systems in contact with the environment, for example, with prescribed boundary temperatures or pressures are determined similarly, this includes systems with several phases [8].

To gain insight into the influence of potential energy, we evaluate (114) for ideal gases and incompressible fluids. For an ideal gas, the Gibbs free energy is $g\left(\rho ,T\right)\;=\;h\left(T\right)-T\left(\int_{T_{0}}^{T}\frac{c_{v}\left(T^{\prime }\right)}{T^{\prime }}dT^{\prime }-R\ln\frac{\rho }{\rho_{0}}+s_{0}\right)$. Using this in (114) and solving for density gives the barometric formula,

(115) $$\rho =\rho^{0}\exp\left[-\frac{g_{n}z}{RT}\right]\;,$$

where $\rho^{0}=\rho_{0}\exp\left[-\frac{\Lambda_{\rho }}{R}-\frac{h\left(T\right)-T\left(\int_{T_{0}}^{T}\frac{c_{v}\left(T^{\prime }\right)}{T^{\prime }}dT^{\prime }+s_{0}\right)}{RT}\right]$ is the density at reference height z=0. The ideal gas law gives the corresponding expression for pressure as

(116) $$p=p^{0}\exp\left[-\frac{g_{n}z}{RT}\right]\;,$$

where $p^{0}=\rho^{0}RT$ is the pressure at z=0. This is the well known barometric formula [8]. Pressure variation of gases in the gravitational field is relatively small. In technical systems with a size of few metres, the variation is so small that one can assume homogeneous pressures. When climbing a mountain (say), the pressure variation is important, of course.

For incompressible fluids, ρ=const., and internal energy and entropy depend only on temperature, so that the Gibbs free energy is $g\left(T,p\right)=u\left(T\right)+\frac{p}{\rho }-Ts\left(T\right)$. Using this in (114) and solving for pressure gives the hydrostatic pressure formula,

(117) $$p=p^{0}-\rho g_{n}z\;,$$

where $p^{0}=\rho T\left[s\left(T\right)-u\left(T\right)/T-\Lambda_{\rho }\right]$ is the pressure at reference height z=0. This is the well known hydrostatic pressure [8].

## 41. Thermodynamic Stability

The equilibrium state must be stable, which means that, indeed, it must be the maximum of the integral Φ (103). This requires that the second variation of Φ must be negative. In our case, where the integrand *X* depends only on *y*, this requires negative eigenvalues for the matrix of second derivatives $\partial^{2}X/\partial y^{2}$ at the location of the maximum. With the help of the Gibbs equation, the second derivatives can be written as

(118) $$\begin{array}{ccc} \frac{\partial X}{\partial \rho^{2}} & = & \left[\frac{1}{T}-\Lambda_{E}\right]\left[2{\left(\frac{\partial u}{\partial \rho }\right)}_{T}+\rho {\left(\frac{\partial^{2}u}{\partial \rho^{2}}\right)}_{T}\right]-\frac{1}{\rho T}{\left(\frac{\partial p}{\partial \rho }\right)}_{T}\;,\\ \frac{\partial^{2}X}{\partial {\vec{V}}^{2}} & = & -\rho \Lambda_{E}\;,\\ \frac{\partial^{2}X}{\partial T^{2}} & = & \rho \left[\frac{1}{T}-\Lambda_{E}\right]{\left(\frac{\partial^{2}u}{\partial T^{2}}\right)}_{\rho }-\frac{\rho }{T^{2}}{\left(\frac{\partial u}{\partial T}\right)}_{\rho }\;,\\ \frac{\partial^{2}X}{\partial \rho \partial T} & = & \frac{\partial^{2}X}{\partial T\partial \rho }=\left[\frac{1}{T}-\Lambda_{E}\right]{\left(\frac{\partial u}{\partial T}\right)}_{\rho }\;,\\ \frac{\partial X}{\partial \rho \partial \vec{V}} & = & \frac{\partial X}{\partial \vec{V}\partial \rho }=-{\vec{\Lambda }}_{M}-\Lambda_{E}\vec{V}\;,\\ \frac{\partial X}{\partial T\partial \vec{V}} & = & \frac{\partial X}{\partial \vec{V}\partial T}=0\;. \end{array}$$

These must now be evaluated at the equilibrium state, $T=1/\Lambda_{E}$ and $\vec{V}=-{\vec{\Lambda }}_{M}/\Lambda_{E}$. All mixed derivatives vanish in equilibrium, hence the requirement reduces to negative values for the diagonal elements. With the definitions of isothermal compressibility $\kappa_{T}=-\frac{1}{v}{\left(\frac{\partial v}{\partial p}\right)}_{T}$ and the specific heat at constant volume $c_{v}={\left(\frac{\partial u}{\partial T}\right)}_{v}$, the resulting conditions read

(119) $$\begin{array}{ccc} {\frac{\partial X}{\partial \rho^{2}}}_{|eq} & = & -\frac{1}{\rho T}{\left(\frac{\partial p}{\partial \rho }\right)}_{T}=-\frac{1}{\rho^{2}T\kappa_{T}}<0\;,\\ {\frac{\partial^{2}X}{\partial {\vec{V}}^{2}}}_{|eq} & = & -\frac{\rho }{T}<0\;,\\ {\frac{\partial^{2}X}{\partial T^{2}}}_{|eq} & = & -\frac{\rho }{T^{2}}{\left(\frac{\partial u}{\partial T}\right)}_{\rho }=-\frac{\rho }{T^{2}}c_{v}<0\;; \end{array}$$

With the mass density being positive, thermodynamic stability thus requires that isothermal compressibility, specific heat, and thermodynamic temperature are positive,

(120) $$\kappa_{T}>0\;\;\;,\;\;\;c_{v}>0\;\;\;,\;\;\;T>0\;.$$

These conditions imply that the volume decreases when pressure is increased isothermally, and that the temperature rises when heat is added to the system. Once more we see that positivity of thermodynamic temperature guarantees dissipation of kinetic energy. The stability conditions (120) imply that $s\left(u,v\right)$ is a concave function, as shown in Appendix B.

## 42. Open Systems

**Figure 15:**
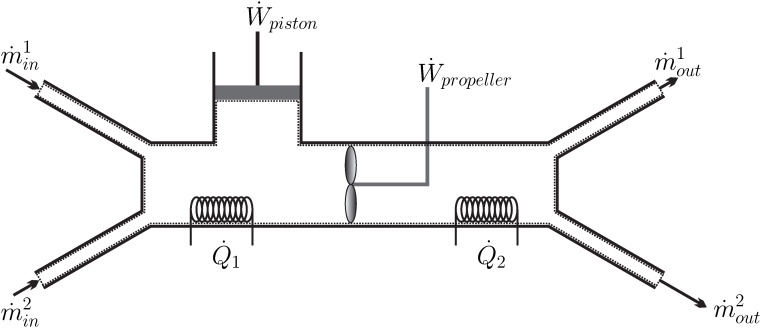
Open system with two inflows, two outflows and two heat sources. The dotted line indicates the system boundary.

So far we have considered only closed systems, which do not exchange mass. We shall now extend the discussion to systems which exchange mass with their surroundings. Figure 15 shows a generic *open system* with two inflows and two outflows. The amount of mass exchanged per unit time, the mass transfer rate or *mass flow*, is denoted by $\dot{m}$. The system also exchanges propeller and piston work, $\dot{W}={\dot{W}}_{propeller}+{\dot{W}}_{piston}$, and heat, $\dot{Q}={\dot{Q}}_{1}+{\dot{Q}}_{2}$, with its surroundings, just as a closed system does.

States in open systems are normally inhomogeneous. One might think of a mass element entering the system of Figure 15 on the left. As the element travels through the system, it constantly changes its state: When it passes the heating, its temperature changes, when it passes the propeller its pressure and temperature change, and so on. Thus, at each location within the system one finds different properties. As discussed earlier, an inhomogeneous system is in a nonequilibrium state. In an open system the nonequilibrium is maintained through the exchange of mass, heat and work with the surroundings.

The equations for open systems presented below are those typically found in thermodynamics textbooks. These rely on a number of simplifying assumptions, such as use of average values for properties and ignore of viscous stresses across inlets and exits, which will only be mentioned, but will not be discussed in detail [8].

## 43. Balances of Mass, Energy and Entropy

Mass cannot be created or destroyed, that is mass is conserved. Chemical reactions change the composition of the material, but not its mass. In a closed system, the law of mass conservation states that the total mass *m* in the system does not change in time, that is, it simply reads $\frac{dm}{dt}=0$. In an open system, where mass enters or leaves over the system boundaries, the conservation law for mass states that the change of mass in time is due to inflow—which increases mass—and outflow—which decreases system mass. In approximation, the mass flow can be written as

(121) $$\dot{m}=\rho \;V\;A\;,$$

where ρ and V are averages of mass density and velocity over the cross section *A* of the in/outflow boundary.

The rate of change in mass is due to the net difference of mass flows entering and leaving the system,

(122) $$\frac{dm}{dt}=\sum_{\mathrm{in}}{\dot{m}}_{i}-\sum_{\mathrm{out}}{\dot{m}}_{e}\;.$$

The indices $\left(i,e\right)$ indicate the values of the properties at the location where the respective flows cross the system boundary, that is their average values at the inlets and outlets, respectively.

The total energy *E* of an open system changes due to exchange of heat and work, and due to *convective energy transport* $\dot{E}$, that is energy carried in or out by the mass crossing the system boundary,

(123) $$\dot{E}=\dot{m}e=\dot{m}\left(u+\frac{1}{2}V^{2}+g_{n}z\right)\;.$$

The power required to push mass over the system boundary is the force required times the velocity. The force is the local pressure (irreversible stresses are ignored) times the cross section, thus the associated *flow work* is

(124) $${\dot{W}}_{flow}=-\left(pA\right)V=-\frac{p}{\rho }\dot{m}\;.$$

Work is done to the system when mass is entering, then ${\dot{W}}_{flow}$ must be negative. The system does work to push leaving mass out, then ${\dot{W}}_{flow}$ must be positive. Accordingly, flow work points opposite to mass flow, which is ensured by the minus sign in the equation.

Thus, in comparison to the energy balance for closed systems, the energy balance for the general open system of Figure 15 has additional contributions to account for convective energy transport and flow work, in condensed notation

(125) $$\frac{dE}{dt}=\dot{Q}-\dot{W}+\sum_{\mathrm{in}/\mathrm{out}}\dot{E}-\sum_{\mathrm{in}/\mathrm{out}}{\dot{W}}^{flow}\;,$$

where the sums have to be taken over all flows crossing the system boundary.

Explicitly accounting for mass flows leaving and entering the system, and with enthalpy h=u+p/ρ, the 1st law—the balance of energy—for the general open system becomes

(126) $$\frac{dE}{dt}=\sum_{\mathrm{in}}{\dot{m}}_{i}{\left(h+\frac{1}{2}V^{2}+g_{n}z\right)}_{i}-\sum_{\mathrm{out}}{\dot{m}}_{e}{\left(h+\frac{1}{2}V^{2}+g_{n}z\right)}_{e}+\dot{Q}-\dot{W}\;.$$

This equation states that the energy *E* within the system changes due to convective inflow and outflow, as well as due to heat transfer and work. Note that the flow energy includes the flow work required to move the mass across the boundaries (124). Moreover, there can be several contributions to work and heat transfer, that is $\dot{W}=\sum_{j}{\dot{W}}_{j}$ and $\dot{Q}=\sum_{k}{\dot{Q}}_{k}$.

All mass that is entering or leaving the system carries entropy. The entropy flow associated with a mass flow is simply $\dot{S}=\dot{m}s$, where *s* is the average specific entropy at the respective inlet or outlet. Adding the appropriate terms for inflow and outflow to the 2nd law (69) for closed systems yields the 2nd law—the balance of entropy—for open systems as

(127) $$\frac{dS}{dt}=\sum_{\mathrm{in}}{\dot{m}}_{i}s_{i}-\sum_{\mathrm{out}}{\dot{m}}_{e}s_{e}+\sum_{k}\frac{{\dot{Q}}_{k}}{T_{k}}+{\dot{S}}_{gen}\;\;\;\mathrm{with}\;\;\;{\dot{S}}_{gen}\geq 0\;.$$

This equation states that the entropy *S* within the system changes due to convective inflow and outflow, as well as due to entropy transfer caused by heat transfer (${\dot{Q}}_{k}/T_{k}$) and entropy generation due to irreversible processes inside the system (${\dot{S}}_{gen}\geq 0$). If all processes within the system are reversible, the entropy generation vanishes (${\dot{S}}_{gen}=0)$. Recall that ${\dot{Q}}_{k}$ is the heat that crosses the system boundary where the boundary temperature is $T_{k}$.

## 44. One Inlet, One Exit Systems

**Figure 16:**
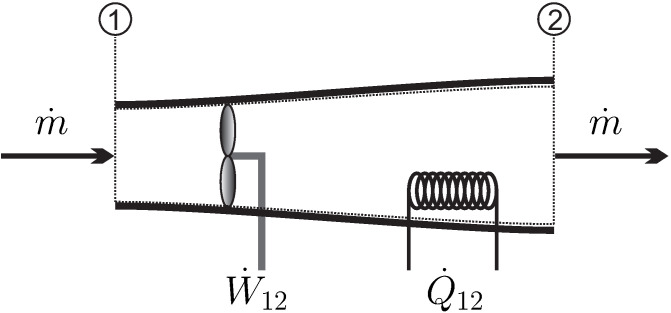
Typical one-inlet-one-exit system.

A case of particular interest are steady-state systems with only one inlet and one exit, as sketched in Figure 16, for which the mass balance reduces to

(128) $${\dot{m}}_{in}={\dot{m}}_{out}=\dot{m}\;.$$

There is just one constant mass flow $\dot{m}$ flowing through each cross section of the system.

For a steady state system, the corresponding forms for energy and entropy balance are

(129) $$\dot{m}\left[h_{2}-h_{1}+\frac{1}{2}\left(V_{2}^{2}-V_{1}^{2}\right)+g_{n}\left(z_{2}-z_{1}\right)\right]={\dot{Q}}_{12}-{\dot{W}}_{12}\;,$$

(130) $$\dot{m}\left(s_{2}-s_{1}\right)-\sum_{k}\frac{{\dot{Q}}_{k}}{T_{k}}={\dot{S}}_{gen}\geq 0\;.$$

It is instructive to study the equations for an infinitesimal step within the system, that is, for infinitesimal system length dx, where the differences reduce to differentials,

(131) $$\begin{array}{ccc} \dot{m}\left(dh+\frac{1}{2}dV^{2}+g_{n}dz\right) & = & \delta \dot{Q}-\delta \dot{W}\;, \end{array}$$

(132) $$\begin{array}{ccc} \dot{m}ds-\frac{\delta \dot{Q}}{T} & = & \delta {\dot{S}}_{gen}\;. \end{array}$$

Heat and power exchanged, and entropy generated, in an infinitesimal step along the system are process dependent, and as always we write ($\delta \dot{Q},\delta \dot{W},\delta \dot{S}$) to indicate that these quantities are not exact differentials. Use of the Gibbs equation in the form Tds=dh−vdp allows to eliminate dh and $\delta \dot{Q}$ between the two equations to give an expression for power,

(133) $$\delta \dot{W}=-\dot{m}\left(vdp+\frac{1}{2}dV^{2}+g_{n}dz\right)-T\delta {\dot{S}}_{gen}\;.$$

The total power for the finite system follows from integration over the length of the system as

(134) $${\dot{W}}_{12}=-\dot{m}\int_{1}^{2}\left(vdp+\frac{1}{2}dV^{2}+g_{n}dz\right)-\int_{1}^{2}T\delta {\dot{S}}_{gen}\;.$$

Since $T\delta {\dot{S}}_{gen}\geq 0$, we see—again—that irreversibilities reduce the power output of a power producing device (where ${\dot{W}}_{12}>0$), and increase the power demand of a power consuming device (where ${\dot{W}}_{12}<0$). Efficient energy conversion requires to reduce irreversibilities as much as possible.

When we consider (133) for a flow without work, we find Bernoulli’s equation (Daniel Bernoulli, 1700–1782) for pipe flows as

(135) $$vdp+\frac{1}{2}dV^{2}+g_{n}dz=-\frac{1}{\dot{m}}T\delta {\dot{S}}_{gen}\;.$$

The Bernoulli equation is probably easier to recognize in its integrated form for incompressible fluids (where $v=\frac{1}{\rho }=const.),$

(136) $$g_{n}\left(H_{2}-H_{1}\right)=\frac{p_{2}-p_{1}}{\rho }+\frac{1}{2}\left(V_{2}^{2}-V_{1}^{2}\right)+g_{n}\left(z_{2}-z_{1}\right)=-\frac{1}{\dot{m}}\int_{1}^{2}T\delta {\dot{S}}_{gen}\;.$$

Here, $H=\frac{p}{\rho g_{n}}+\frac{1}{2}\frac{V^{2}}{g_{n}}+z$ denotes hydraulic head. The right hand side describes loss of hydraulic head due to irreversible processes, in particular friction.

Finally, for reversible processes—where $\delta {\dot{S}}_{gen}=0$ in (134)—we find the *reversible steady-flow work*

(137) $${\dot{W}}_{12}^{rev}=-\dot{m}\int_{1}^{2}\left(vdp+\frac{1}{2}dV^{2}+g_{n}dz\right)\;.$$

For flows at relatively low velocities and without significant change of level the above relation can be simplified to

(138) $${\dot{W}}_{12}^{rev}=\dot{m}w_{12}^{rev}=-\dot{m}\int_{1}^{2}vdp\;.$$

In a p-v-diagram, the *specific reversible flow work* $w_{12}^{rev}$ is the area to the left of the process curve.

The heat exchanged in a reversible process in a steady-state, one inlet, one exit system follows from the integration of the second law (132) with $\delta {\dot{S}}_{gen}=0$ as

(139) $${\dot{Q}}_{12}^{rev}=\int_{1}^{2}\delta {\dot{Q}}^{rev}=\dot{m}\int_{1}^{2}Tds=\dot{m}q_{12}^{rev}\;.$$

In a T-s-diagram, $q_{12}^{rev}$ is the area below the process curve, just as in a closed system.

## 45. Entropy Generation in Mass Transfer

Friction in flows leads to loss of pressure and corresponding entropy generation. When we consider a simple flow with no work added or withdrawn, Equation (135) gives the entropy generated in dx as

(140) $$\delta {\dot{S}}_{gen}=-\frac{\dot{m}}{T}\left(vdp+\frac{1}{2}dV^{2}+g_{n}dz\right)\;.$$

The total entropy generated in a finite system is

(141) $${\dot{S}}_{gen}=-\dot{m}\int_{in}^{out}\frac{1}{T}\left(vdp+\frac{1}{2}dV^{2}+g_{n}dz\right)\;.$$

For a system where kinetic and potential energy are unimportant, this reduces to

(142) $${\dot{S}}_{gen}=-\dot{m}\int_{in}^{out}\frac{v}{T}dp\;.$$

Once more, we interpret the entropy generation rate as the product of a flux, the mass flow $\dot{m}$, and a thermodynamic force, namely the integral over $-\frac{v}{T}dp$. Here, pressure in equilibrium is homogeneous, since gravitation is ignored. Deviation from homogeneous pressure is the thermodynamic force that induces a mass flow to equilibrate pressure.

Since specific volume *v* and thermodynamic temperature *T* are strictly positive, the force is proportional to the pressure difference, $-\int_{in}^{out}\frac{v}{T}dp\propto \left(p_{in}-p_{out}\right)$. In order to obtain a positive entropy generation rate, linear irreversible thermodynamics suggests that the mass flow be proportional to the force, which is the case for

(143) $$\dot{m}=\zeta A\left(p_{in}-p_{out}\right)=\zeta A\Delta p\;.$$

Here, *A* is the mass transfer area and ζ>0 is a positive transport coefficient that must be measured.

One particular example for this law is the Hagen-Poiseuille relation (Gotthilf Hagen, 1797–1884; Jean Poiseuille, 1797–1869) of fluid dynamics which gives the volume flow $\dot{V}=\dot{m}/\rho$ of a fluid with shear viscosity μ through a pipe of radius *R* and length *L* as

(144) $$\dot{V}=\frac{\pi R^{4}}{8\mu L}\Delta p\;.$$

Another example for (143) is Darcy’s law (Henry Darcy, 1803-1858) that describes flow through porous media. Then *A* is the cross section of the porous medium considered, and ζ is a coefficient of permeability.

Real processes are irreversible, and produce entropy. For a simple flow, the work loss to irreversibilities is

(145) $${\dot{W}}_{\mathrm{loss}}=\int_{in}^{out}T\delta {\dot{S}}_{gen}\;.$$

Since $\delta {\dot{S}}_{gen}=-\frac{\dot{m}}{T}vdp$, for isothermal flow of an incompressible liquid, entropy generation and work loss are

(146) $${\dot{S}}_{gen}=\frac{\dot{V}}{T}\left(p_{in}-p_{out}\right)\;\;,\;\;\;{\dot{W}}_{\mathrm{loss}}=\dot{V}\left(p_{in}-p_{out}\right)\;,$$

where $\dot{V}=\dot{m}v$ is the volume flow.

For an ideal gas flow, we have instead

(147) $${\dot{S}}_{gen}=\dot{m}R\ln\frac{p_{in}}{p_{out}}\;\;\;,\;\;\;{\dot{W}}_{\mathrm{loss}}=-\dot{m}\int_{1}^{2}vdp\;.$$

## 46. Global and Local Thermodynamics

From the very beginning of the discussion, we have emphasized entropy as a property describing equilibrium *and* nonequilibrium states. In that, it does not differ from other properties, such as mass density, momentum, or energy, which all are well defined in nonequilibrium. Nonequilibrium states typically are inhomogeneous, hence all properties must be defined locally, for example, as specific properties for the volume element dV.

Most thermodynamic systems, and certainly those of engineering interest, involve inhomogeneous states. While a lot can be learned about these systems by considering the thermodynamic laws for the system, for example, the global laws (122), (126) and (127), a complete understanding of a thermodynamic system requires the look inside, that is the complete resolution of local properties at all times, such as mass density $\rho \left(\vec{r},t\right)$, velocity $\vec{V}\left(\vec{r},t\right)$, temperature $T\left(\vec{r},t\right)$. In transport theories such as *Fluid Dynamics* or *Heat Transfer* the local conservation laws are solved, their numerical solution is known as *Computational Fluid Dynamics* (CFD).

The topic of *Nonequilibrium Thermodynamics* is to identify the transport equations needed, and the constitutive equations required for their closure.

The Navier-Stokes-Fourier equations of classical thermo- and fluid dynamics are derived in the theory of *Linear Irreversible Thermodynamics* (LIT), which relies on the assumption of local thermodynamic equilibrium. A short outline of LIT is presented in Appendix C. The method combines the Gibbs equation with local conservation laws to find the balance law for entropy. Constitutive equations for stress tensor and heat flux are constructed such that the local entropy generation is always positive. Thus, the method provides not only transport equations, but also the accompanying 2nd law. The global laws as formulated above (122), (126) and (127) result from integration over system volume (with some simplifying assumptions).

Appendix C.4 also has a short discussion on boundary conditions for fluids and gases, where evaluation of the 2nd law suggests temperature jumps and velocity slip at the interface between a fluid and a wall.

Systems in local thermodynamic equilibrium, but global nonequilibrium, are relatively easy to describe, since the thermal and caloric equations of state, and the Gibbs equation, which are well-known from equilibrium thermodynamics, remain valid locally. Considering that equilibrium is approached over time, one will expect that local equilibrium states will be observed when the changes in the system, for example, the manipulation at system boundaries, are sufficiently slow, and gradients are sufficiently flat.

For systems with fast changes, and steep gradients, one will *not* encounter local equilibrium states, hence the equilibrium property relations cannot be used for their description. The question of determining the local property relations, and the transport equations, for systems in strong nonequilibrium is the subject of modern nonequilibrium thermodynamics. The answers depend on the school, and the material considered—a rarefied gas behaves differently from a visco-elastic fluid. Overarching frameworks are available, such as Extended Thermodynamics [20,21], or GENERIC (general equation for the nonequilibrium reversible-irreversible coupling) [22]. These include the equations of classical linear irreversible thermodynamics as proper limits, but go well beyond these. They typically include nonequilibrium property relations, often use an extended set of variables to describe the nonequilibrium state, and invariably include a formulation of the 2nd law, with nonequilibrium relations for entropy and its flux. Here is not the point to discuss this further, the cited books are good starting points for further inquiry.

## 47. Kinetic Theory of Gases

Instructive insights into the 2nd law can be found in Boltzmann’s *Kinetic Theory of Gases*, which describes a gas as an ensemble of particles that move in space, and collide among themselves and with walls. An abridged and simplified overview of some aspects of the theory for ideal monatomic gases is presented in Appendix D. The Boltzmann equation describes the time-space evolution of the gas towards an equilibrium state from the microscopic viewpoint. The macroscopic conservation laws for mass, momentum and energy are obtained from suitable averages of the Boltzmann equation. The equations for local thermodynamic equilibrium can be obtained from suitable limits (small Knudsen number), in full agreement with LIT.

Boltzmann’s celebrated H-theorem identifies a macroscopic quantity which has all properties of entropy, and, in our opinion is entropy, indeed. In particular, the H-function obeys a balance law with non-negative production, and in equilibrium it reduces to the equilibrium entropy of an ideal gas. The Boltzmann entropy is defined for arbitrary states, including *all* inhomogeneous nonequilibrium states far from local equilibrium, in agreement with our assumption on entropy throughout.

The underlying microscopic picture provides an interpretation for entropy, a quantity that arose first from purely phenomenological considerations. This interpretation is the topic of the next sections.

## 48. What is Entropy?

The arguments that gave us the second law and entropy as a property centered around the trend to equilibrium observed in any system left to itself (isolated system). Based on the derivation, the question *What is entropy?* can be answered simply by saying *it is a quantity that arises when one constructs an inequality that describes the trend to equilibrium.* Can there be a deeper understanding of entropy?

Before we try to answer, we look at internal energy—when the first law of thermodynamics was found, the concept of internal energy was new, and it was difficult to understand what it might describe. At that time, the atomic structure of matter was not known, and internal energy could not be interpreted—it appeared because it served well to describe the phenomena. Today we know more, and we understand internal energy as the kinetic, potential, and quantum energies of atoms and molecules on the microscopic level. Thus, while the concept of internal energy arose from the desire to describe phenomena, today it is relatively easy to understand, because it has a macroscopic analogue in mechanics.

Entropy also came into play to describe the phenomena, but it is a new quantity, without a mechanical analogue. A deeper understanding of entropy can be gained, as for internal energy, from considerations on the atomic scale. Within the framework of his *Kinetic Theory of Gases*, Ludwig Boltzmann (1844–1905) found the microscopic interpretation of entropy (see Appendix D).

Macroscopically, a state is described by only a few macroscopic properties, for example, temperature, pressure, volume. Microscopically, a state is described through the location and momentum of all atoms within the system. The microscopic state is constantly changing due to the microscopic motion of the atoms, and there are many microscopic states that describe the same macroscopic state. If we denote the total number of all microscopic states that describe the same macroscopic state by Ω, then the entropy of the macroscopic state according to Boltzmann is

(148) $$S=k_{B}\ln\Omega \;.$$

The constant $k_{B}=\bar{R}/A=1.3804\times 10^{-23}\frac{J}{K}$ is the Boltzmann constant, which can be interpreted as the gas constant per particle; $A=6.022\times 10^{23}\frac{1}{\mathrm{mol}}$ is the Avogadro constant.

The growth of entropy in an isolated system, $\frac{dS}{dt}\geq 0$, thus means that the system shifts to macrostates which have larger numbers of microscopic realizations. As we will see, equilibrium states have particularly large numbers of realizations, and this is why they are observed.

## 49. Ideal Gas Entropy

To make the ideas somewhat clearer, we consider the expansion of a gas when a barrier is removed, see Section 35. This is a particularly simple case, where the internal energy, and thus the distribution of energy over the particles, does not change. Hence, we can ignore the distribution of thermal energy over the particles, and the exchange of energy between them.

We assume a system of *N* gas particles in a volume *V*. The volume of a single particle is $v_{0}$, and in order to be able to compute the number Ω, we “quantize” the accessible volume *V* into $n=V/v_{0}$ boxes that each can accommodate just one particle. Note that in a gas most boxes are empty. Due to their thermal energy, the atoms move from box to box. The number of microstates is simply given by the number of realizations of a state with *N* filled boxes and $\left(n-N\right)$ empty boxes, which is

(149) $$\Omega \left(N,V\right)=\frac{n!}{N!\left(n-N\right)!}\;.$$

By means of Stirling’s formula lnx!=xlnx−x (for x≫1) the entropy (148) for this state becomes

(150) $$S\left(N,V\right)=k_{B}\left[-N\ln\frac{N}{n}-\left(n-N\right)\ln\left(1-\frac{N}{n}\right)\right]\;.$$

Now we can compute the change of entropy with volume. For this, we consider the same *N* particles in two different volumes, $V_{1}=n_{1}v_{0}$ and $V_{2}=n_{2}v_{0}$. The entropy difference $S_{2}-S_{1}=S\left(N,V_{2}\right)-S\left(N,V_{1}\right)$ between the two states is

(151) $$S_{2}-S_{1}=k_{B}\left[N\ln\frac{n_{2}}{n_{1}}+n_{1}\ln\left(1-\frac{N}{n_{1}}\right)-n_{2}\ln\left(1-\frac{N}{n_{2}}\right)+N\ln\frac{\left(1-\frac{N}{n_{2}}\right)}{\left(1-\frac{N}{n_{1}}\right)}\right]\;.$$

In an ideal gas the number of possible positions *n* is much bigger than the number of particles *N*, that is $\frac{N}{n_{1}}\ll 1,\frac{N}{n_{2}}\ll 1$. Taylor expansion yields the entropy difference to leading order as

(152) $$S_{2}-S_{1}=k_{B}N\ln\frac{n_{2}}{n_{1}}=mR\ln\frac{V_{2}}{V_{1}}\;,$$

where we reintroduced volume ($V_{1,2}=n_{1,2}v_{0}$), and introduced the mass as m=MN/A; $R=\bar{R}/M$ is the gas constant. This is just the change of entropy computed in Section 35.

## 50. Homogeneous vs. Inhomogeneous States

It is instructive to compare the number of realizations for the two cases, for which we find

(153) $$\frac{\Omega_{2}}{\Omega_{1}}=\exp\frac{S_{2}-S_{1}}{k}=\exp\left(N\ln\frac{V_{2}}{V_{1}}\right)={\left(\frac{V_{2}}{V_{1}}\right)}^{N}\;.$$

For a macroscopic amount of gas, the particle number *N* is extremely large (order of magnitude ∼$10^{23}$), so that already for a small difference in volume the ratio of microscopic realization numbers is enormous. For instance for $V_{2}=2V_{1}$, we find $\frac{\Omega_{2}}{\Omega_{1}}=2^{N}$.

Microscopic states change constantly due to travel of, and collisions between, particles. Each of the Ω microstates compatible with the given macrostate is observed with the same probability, 1/Ω. The $\Omega_{1}$ microstates in which the gas is confined in the volume $V_{1}$ are included in the $\Omega_{2}$ microstates in which the gas is confined in the larger volume $V_{2}$. Thus, after removal of the barrier, there is a finite, but extremely small probability of $P=\frac{\Omega_{1}}{\Omega_{2}}={\left(\frac{V_{1}}{V_{2}}\right)}^{N}$ to find all gas particles in the initial volume $V_{1}$. This probability is so small that the expected waiting time for observing a return into the original volume exceeds the lifetime of the universe by many orders of magnitude. If we do not want to wait that long for the return to initial state, we have to push the gas back into the initial volume, which requires work.

In generalization of the above, we can conclude that it is quite unlikely that a portion $V_{\nu }$ of the volume is void of particles. The corresponding probability is $P_{\nu }={\left(\frac{V-V_{\nu }}{V}\right)}^{N}$. The average volume available for one particle is $\bar{V}=\frac{V}{N}$, and when $V_{\nu }=\nu \bar{V}$ we find, for the large particle numbers in an macroscopic amount of gas, $P_{\nu }={\left(1-\frac{\nu }{N}\right)}^{N}≃e^{-\nu }$. Thus, as long as $V_{\nu }$ is bigger than the average volume for a single particle, so that ν>1, the probability for a void is very small. Moreover, strongly inhomogeneous distributions are rather unlikely, since the number of homogeneous distributions is far larger in number. Hence, we observe homogeneous distributions in equilibrium.

A closer look at equilibrium properties reveals small local fluctuations of properties, for example, mass density, which are induced by the thermal motion of particles. The equilibrium state is stable, that is these random disturbances decay in time, so that in average the equilibrium state is observed. For macroscopic systems the fluctuations are so small that they can be ignored. Nevertheless, fluctuations in density lead to light scattering, which can be used to determine transport coefficients such as viscosity and heat conductivity from equilibrium states [20]. Since blue light is more likely to be scattered in density fluctuations of the atmosphere, the sky appears blue.

## 51. Entropy and Disorder

Often it is said that entropy is a measure for disorder, where disorder has a higher entropy. One has to be rather careful with this statement, since order, or disorder, are not well-defined concepts. To shed some light on this, we use the following analogy—the ordered state of an office is the state where all papers, folders, books and pens are in their designated shelf space. Thus, they are confined to a relatively small initial volume of the shelf, $V_{1}$. When work is done in the office, all these papers, folders, books and pens are removed from their initial location, and, after they are used, are dropped somewhere in the office—now they are only confined to the large volume of the office, $V_{2}$. The actions of the person working in the office constantly change the microstate of the office (the precise location of that pen … where is it now?), in analogy to thermal motion.

At the end of the day, the office looks like a mess and needs work to clean up. Note, however, that the final state of the office—which appears to be so disorderly—is just *one* accessible microstate, and therefore it has the same probability as the fully ordered state, where each book and folder is at its designated place on the shelf. A single microstate, for example, a particular distribution of office material over the office in the evening, has no entropy. Entropy is a macroscopic property that counts the number of all possible microstates, for example, all possible distributions of office material.

A macroscopic state which puts strong restrictions on the elements has a low entropy, for example, when all office material is in shelves behind locked doors. When the restrictions are removed—the doors are unlocked—the number of possible distributions grows, and so does entropy. Thermal motion leads to a constant change of the distribution within the inherent restrictions.

To our eye more restricted macroscopic states—all gas particles only in a small part of the container, or all office material behind closed doors—appear more orderly, while less restricted states generally appear more disorderly. Only in this sense one can say that entropy is a measure for disorder.

In the office, every evening the disordered state differs from that of the previous day. Over time, one faces a multitude of disordered states, that is the disordered office has many realizations, and a large entropy. In the end, this makes cleaning up cumbersome, and time consuming.

Our discussion focussed on spatial distributions where the notion of order is well-aligned with our experience. The thermal contribution to entropy is related to the distribution of microscopic energy $e_{m}$ over the particles, where $e_{m}$ is the microscopic energy per particle. In *Statistical Thermodynamics* one finds that in equilibrium states the distribution of microscopic energies between particles is exponential, $A\exp\left[-\frac{e_{m}}{kT}\right]$. The factor *A* must be chosen such that the sum over all particles gives the internal energy, $U=\sum_{m}Ae_{m}\exp\left[-\frac{e_{m}}{k_{B}T}\right]$. One might say that the exponential itself is an orderly function, so that the equilibrium states are less disordered than nonequilibrium states. Moreover, for lower temperatures the exponential is more narrow, the microscopic particle energies are confined to lower values, and one might say that low temperature equilibrium states are more orderly than high temperature equilibrium states. And indeed, we find that entropy grows with temperature, that is colder systems have lower entropies.

## 52. Summary

Looking back at the above, it is clear that we have not established any new thermodynamics, but provided our perspective on entropy and the 2nd law. Throughout the discussion entropy is established as a property for any state, be it in equilibrium and nonequilibrium. While this is standard in all theories on nonequilibrium thermodynamics, and in kinetic theory of gases, one finds many discussions of thermodynamics that define entropy only for equilibrium states. Restriction of entropy to equilibrium state is an unnecessary assumption, that reduces applicability of thermodynamics, but can easily be avoided.

Engineering applications of thermodynamics invariably have to account for inhomogeneous nonequilibrium states, hence a clear description of entropy as nonequilibrium quantity is required, and is, indeed, used.The global balance laws used in engineering textbooks follow from the assumption of local thermodynamic equilibrium, which allows to use the equilibrium property relations locally. The same assumption gives the Navier-Stokes-Fourier equations, that is, the partial differential equations describing all local process details. Global and local descriptions are equivalent.

Entropy, and all other properties such as density, energy, and so forth, are also meaningful for extreme nonequilibrium states, which are not in local equilibrium. The associated property relations and transport equations might differ considerably from those based on the local equilibrium hypothesis, but this does not imply that energy or entropy lose their meaning.

Positivity of thermodynamic temperature guarantees dissipation of work and kinetic energy. This is best seen in the stability analysis, where motion of volume elements, that is, kinetic energy, is included. In the frame where system momentum vanishes, the local velocity will vanish in equilibrium, and this equilibrium resting state is stable only if thermodynamic temperature is positive.

Entropy and the 2nd law were introduced based on five intuitive observations that are in agreement with daily experience. There is no need of any discussion of thermodynamic cycles and engines to introduce entropy and the 2nd law. This greatly simplifies access to the subject—both for teaching and studying thermodynamics—since all thermodynamic cycles and engines are discussed only after the laws of thermodynamics are established.

For teaching thermodynamics, I use a variant—with some shortcuts—of the approach developed here [8]. This allows fast and meaningful access to the thermodynamic laws as early in the course as possible, so that all applications can rely on 1st and 2nd law from the beginning.

## Appendix A. Caratheodory’s Approach

Our approach towards entropy and the 2nd law is based on the observation of phenomena. Attempts for building thermodynamics axiomatically were presented by C. Caratheodory [9,19], and more recently by Lieb and Yngvason [12,13].

Restricting his attention to adiabatic processes and equilibrium states only, Caratheodory formulated the 2nd law in the statement that *in each neighbourhood of an arbitrary initial state there exist states that cannot be reached by adiabatic processes*. While initial and end states are equilibrium states, the processes that connect these states are allowed to be irreversible (work only). This statement is, of course, compatible with the 2nd law (44), which for adiabatic processes reduces to $\frac{dS}{dt}={\dot{S}}_{gen}\geq 0\;$—states with smaller entropy than the initial state cannot be reached adiabatically.

Using sophisticated reasoning within multivariable calculus, Caratheodory proceeds to find the Gibbs equation where thermodynamic temperature is identified as a material independent integrating factor.

Compared to our approach, entropy is defined only for equilibrium states and, since only adiabatic processes are considered, heat—and therefore entropy flux as heat over temperature—does not appear in the 1st and 2nd law. Therefore, the full entropy balance (44), which describes irreversible processes with heat and work interaction, cannot be found within this framework.

## Appendix B. Concavity of Equilibrium Entropy

We show that the stability conditions (120) are equivalent to the statement that equilibrium entropy $s\left(u,v\right)$ is a concave function of its arguments. The requirement for concavity is that the matrix of second derivatives is negative definite. A symmetric matrix $A=\left\{\begin{array}{cc} a & b\\ b & c \end{array}\right\}$ is negative definite if x·A·x<0 for arbitrary vectors $x=\left\{x_{1},x_{2}\right\}$. This is the case for a<0, $c-\frac{b^{2}}{a}<0$, which implies c<0. Hence, concavity of $s\left(u,v\right)$ is given for

(A1) $${\left(\frac{\partial^{2}s}{\partial u\partial u}\right)}_{v}<0\;\;\;,\;\;\;{\left(\frac{\partial^{2}s}{\partial u\partial u}\right)}_{v}-\frac{{\left(\frac{\partial^{2}s}{\partial v\partial u}\right)}^{2}}{{\left(\frac{\partial^{2}s}{\partial v\partial v}\right)}_{u}}<0\;.$$

The reformulation of these conditions into the stability conditions (120) is a beautiful application of multi-variable calculus.

The first derivatives of entropy are obtained from the Gibbs equation Tds=du+pdv as

(A2) $${\left(\frac{\partial s}{\partial u}\right)}_{v}=\frac{1}{T}\;\;\;,\;\;\;{\left(\frac{\partial s}{\partial v}\right)}_{u}=\frac{p}{T}\;.$$

From this we have, with the definition of specific heat (19),

(A3) $${\left(\frac{\partial^{2}s}{\partial u\partial u}\right)}_{v}={\left(\frac{\partial }{\partial u}\frac{1}{T}\right)}_{v}=-\frac{1}{T^{2}}{\left(\frac{\partial T}{\partial u}\right)}_{v}=-\frac{1}{T^{2}c_{v}}<0\;,$$

To bring the other second derivatives into a compact form requires repeated use of the identity ${\left(\frac{\partial x}{\partial z}\right)}_{y}=-{\left(\frac{\partial x}{\partial y}\right)}_{z}{\left(\frac{\partial y}{\partial z}\right)}_{x}$. For the mixed derivative we find

(A4) $$\frac{\partial^{2}s}{\partial v\partial u}={\left(\frac{\partial \left(\frac{1}{T}\right)}{\partial v}\right)}_{u}=-\frac{1}{T^{2}}{\left(\frac{\partial T}{\partial v}\right)}_{u}=-\frac{1}{T^{2}}\left[-{\left(\frac{\partial T}{\partial u}\right)}_{v}{\left(\frac{\partial u}{\partial v}\right)}_{T}\right]=\frac{1}{T^{2}c_{v}}{\left(\frac{\partial u}{\partial v}\right)}_{T}\;.$$

For the second volume derivative we find at first

(A5) $${\left(\frac{\partial^{2}s}{\partial v\partial v}\right)}_{u}={\left(\frac{\partial \left(\frac{p}{T}\right)}{\partial v}\right)}_{u}=-\frac{p}{T^{2}}{\left(\frac{\partial T}{\partial v}\right)}_{u}+\frac{1}{T}{\left(\frac{\partial p}{\partial v}\right)}_{u}=\frac{p}{c_{v}T^{2}}{\left(\frac{\partial u}{\partial v}\right)}_{T}-\frac{1}{T}{\left(\frac{\partial p}{\partial u}\right)}_{v}{\left(\frac{\partial u}{\partial v}\right)}_{p}\;.$$

For further simplification, we consider energy as a function of $\left(T,v\right)$, with the differential $du=c_{v}dT+{\left(\frac{\partial u}{\partial v}\right)}_{T}dv$, from which we find the partial derivatives

(A6) $${\left(\frac{\partial u}{\partial v}\right)}_{p}=c_{v}{\left(\frac{\partial T}{\partial v}\right)}_{p}+{\left(\frac{\partial u}{\partial v}\right)}_{T}\;\;\;,\;\;\;{\left(\frac{\partial u}{\partial p}\right)}_{v}=\frac{1}{{\left(\frac{\partial p}{\partial u}\right)}_{v}}=c_{v}{\left(\frac{\partial T}{\partial p}\right)}_{v}\;.$$

We use the above, and (54), to find

$$\begin{array}{ccc} {\left(\frac{\partial^{2}s}{\partial v\partial v}\right)}_{u} & = & \frac{1}{T^{2}c_{v}}\left[p-T{\left(\frac{\partial p}{\partial T}\right)}_{v}\right]{\left(\frac{\partial u}{\partial v}\right)}_{T}-\frac{1}{T}{\left(\frac{\partial T}{\partial v}\right)}_{p}{\left(\frac{\partial p}{\partial T}\right)}_{v}\\ & = & -\frac{1}{T^{2}c_{v}}{\left(\frac{\partial u}{\partial v}\right)}_{T}{\left(\frac{\partial u}{\partial v}\right)}_{T}+\frac{1}{T}{\left(\frac{\partial p}{\partial v}\right)}_{T}\;, \end{array}$$

so that

$${\left(\frac{\partial^{2}s}{\partial v\partial v}\right)}_{u}-\frac{{\left(\frac{\partial^{2}s}{\partial v\partial u}\right)}^{2}}{{\left(\frac{\partial^{2}s}{\partial u\partial u}\right)}_{v}}=\frac{1}{T}{\left(\frac{\partial p}{\partial v}\right)}_{T}=-\frac{1}{vT\kappa_{T}}<0\;.$$

Here, we have used the stability conditions (120), which state that T>0, $\kappa_{T}=-\frac{1}{v}{\left(\frac{\partial v}{\partial p}\right)}_{T}>0$. Hence concave equilibrium entropy $s\left(u,v\right)$ follows from stability of the equilibrium state as expressed in (120).

## Appendix C. Local Formulation of Nonequilibrium Thermodynamics

### Appendix C.1. Global and Local Balance Laws

In the body of the paper, we have discussed thermodynamics of systems, only occasionally looking inside the systems, when states are inhomogeneous. For full resolution of what happens inside a system, we need to formulate the conservation laws for mass, momentum and energy, and the non-conservation law for entropy, for each point in space, that is as partial differential equations. For doing so, it is best to first discuss the general structure of balance laws in global and local form, and then specify for the individual quantities to be balanced (mass, momentum, energy, entropy).

We consider a system of fixed volume $V_{0}$. The outer surface of the system is denoted by $\partial V_{0}$, and $n_{i}$ denotes the normal vector on the boundary, pointing outwards. Following Reference [5], we use index notation for vectors and tensors.

A global balance law considers the change in time of a global quantity

(A7) $$\Psi =\int_{V_{0}}\rho \psi dV\;,$$

where ψ is the mass specific property to the the global property Ψ.

The change in time dΨ/dt can be effected by: (a) a convective flux, that is the amount of Ψ that is transferred in or out of the system when mass crosses the system boundary,

(A8) $$\dot{\Psi }=-\oint_{\partial V_{0}}\rho \psi V_{i}n_{i}dA\;;$$

here, dA is a surface element of the boundary $\partial V_{0}$ and $-\rho V_{i}n_{i}dA$ is the amount of mass crossing dA during a time interval dt.

(b) a non-convective flux with local flux vector $\varphi_{i}$, so that the overall amount flowing over the system boundary is

(A9) $$\dot{\Phi }=-\oint_{\partial V_{0}}\varphi_{i}n_{i}dA\;;$$

(c) a production Π inside the system with local production density π,

(A10) $$\Pi =\int_{V_{0}}\pi dV\;;$$

and (d) a supply Θ with local supply density ρθ to the bulk of the system

(A11) $$\Theta =\int_{V_{0}}\rho \theta dV\;.$$

The difference between supply and production is that a supply can, at least in principle, be controlled from the outside, while a production cannot be controlled, and is due to the processes inside the system.

Combining the above into the balance law gives

(A12) $$\frac{d\Psi }{dt}=\dot{\Psi }+\dot{\Phi }+\Pi +\Theta \;.$$

With the Gauss divergence theorem, $\oint_{\partial V_{0}}\gamma_{i}n_{i}dA=\int_{V_{0}}\frac{\partial \gamma_{k}}{\partial r_{k}}dV$, the flux terms can be converted into volume integrals, and since the system volume is fixed, the time derivative can be moved into the integral, so that the balance assumes the form

$$\int_{V_{0}}\left(\frac{\partial \rho \psi }{\partial t}+\frac{\partial \left(\rho \psi V_{k}+\varphi_{k}\right)}{\partial r_{k}}-\pi -\rho \theta \right)dV=0\;.$$

The integral must vanish for arbitrary system volumes, hence the integrand must vanish as well. As a result, we obtain the general form of a balance law in local formulation,

(A13) $$\frac{\partial \rho \psi }{\partial t}+\frac{\partial \left(\rho \psi V_{k}+\varphi_{k}\right)}{\partial r_{k}}=\pi +\rho \theta \;.$$

### Appendix C.2. Local Conservation Laws

For the balance of mass, we have ψ=1. Mass is conserved (π=0), and can only be transferred by convection, that is there is neither a non-convective flux ($\varphi_{k}=0$), nor a supply (θ=0). Hence, the local mass balance reads

(A14) $$\frac{\partial \rho }{\partial t}+\frac{\partial }{\partial r_{k}}\left[\rho V_{k}\right]=0\;.$$

For momentum, we have $\psi =V_{i}$. Momentum is conserved (π=0), it can be transferred to the system by convection, and by forces $\varphi_{k}=-t_{ik}n_{i}$ acting on the system boundary; body forces $G_{i}$, such as gravity, serve as a supply ($\theta =G_{i}$):

(A15) $$\frac{\partial \left[\rho V_{i}\right]}{\partial t}+\frac{\partial }{\partial r_{k}}\left[\rho V_{i}V_{k}-t_{ik}\right]=\rho G_{i}\;.$$

Here, $t_{ij}$ is the symmetric stress tensor defined such that $t_{ik}n_{i}$ is the force on a fluid surface element with normal vector $n_{i}$; in equilibrium the stress tensor reduces to the pressure, $t_{ik|Eq}=-p\delta_{ik}$.

For energy, we have $\psi =u+\frac{1}{2}V^{2}$. Also energy is conserved (π=0). It can be transferred to the system by convection, by non-convective heat flux $q_{k}$, and by the power of the surface forces $-t_{ik}n_{k}V_{i}$; the supply is due to the power $\rho G_{i}V_{i}$ of body forces. Hence the energy balance reads

(A16) $$\frac{\partial }{\partial t}\left[\rho \left(u+\frac{1}{2}V^{2}\right)\right]+\frac{\partial }{\partial r_{k}}\left[\rho \left(u+\frac{1}{2}V^{2}\right)V_{k}+q_{k}-t_{ik}V_{i}\right]=\rho G_{i}V_{i}\;.$$

The above Equations (A14)–(A16) are valid for any fluid or gas. Integrating the mass balance (A14) and the energy balance (A16) over the (time dependent) system volume *V* results in the system conservation laws (122) and (126), with the mass flow over an open section *A* (i.e., an inflow or outflow) of the boundary as

(A17) $$\dot{m}=\int_{A}\rho \left|V_{k}n_{k}\right|dA\;,$$

and total heat and work given by

(A18) $$\dot{Q}=-\oint_{\partial V}q_{i}n_{i}dA\;\;\;,\;\;\;\dot{W}=-\int_{\partial V,\mathrm{solid}}t_{ik}V_{k}n_{i}dA\;;$$

here, ∂V is the boundary of the system, and $\left(\partial V,\mathrm{solid}\right)$ is the solid part of the system boundary, where no mass can cross. The derivation requires some assumptions, including that properties at in/outflows can be replaced by averages, and that the body force has a time-independent potential, $G_{i}=-\frac{\partial \left(g_{n}z\right)}{\partial r_{i}}$.

In the form given above, the conservation laws (A14)–(A16) do not form a closed system of equations for the variables $\left(\rho ,V_{i},T\right)$, since they contain internal energy *u*, heat flux $q_{i}$ and stress tensor $t_{ij}$, which must be related to the variables by means of constitutive relations—this will be discussed in the next section.

For the constitutive theory based on the assumption of local thermodynamic equilibrium, it is most convenient to rewrite the conservation laws with the material time derivative $\frac{D}{Dt}=\frac{\partial }{\partial t}+V_{k}\frac{\partial }{\partial r_{k}}$. After some manipulation, including use of mass balance to simplify momentum and energy balance, and momentum balance (after scalar product with $V_{i}$) to simplify the energy balance, the result can be written as

(A19) $$\begin{array}{ccc} \frac{D\rho }{Dt}+\rho \frac{\partial V_{k}}{\partial r_{k}} & = & 0\;,\\ \rho \frac{DV_{i}}{Dt}-\frac{\partial t_{ik}}{\partial r_{k}} & = & \rho G_{i}\;,\\ \rho \frac{Du}{Dt}+\frac{\partial q_{k}}{\partial r_{k}} & = & -t_{ik}\frac{\partial V_{i}}{\partial x_{k}}\;. \end{array}$$

The last equation is the balance of internal energy, which is not a conservation law, due to the term on the right which describes production of internal energy due to frictional heating.

### Appendix C.3. Linear Irreversible Thermodynamics

The question of finding constitutive equations for $u,q_{i},t_{ij}$, and entropy *s*, and the construction of the entropy balance, is the main question in *Nonequilibrium Thermodynamics*. For strong nonequilibrium, this is a rather difficult topic, with many competing, or complimentary, ideas. The approach for the case of *local thermodynamic equilibrium*, however, is now well established as *Linear Irreversible Thermodynamics* (LIT) [18].

The basic idea is to assume that the property relations for equilibrium are valid locally, for any element of the fluid, that is we have the thermal and caloric equations of state, $p\left(T,\rho \right),\;u\left(T,\rho \right)\;$ and the Gibbs equation, $Tds=du-\frac{p}{\rho^{2}}d\rho$ valid in a volume element dV located at $\vec{r}$.

For the construction of the local balance law for entropy—the 2nd law—we recall that the Gibbs equation was derived for a closed system, that is, it is valid for a material element. Therefore its material time derivative assumes the form

(A20) $$T\frac{Ds}{Dt}=\frac{Du}{Dt}-\frac{p}{\rho^{2}}\frac{D\rho }{Dt}\;,$$

which is the base for the construction of the entropy balance. Replacing the time derivatives of ρ,u by means of the conservation laws, multiplying with $\frac{\rho }{T}$, and an integration by parts, yields

(A21) $$\rho \frac{Ds}{Dt}+\frac{\partial }{\partial r_{k}}\left[\frac{q_{k}}{T}\right]=\sigma \;,$$

with

(A22) $$\sigma =q_{k}\frac{\partial \frac{1}{T}}{\partial r_{k}}+\frac{1}{T}t_{\langle ik\rangle }\frac{\partial V_{\langle i}}{\partial r_{k\rangle }}+\frac{1}{T}\left(p+\frac{1}{3}t_{rr}\right)\frac{\partial V_{k}}{\partial r_{k}}\;.$$

Here, $t_{\langle ik\rangle }$ denotes the trace free and symmetric part of the stress tensor, and $t_{rr}$ is its trace.

Equation (A21) is the local balance of entropy with the non-convective entropy flux $\phi_{k}=\frac{q_{k}}{T}$, and the entropy production density σ. Integration over the system volume yields the system balance (69) that was derived under the assumption of local thermal equilibrium, with the overall entropy generation rate ${\dot{S}}_{gen}=\int_{V_{0}}\sigma dV$.

The requirement of non-negative entropy generation, ${\dot{S}}_{gen}\geq 0$, for all choices of the system volume $V_{0}$ implies non-negative values for the production density, that is, σ≥0. This is a strong condition on the constitutive equations for $q_{i},t_{ik}$. We recognize σ in (A22) as the product of the thermodynamic fluxes $F_{A}=\left\{q_{i},t_{\langle ik\rangle },\left(p+\frac{1}{3}t_{rr}\right)\right\}$ and thermodynamic driving forces, viz. the space derivatives of velocity $V_{i}$ and temperature *T*. Note that the forces describe deviations from equilibrium states, which have homogeneous temperature and velocity.

LIT assumes a linear relationship between fluxes and forces, such that corresponding forces and fluxes have the same tensorial degree (Curie principle) [18]. The result is the stress tensor of the Navier-Stokes equations, and Fourier’s law for the heat flux,

(A23) $$\left(p+\frac{1}{3}t_{rr}\right)=\nu \frac{\partial V_{k}}{\partial r_{k}}\;\;\;,\;\;\;t_{\langle ik\rangle }=2\mu \frac{\partial V_{\langle i}}{\partial r_{k\rangle }}\;\;\;,\;\;\;q_{i}=-\kappa \frac{\partial T}{\partial r_{i}}\;.$$

Here, ν>0 is the bulk viscosity, μ>0 is the shear viscosity, and κ>0 is the heat conductivity; all three must be measured as functions of $\left(\rho ,T\right)$. The signs ensure that the entropy generation is always positive. Note that the viscosities must be positive since thermodynamic temperature is positive—once more we see that positive temperature guarantees mechanical stability.

The entropy generation rate becomes

(A24) $$\sigma =\frac{\kappa }{T^{2}}\frac{\partial T}{\partial r_{i}}\frac{\partial T}{\partial r_{k}}+\frac{2\mu }{T}\frac{\partial V_{\langle i}}{\partial r_{k\rangle }}\frac{\partial V_{\langle i}}{\partial r_{k\rangle }}+\frac{\nu }{T}\frac{\partial V_{j}}{\partial r_{j}}\frac{\partial V_{k}}{\partial r_{k}}\geq 0\;.$$

In summary, LIT derives the Navier-Stokes-Fourier equations that are routinely used in thermodynamics, fluid mechanics, and heat transfer. Moreover, LIT provides the expression for the local entropy balance, which opens the door to full analysis of thermodynamic losses—recall that ${\dot{W}}_{loss}=T_{0}{\dot{S}}_{gen}=T_{0}\int \sigma dV$.

The entropy generation rate (A24) is quadratic in gradients of temperature and velocity. This indicates that processes with small gradients have very small entropy generation, with the limit of reversible processes for vanishing gradients.

### Appendix C.4. Jump and Slip at Boundaries

It is a common assumption that a fluid at a wall assumes the temperature and velocity of the wall. To re-examine boundary conditions for fluids, we consider a wall-fluid boundary with a normal vector $n_{k}$, pointing from the wall into the fluid. For this section properties of the wall are denoted with a superscript *W*, while properties with a superscript *F* denote fluid properties *directly at the wall*.

The wall does not allow fluid to pass, hence the normal velocity of the fluid relative to the wall vanishes,

(A25) $$\left(V_{k}^{F}-V_{k}^{W}\right)n_{k}=0\;.$$

Since momentum and energy are conserved, their non-convective fluxes must be continuous, that is

(A26) $$-t_{ik}^{F}n_{k}=-t_{ik}^{W}n_{k}\;\;\;,\;\;\;q_{k}^{F}n_{k}-t_{ik}^{F}V_{i}^{F}n_{k}=q_{k}^{W}n_{k}-t_{ik}^{W}V_{i}^{W}n_{k}\;.$$

It must be expected that the interaction of fluid and wall is irreversible, so that the entropy flux into the fluid is larger than the flux out of the wall. With the surface entropy production $\bar{\sigma }\geq 0$, we write

(A27) $$\frac{q_{k}^{F}n_{k}}{T^{F}}=\frac{q_{k}^{W}n_{k}}{T^{W}}+\bar{\sigma }\;.$$

To obtain boundary conditions for the fluid, we eliminate the stress and heat flux in the wall from the conservation conditions, to find

(A28) $$\bar{\sigma }=\frac{q_{k}^{F}n_{k}}{T^{F}}-\frac{q_{k}^{W}n_{k}}{T^{W}}=\left(\frac{1}{T^{F}}-\frac{1}{T^{W}}\right)q_{k}^{F}n_{k}+\left(V_{i}^{F}-V_{i}^{W}\right)\frac{t_{ik}^{F}n_{k}-n_{i}t_{jk}^{F}n_{j}n_{k}}{T^{W}}\geq 0\;.$$

Again we require non-negative production at all states, and, employing the LIT constitutive expressions, find expressions for the temperature jump and the velocity slip,

(A29) $$T^{F}-T^{W}=\lambda_{T}\frac{\partial T}{\partial r_{k}}n_{k}\;\;\;,\;\;\;V_{i}^{F}-V_{i}^{W}=\lambda_{V}\left(\frac{\partial V_{\langle i}}{\partial r_{k\rangle }}-\frac{\partial V_{\langle j}}{\partial r_{k\rangle }}n_{i}n_{j}\right)n_{k}\;.$$

Here, $\lambda_{T}>0$ and $\lambda_{V}>0$ are the jump and slip length, respectively, which must be obtained from experiments.

For most engineering applications, $\lambda_{T}$ and $\lambda_{V}$ are so small that they can be set to zero, which yields the common boundary conditions that the fluid sticks to the wall, and has its boundary temperature, $V_{i}^{F}=V_{i}^{W}$, $T^{F}=T^{W}$. The above derivation shows, however, that jump and slip are the natural expectation, since they imply irreversibility at the wall. In particular for rarefied gases, jump and slip might be marked effects [17]. Note that in nonequilibrium systems temperature jumps are expected at thermometer boundaries, so that a thermometer might not show the actual temperature of the fluid.

## Appendix D. Elements of Kinetic Theory of Gases

Much on entropy and the 2nd law can be learned from the kinetic theory of monatomic gases, which we briefly discuss in this Appendix. Kinetic Theory is a fascinating and deep subject, and it should be clear that we can at best give a flavor of its most salient results. For deeper insight, we must refer the reader to the literature, for example, References [15,16,17].

### Appendix D.1. Microscopic Description of a Gas

A gas consists of a huge number—in the order of $10^{23}$—interacting particles α whose physical state is described by their locations $x^{\alpha }=\left\{x_{1}^{\alpha },x_{2}^{\alpha },x_{3}^{\alpha }\right\}$ and their velocities $c^{\alpha }=\left\{c_{1}^{\alpha },c_{2}^{\alpha },c_{3}^{\alpha }\right\}$ at any time *t*. The (micro-) state of the gas is given by the complete set of the $\left\{x^{\alpha },c^{\alpha }\right\}$, and each particle can be described through its trajectory in the 6-dimensional phase space spanned by x and c.

Thus, to describe the gas one could establish the equation of motion for each particle, and then had to solve a set of $∼10^{23}$ coupled equations. Clearly this is not feasible, and therefore kinetic theory chooses to describe the state of the gas on the micro-level through the phase density, or distribution function, $f\left(x,t,c\right)$ which is defined such that $N_{x,c}=f\left(x,t,c\right)dxdc$ gives the number of particles that occupy a cell of phase space dxdc at time *t*. In other words, $N_{x,c}$ is the number of particles with velocities in $\left\{c,c+dc\right\}$ located in the interval $\left\{x,x+dx\right\}$ at time *t*.

With this definition, a certain level of inaccuracy is introduced, since now the state of each particle is only known within an error of dxdc. The phase density $f\left(x,t,c\right)$ is the central quantity in kinetic theory, since the state of the gas is (almost) completely known when *f* is known.

We consider particles of mass *m*. When we integrate mf over velocity, we obtain the mass density ρ. We frequently have to integrate over the full velocity space, and in order to condense notation we write one integral sign without limits,

(A30) $$\rho =m\overset{\infty }{\underset{-\infty }{\;\int \int \int \;}}fdc=m\int fdc\;.$$

The momentum density of the gas is obtained by averaging particle momentum,

(A31) $$\rho V_{i}=m\int c_{i}fdc\;.$$

The peculiar velocity $C_{i}=c_{i}-V_{i}$ of the particles gives the particle speed as measured by an observer moving with the gas at the local velocity $V_{i}$, that is, an observer in the rest-frame, hence the first moment of *f* over $C_{i}$ vanishes,

(A32) $$0=m\int C_{i}fdc\;.$$

The kinetic energy of a particle is given by $\frac{m}{2}c^{2}$, so that the energy density of the gas is given by (*e* denotes the specific energy)

(A33) $$\rho e=\frac{m}{2}\int c^{2}fdc=\frac{m}{2}\int \left(C^{2}+2V_{k}C_{k}+V^{2}\right)fdc=\rho u+\frac{\rho }{2}V^{2}\;,$$

where

(A34) $$\rho u=\frac{m}{2}\int C^{2}fdc\;$$

is the internal, or thermal, energy of the gas, and $\frac{\rho }{2}V^{2}$ is the kinetic energy of its macroscopic motion. Thus, the internal energy of an ideal monatomic gas is the kinetic energy of its particles as measured in the rest-frame.

Pressure tensor $p_{ij}$ and heat flux $q_{i}$ are the fluxes of momentum and energy,

(A35) $$p_{ij}=-t_{ij}=m\int C_{i}C_{j}fdc\;\;\;\mathrm{and}\;\;\;q_{i}=\frac{m}{2}\int C^{2}C_{i}fdc\;.$$

Note that the stress tensor of fluid dynamics is just the negative pressure tensor. Pressure is the trace of the pressure tensor,

(A36) $$p=\frac{1}{3}p_{kk}=\frac{1}{3}m\int C^{2}fdc=\frac{2}{3}\rho u\;,$$

From the ideal gas law p=ρRT follows the relation between energy and temperature as

(A37) $$u=\frac{3}{2}RT\;\;,\;\mathrm{or}\;\;T=\frac{1}{3}\frac{m}{\rho R}\int C^{2}fdc\;.$$

Temperature in kinetic theory is usually defined by the above relation for all situations, including strong deviations from equilibrium.

The atoms in a monatomic gas have three translational degrees of freedom (the three components of velocity), and each degree of freedom contributes $\frac{1}{2}RT$ to the specific internal energy, or $\frac{1}{2}R$ to the specific heat $c_{v}={\left(\frac{\partial u}{\partial T}\right)}_{\rho }$.

### Appendix D.2. Equilibrium and the Maxwellian Distribution

A simple argument going back to Maxwell allows us to find the velocity distribution in equilibrium. Equilibrium is a state where no changes will occur when the gas is left to itself, and this will imply that the gas is homogeneous, that is, displays no gradients in any quantity (external forces such as gravity are ignored), and the phase density is isotropic, that is independent of the direction $\nu_{i}=C_{i}/C$. The following argument considers the gas in the rest-frame, where $V_{i}=0$.

An arbitrary atom picked from the gas will have the velocity components $C_{k},k=1,2,3$, and the probability to find the component in direction $r_{k}$ within the interval $\left[C_{k},C_{k}+dC_{k}\right]$ is given by $\Pi \left(C_{k}\right)dC_{k}$. Note that, due to isotropy, the probability function Π is the same for all components. Then, the probability to find a particle with the velocity vector $\left\{C_{1},C_{2},C_{3}\right\}$ is given by

(A38) $$F\left(C\right)dC_{1}dC_{2}dC_{3}=\Pi \left(C_{1}\right)\Pi \left(C_{2}\right)\Pi \left(C_{3}\right)dC_{1}dC_{2}dC_{3}$$

where $F\left(C\right)=f\left(C\right)/\left(\rho /m\right)$ depends only on the absolute value of velocity, *C*, since the probability must be independent of direction. Thus, *F* and Π are related as

(A39) $$F\left(C\right)=\Pi \left(C_{1}\right)\Pi \left(C_{2}\right)\Pi \left(C_{3}\right)\;.$$

Taking the logarithmic derivative of this equation with respect to $C_{1}$ we see

(A40) $$\frac{\partial }{\partial C_{1}}\ln F\left(C\right)=\frac{\partial }{\partial C_{1}}\left[\ln\Pi \left(C_{1}\right)+\ln\Pi \left(C_{2}\right)+\ln\Pi \left(C_{3}\right)\right]$$

or

(A41) $$\frac{1}{C}\frac{F^{\prime }\left(C\right)}{F\left(C\right)}=\frac{1}{C_{1}}\frac{\Pi^{\prime }\left(C_{1}\right)}{\Pi \left(C_{1}\right)}=-2\gamma \;.$$

Since the left and the right side of this equation depend on different variables, γ must be a constant, and integration gives an isotropic Gaussian distribution,

(A42) $$F=\frac{f}{n}=A\exp\left[-\gamma C^{2}\right]\;\;\mathrm{and}\;\;\Pi \left(C_{k}\right)=\sqrt[{3}]{A}\exp\left[-\gamma C_{k}^{2}\right]\;,$$

where *A* is a constant of integration. The two constants γ and *A* follow from the conditions that the phase density must reproduce mass and energy density, that is

(A43) $$\rho =m\int fdc\;\;\mathrm{and}\;\;\rho u=\frac{3}{2}\rho RT=\frac{m}{2}\int C^{2}fdc\;.$$

The resulting equilibrium phase density is the Maxwellian distribution

(A44) $$f_{M}=\frac{\rho }{m}\frac{1}{{\sqrt{2\pi RT}}^{3}}\exp\left[-\frac{C^{2}}{2RT}\right]\;.$$

### Appendix D.3. Kinetic Equation and Conservation Laws

The evolution of the phase density *f* in space time is given by the Boltzmann equation, which describes the change of *f* due to free flight, external forces $G_{k}$, and binary collisions between particles. Instead of discussing the full Boltzmann equation, we consider a kinetic model equation, known as the BGK model, where the Boltzmann collision term is replaced by a relaxation term,

(A45) $$\frac{\partial f}{\partial t}+c_{k}\frac{\partial f}{\partial r_{k}}+G_{k}\frac{\partial f}{\partial c_{k}}=-\frac{1}{\tau }\left(f-f_{M}\right)\;.$$

Here, τ is the mean free time, that is, the average time a particle travels freely between two collisions with other particles, and $f_{M}$ is the local Maxwell distribution. The right hand side describe the change of the distribution due to collisions, which move the distribution function closer towards the Maxwellian. If the collision frequency 1/τ is relatively large, than we expect that the local state is close to the Maxwellian at all times, which implies local equilibrium. The discussion further below will give more details on this particular limit.

While not exact, the BGK model shares the main features of the Boltzmann equation, and is used in the present brief sketch because of its simplicity.

To simplify notation, we write moment of the phase density as

(A46) $$\rho \langle \psi \rangle =m\int \psi fdc\;,$$

where ψ is any function of $\left(r,t,c\right)$, so that, for example, $\rho =\rho \langle 1\rangle$, $\rho v_{i}=\rho \langle c_{i}\rangle$, $\rho e=\frac{\rho }{2}\langle c^{2}\rangle$, and so forth. The evolution equation for $\langle \psi \rangle$, the equation of transfer, is obtained by multiplying (A45) with $\psi \left(r,t,c\right)$, and subsequent integration,

(A47) $$\frac{\partial \rho \langle \psi \rangle }{\partial t}+\frac{\partial \rho \langle \psi c_{k}\rangle }{\partial r_{k}}=\rho \langle \frac{\partial \psi }{\partial t}\rangle +\rho \langle c_{k}\frac{\partial \psi }{\partial r_{k}}\rangle +\rho \langle G_{k}\frac{\partial \psi }{\partial c_{k}}\rangle +S_{\psi }\;.$$

The production term $S_{\psi }$ is defined as

(A48) $$S_{\psi }=-\frac{1}{\tau }\int \psi \left(f-f_{M}\right)dc\;,$$

with no productions for the conserved quantities mass, momentum and energy,

(A49) $$S_{1}=S_{c_{i}}=S_{c^{2}}=0\;,$$

that is the Boltzmann equation, and the BGK model, guarantee conservation of mass, momentum, and energy.

For $\psi =\left\{1,c_{i},\frac{1}{2}c^{2}\right\}$, (A47) and the above definitions of macroscopic properties imply the conservation laws for mass, momentum and energy (A14)–(A16).

### Appendix D.4. Entropy and 2nd Law in Kinetic Theory

In the equation of transfer (A47) we chose, following Boltzmann [15,16,17],

(A50) $$\psi =-k_{B}\ln\frac{f}{y}$$

with the Boltzmann constant $k_{B}$ and another constant *y*. We introduce

(A51) $$\eta =-k_{B}\int f\ln\frac{f}{y}dc\;\;,\;\;\Phi_{\kappa }=-k_{B}\int c_{k}f\ln\frac{f}{y}dc$$

and

(A52) $$\Sigma =S_{-k_{B}\ln\frac{f}{y}}=\frac{k_{B}}{\tau }\int \ln\frac{f}{y}\left(f-f_{M}\right)dc=\frac{k_{B}}{\tau }\int \ln\frac{f}{f_{M}}\left(f-f_{M}\right)dc\;,$$

so that the corresponding transport equation for η reads

(A53) $$\frac{\partial \eta }{\partial t}+\frac{\partial \Phi_{k}}{\partial r_{k}}=\Sigma \;.$$

Closer examination shows that the collision term (A52) cannot be negative, and Σ vanishes in equilibrium, so that

(A54) Σ≥0.

This feature of the BGK model reproduces the behavior of the full Biolzmann collision term. Thus, η always has a positive production which vanishes in equilibrium. Accordingly, η can only grow in an isolated system, where no flux over the surface is allowed ($\oint_{\partial V}\Phi_{k}n_{k}dA=0$), and reaches its maximum in equilibrium, where Σ=0. This property of η and in particular the definite sign of Σ are known as the H-theorem.

In our view, the H-theorem is equivalent to the second law of thermodynamics, the entropy law, which was introduced above on purely phenomenological grounds. Then, η is the entropy density of the gas, and we write

(A55) η=ρs,

where *s* denotes the specific entropy. $\Phi_{\kappa }$ as given in (A51)${}_{2}$ is the entropy flux, and the non-convective entropy flux $\phi_{k}=\Phi_{k}-\rho sv_{k}$ can be computed according to

(A56) $$\phi_{\kappa }=-k_{B}\int C_{k}f\ln\frac{f}{y}dc\;.$$

This gives the second law in the form

(A57) $$\rho \frac{Ds}{Dt}+\frac{\partial \phi_{k}}{\partial x_{k}}=\Sigma \geq 0\;;$$

Σ is the entropy production (entropy generation rate).

The specific entropy of the monatomic gas in equilibrium follows by evaluating (A51)${}_{1}$ with the Maxwellian (A44) as

(A58) $$s=R\ln\frac{T^{3/2}}{\rho }+s_{0}\;\;\mathrm{where}\;\;s_{0}=R\left[\frac{3}{2}+\ln\left(mR^{3/2}y{\sqrt{2\pi }}^{3}\right)\right]\;.$$

This result stands in agreement with classical thermodynamics.

Phase density and Boltzmann equation are valid for arbitrary nonequilibrium states, hence the above expressions for entropy, entropy flux, and entropy generation, and the balance law (A57) hold for arbitrary nonequilibrium states. There is no restriction of Boltzmann entropy to equilibrium states!

The Boltzmann equation is constructed such that entropy η has a strictly non-negative production rate Σ≥0. This is not in agreement with the behavior of microscopic mechanical systems. The instantaneous reversal of the microscopic velocities of all particles should send the gas back to its initial state, which would imply destruction of entropy (Loschmidt’s reversibility paradox). Also, any mechanical system will, after some time, return to (microscopic) states arbitrarily close to its initial state, with an entropy close to that of the initial state, which also implies destruction of entropy (Zermelo’s recurrence paradox).

The reversibility paradox is resolved by observing that the overwhelming majority of possible microscopic initial conditions (for the same macroscopic state) will result in processes with non-negative entropy generation. Microscopic states that result from sudden inversion of all particle velocities belong to the comparatively rather small number of possible initial conditions that lead to entropy destruction. Indeed, the relative number of such initial conditions must be so small that these can be ignored—after all, observation of macroscopic processes shows non-negative entropy generation. Also, it must be noted that it is impossible to realize the sudden reversal of all microscopic velocities.

The recurrence paradox is resolved by observing that the recurrence time is so long (much longer than the lifetime of the universe for systems with many particles) that entropy destruction would not be observed during our lifetimes.

In summary, we can state that the Boltzmann equation describes the probable behavior of gases, with non-negative generation of entropy. For a deeper discussion of these points we must refer the reader to References [1,15,16,17].

### Appendix D.5. Kinetic Theory in the Continuum Limit

To align the idea of local thermodynamic equilibrium with kinetic theory, we use the Chapman-Enskog method to determine an approximate phase density from the kinetic equation. The approach presented is justified for flows where the Knudsen number is sufficiently small [16,17]. The Knudsen number is defined as the ratio of average mean free path $\lambda =\tau \sqrt{RT}$ of the gas and the relevant length scale of a process, as $\mathrm{Kn}=\frac{\tau \sqrt{RT_{0}}}{L}$, where we use $\sqrt{RT_{0}}$ with a meaningful reference temperature $T_{0}$ as a measure of average particle speed in the restframe.

In dimensionless form, with scales based on *L*, $T_{0}$, the BGK collison term appears as $\frac{1}{\mathrm{Kn}}\left(f-f_{M}\right)$, and evaluation of the Boltzmann equation can be performed in the limit of small Knudsen numbers. Instead, one can work with the dimensional equation, and introduce a formal scaling parameter ε that plays the role of the Knudsen number in taking limits. The parameter will be set to unity at the end of the scaling procedure. Hence, we write the kinetic equation with the convective time derivative as

(A59) $$\frac{Df}{Dt}+C_{k}\frac{\partial f}{\partial r_{k}}+G_{k}\frac{\partial f}{\partial c_{k}}=-\frac{1}{\varepsilon }\frac{1}{\tau }\left(f-f_{M}\right)\;.$$

The idea of the Chapman-Enskog expansion is to write *f* as a series in ε (or, in the Knudsen number Kn, if the dimensionless equation is used),

(A60) $$f=f^{\left(0\right)}+\varepsilon f^{\left(1\right)}+\varepsilon^{2}f^{\left(2\right)}+\cdots$$

The expansion terms $f^{\left(\alpha \right)}$ are determined from inserting the series into the Boltzmann equation, and evaluation of the contributions at the different powers of ε. Here, we are only interested in the first two contributions. Inserting the ansatz into (A59), and equating terms of equal powers in $\varepsilon^{-1}$ and $\varepsilon^{0}$ yields at first

(A61) $$0=-\frac{1}{\tau }\left(f^{\left(0\right)}-f_{M}\right)\;\;\;,\;\;\;\frac{Df^{\left(0\right)}}{Dt}+C_{k}\frac{\partial f^{\left(0\right)}}{\partial r_{k}}+G_{k}\frac{\partial f^{\left(0\right)}}{\partial c_{k}}=-\frac{1}{\tau }f^{\left(1\right)}\;.$$

Hence, the leading order term is the local Maxwellian, $f^{\left(0\right)}=f_{M}$, and the first order correction $f^{\left(1\right)}$ is determined from the Maxwellian. Using the conservation laws for eliminating the ensuing time derivatives, one finds

(A62) $$f^{\left(1\right)}=-f_{M}\tau \left[\frac{C_{\langle k}C_{l\rangle }}{\theta }\frac{\partial v_{\langle k}}{\partial r_{l\rangle }}+C_{k}\left(\frac{C^{2}}{2\theta }-\frac{5}{2}\right)\frac{1}{\theta }\frac{\partial \theta }{\partial r_{k}}\right]\;.$$

When the first order approximation of the distribution function, $f^{\left(0\right)}+\varepsilon f^{\left(1\right)}$, is inserted into (A35) to determine stress tensor and heat flux to first order, one finds just the laws of Navier-Stokes and Fourier,

(A63) $$t_{ij}^{\left(1\right)}=-p\delta_{ij}+2p\tau \frac{\partial v_{\langle i}}{\partial r_{k\rangle }}\;\;\mathrm{and}\;\;q_{i}^{\left(1\right)}=-\frac{5}{2}p\tau \frac{\partial \theta }{\partial r_{i}}\;,$$

where viscosity and thermal conductivity for the BGK model are identified as

(A64) $$\mu =p\tau \;\;\mathrm{and}\;\;\kappa =\frac{5}{2}p\tau \;.$$

We note that the BGK model gives an incorrect Prandtl number, $\mathrm{Pr}=\frac{5}{2}\frac{\mu }{\kappa }=1$, while the full Boltzmann equation yields the proper value of $\mathrm{Pr}≃\frac{2}{3}$. Moreover, the monatomic gas has no bulk viscosity.

According to the CE expansion, the first order solution for the phase density can be written as

(A65) $$f=f_{M}\left(1+\varepsilon \varphi \right)\;,$$

and the corresponding expressions for entropy and its flux follow from first order expansion of the logarithm, $\ln\left(1+\varepsilon \varphi \right)≃\varepsilon \varphi$. For the entropy we obtain to first order in ε

(A66) $$\rho s=-k\int f_{M}\left(1+\varepsilon \varphi \right)\left(\ln\frac{f_{M}}{y}+\varepsilon \varphi \right)dc=-k\int f_{M}\ln\frac{f_{M}}{y}dc\;.$$

Since the first order contributions vanish, this is just the entropy in equilibrium (24). We conclude that to first order in ε the gas can be described locally as if it were in equilibrium. This result gives justification to the hypothesis of local thermodynamic equilibrium.

The corresponding first order entropy flux is obtained as

(A67) $$\phi_{\kappa }=-k\int C_{k}f_{M}\varepsilon \varphi \left(\ln\frac{f_{M}}{y}+1\right)dc\;.$$

Since $\ln f_{M}+1=-C^{2}/2\theta +F\left(\rho ,T\right)$, and $\int C_{k}f_{M}\varphi dc=0$, we obtain, again after setting the formal parameter ε=1, an approximation for the entropy flux to first order in ε,

(A68) $$\phi_{\kappa }=\frac{1}{T}\frac{m}{2}\int C_{k}C^{2}f_{M}\varphi dc=\frac{q_{k}}{T}\;.$$

Also this relation between heat flux and entropy flux is well-known in classical thermodynamics. The corresponding entropy generation rate Σ is the same as in LIT, given in (A24).

### Appendix D.6. Entropy as S = k*lnΩ*

Boltzmann’s choice (A50) allows to relate entropy to microscopic properties, and to interpret entropy as a measure for disorder [5,17].

We consider a gas of *N* particles in the volume *V*, and compute its total entropy as

(A69) $$H=\int_{V}\eta dr=-k\int_{V}\int f\ln\frac{f}{y}dcdr\;.$$

The number of particles in a phase cell is

(A70) $$N_{r,c}=fdrdc=fY\;,$$

where Y=drdc is the size of the cell. By writing sums over phase cells instead of integrals, and with $N=\sum_{r,c}N_{r,c}$ follows

(A71) $$H=-k\sum_{r,c}N_{r,c}\ln\frac{N_{r,c}}{yY}=-k\sum_{r,c}N_{r,c}\ln N_{r,c}+kN\ln yY\;.$$

Use of Stirling’s formula, lnN!=NlnN−N, yields

(A72) $$H=-k\sum_{r,c}\left(\ln N_{r,c}!+N_{r,c}\right)+kN\ln yY=k\ln\frac{N!}{\prod_{r,c}N_{r,c}!}+kN\ln\frac{yY}{N}\;.$$

Choosing y=N/Y finally allows to write

(A73) H=klnΩ

where

(A74) $$\Omega =\frac{N!}{\prod_{r,c}N_{r,c}!}$$

is the number of possibilities to distribute *N* particles into the cells of phase space, so that the cell at $\left(r,c\right)$ contains $N_{r,c}$ particles.

Equation (A73) relates the total gas entropy *H* to the number of possibilities to realize the state of the gas. The growth of entropy, which is imperative in an isolated system, therefore corresponds to an increasing number of possibilities to realize the state. Since a small number of possibilities refers to an ordered state, and a large number to disorder, we can say that the H-theorem states that disorder must grow in an isolated process. Accordingly, entropy is often interpreted as a measure for disorder.

The relation (A73) is generally accepted as being valid not only for monatomic ideal gases—where it originated—but for any substance. However, the evaluation for other substances can be quite difficult, or impossible, since it requires a detailed understanding of the microscopic details of phase space, accessible states, and so forth, in order to determine Ω properly.

Finally we note that we used the symbols η,H to denote Boltzmann entropies, but that we consider these to be the actual entropies of the gas, that is η=ρs,H=∫ηdV=∫ρsdV=S.
